# METTL3-mediated m6A modification of has_circ_0007905 promotes age-related cataract progression through miR-6749-3p/EIF4EBP1

**DOI:** 10.7717/peerj.14863

**Published:** 2023-03-06

**Authors:** Rui Li, Haohao Zhu, Qian Li, Jiancen Tang, Yiping Jin, Hongping Cui

**Affiliations:** 1 Department of Ophthalmology, Shanghai East Hospital, School of Medicine, Tongji University, Shanghai, China; 2 Department of Ophthalmology, The Fifth People’s Hospital of Shanghai, Fudan University, Shanghai, China

**Keywords:** Age-related cataract, METTL3, has_circ_0007905, miR-6749-3p, EIF4EBP1, m6A modification

## Abstract

Many cases of blindness are caused by age-related cataracts (ARCs). N6-methyladenosine (m6A)-modified circRNA widely participates in disease progression. However, the role of m6A modification of circRNA in ARC is unclear. We mined and elucidated the functions and mechanisms of key circRNAs with m6A modification involved in ARC progression. The GSE153722 dataset was used to mine m6A-mediated key circRNA. Loss-of-function assays and rescue assays were used to explore the effect and mechanism of circRNA on ARC cell proliferation and apoptosis. Has_circ_0007905 was a hypermethylated and upregulated expression in the ARC group relative to the control group both* in vivo* and *in vitro*. Silencing of has_circ_0007905 promoted proliferation and inhibited the apoptosis of HLE-B3 cells. METTL3 was upregulated in HLE-B3 cells after ARC modeling and had four binding sites with has_circ_0007905 and a mediated m6A modification of has_circ_0007905. Proliferation was significantly inhibited and apoptosis of HLE-B3 cells was facilitated by METTL3 overexpression, whereas these effects were prevented by has_circ_0007905 silencing. Silencing of has_circ_0007905 led to an alteration in the transcriptome landscape. Differentially expressed genes were mainly involved in immune-related processes and pathways. EIF4EBP1 overexpression promoted apoptosis and suppressed proliferation, and also significantly reversed effects of has_circ_0007905 silencing. Moreover, miR-6749-3p significantly decreased the luciferase activities of wild type plasmids with both of has_circ_0007905 and EIF4EBP1. MiR-6749-3p inhibitor blocked elevation in proliferation and reduced EIF4EBP1 expression and apoptosis conferred by has_circ_0007905 silencing. We reveal for the first time that the commitment of ARC progression is guided by METTL3/has_circ_0007905/miR-6749-3p/EIF4EBP1 axis, and the results provide new insights into ARC pathology.

## Introduction

Age-related cataract (ARC) is classified into three subtypes based on the different regions of opacity within the lens: posterior subcapsular, cortical, and nuclear (Shiels & Hejtmancik, 2017). Currently, ARC contributes to the most significant number of blindness cases caused by cataracts worldwide and is strongly associated with age (Lawrence, Fedorowicz & Van Zuuren, 2015). However, the dual challenges of an increasingly aging population and treatment reliance on cataract surgery have called for the development of new and effective therapeutic strategies for ARC.

N6-methyladenosine (m6A) refers to the insertion of a methyl substituent on adenosine at the N6 position. It most prevalently occurs in the stop codons and 3′ untranslated region with a conserved motif of RRACH (R = G or A; and H = A, C, or U) (Luo et al., 2022). The m6A modification process includes m6A installation by “writers” (m6A methyltransferases), m6A removal by “erasers” (m6A demethylases), and m6A recognition by “readers” (recognition proteins) (Chen & Wong, 2020). Benefiting from m6A involvement in regulating RNA translation, splicing, and stability, m6A plays an essential biological role in a variety of diseases and cellular functions (Chen et al., 2022), including cancer (Jing et al., 2021) and neurologic disorders (Yen & Chen, 2021). It has been shown in a recent review that m6A modification also plays a vital role in the pathogenesis of diabetic retinopathy (Kumari et al., 2021). It was suggested in a report on transcriptome-wide m6A methylome sequencing of the anterior lens capsule of high myopia patients that m6A may modulate the composition of the extracellular matrix by proteins that alter fundus anatomy in high myopia (Wen et al., 2021). Overall, m6A plays a critical role in ocular diseases. Furthermore, methylase METTL3 repressed proliferation and promoted apoptosis of human lens epithelial cells in diabetic cataracts (Yang et al., 2020), indicating that cataract pathological progression is related to m6A modification. However, the mechanism by which m6A is involved in ARC progression is still largely unknown.

Intriguingly, abundant m6A modifications in circRNAs similar to mRNA have been proven in emerging studies (Liu et al., 2022a). CircRNAs are a class of stable and ubiquitous noncoding RNAs highly conserved in mammals. The latest study reported that the m6A abundance of total circRNAs was reduced in the lens epithelium cells of ARC patients. m6A regulators, such as METTL14, WTAP, and ALKBH5, were significantly upregulated in ARC tissue compared to the normal lens (Li et al., 2020). It is suggested by these results that m6A may participate in ARC by regulating circRNAs. However, the study of circRNA RNA methylation is still at an early stage, and the pathogenesis of the m6A modification of circRNA in ARC has not yet been reported.

In this study, we intended to screen the critical circRNAs in ARC through MeRIP-seq and sequencing data of circRNAs and clarify the function of candidate circRNAs in lens epithelial cells through cellular experiments. Finally, transcriptome sequencing was used to explore the downstream molecular mechanisms of the circRNAs. For the first time, we reveal the role and molecular mechanism of m6A modification of circRNAs in ARC progression.

## Material and Methods

### Data downloaded from the Gene Expression Omnibus (GEO) database

The circRNA-seq data and MeRIP-seq data were downloaded from the GEO database under accession number GSE153722 (six ARC *vs.* six normal). The differential expression analysis of circRNA-seq and MeRIP-seq was performed using the R package DESeq under the cut-off criteria: —log2FC—≥ 1. The m6A-enriched circRNA in the normal control and ARC samples were analyzed. The intersection of differentially expressed circRNAs and differentially methylated m6A-enriched circRNAs served as candidate circRNAs.

Additionally, the RNA-protein binding site between has_circ_0007905 and METTL3 was predicted using the RBPsuite software (http://www.csbio.sjtu.edu.cn/bioinf/RBPsuite/). The potential m6A modification sites of has_circ_0007905 were predicted using SRAMP software (http://www.cuilab.cn/sramp).

### Sample collection of crystalline lens from ARC patients

Crystalline lens samples were obtained from conventional continuous curvilinear capsulorhexis in ARC patients (*n* = 10, each sample with three replicates), with no vascular contact or damage to the iris or other intraocular tissues. Patients with complex cataracts due to high myopia, trauma, uveitis, glaucoma, or other systemic diseases, such as hypertension and diabetes, were excluded from the study. Transparent lenses removed from normal subjects with shallow anterior chambers served as controls (*n* = 2, each sample with three replicates). The “shallow anterior chamber” is only a potential risk factor for glaucoma, but there is currently no related disease, and it also belongs to normal people. In this group, the clinical option was to deepen the anterior chamber through preventive lens surgery to remove the potential glaucoma risk. Signed informed consent was obtained from all patients. Detailed clinical data for each individual human subject are shown in Table S1. This study was approved by the Ethics Committee of Shanghai East Hospital, School of Medicine, Tongji University ([2021] Audit Research No. 79).

### RT-qPCR analysis

Total RNA was extracted from crystalline lens samples and HLE-B3 cells using TRIzol (Invitrogen). The concentration and purity of total RNA were measured using a microspectrophotometer (Tiangen, Beijing, China). High-quality RNA was stored at −80 °C for subsequent RT-qPCR and RNA sequencing. Next, reverse transcription was conducted using a RevertAid™ First Strand cDNA Synthesis Kit (K16225; Thermo Fisher, Waltham, MA, USA). cDNA amplification was performed by 2 × PCR Master Mix (Roche, Basel, Switzerland) on the ABI Q6 Flex Real-time PCR system (Applied Biosystems, Foster City, CA, USA). Gene expression was calculated using the 2^−ΔΔCT^ method. All primers used in this study are shown in Table S2.

### Cell culture and ultraviolet B (UVB) irradiation and transfection

The human lens epithelial cell line of HLE-B3 cells was purchased from BFB BLUEFBIO (BFN60805970; Bluebio (Yantai) Bio-Pharmaceutical Co., Ltd., Yantai, China) and cultured in high glucose DMEM (10-013-CVRC; Corning, Shanghai, China) supplemented with 10% FBS (10099, GIBCO) and 1% PS (E607011, Sangon). HLE-B3 cells were maintained in an incubator with a 5% CO_2_ atmosphere at 37 °C. To construct ARC cell model, HLE-B3 cells were exposed to UVB light (from top to bottom) for 10 min, according to a previous study, with slight modifications in exposure time (Xiang et al., 2019).

For the knockdown of has_circ_0007905 and METLL3, siRNAs were designed and synthesized by GenePharma. The has-miR-6749-3p mimic and has-miR-6749-3p inhibitor were also synthesized by GenePharma to overexpress and knockdown the has-miR-6749-3p, respectively. For overexpression of EIF4EBP1, the full length of EIF4EBP1 was cloned into pCDNA3.1 vectors (OE-EIF4EBP1), and the blank vector served as NC.

Transfection was conducted using Lipofectamine 2000 reagent (Invitrogen) according to the manufacturer’s instructions. Briefly, when the fusion rate of the cells reached 80–90%, the fresh medium was replaced. All siRNA, vectors, and Lipofectamine 2000 reagents were diluted with OPTI-MEM (5 µL:45 µL). Next, diluted Lipofectamine and RNA sequences were mixed for 20 min and added to the cell sample.

### MeRIP-qPCR analysis

Isolated RNA was purified and fragmented into fragments with ∼200 bp using RNA Fragmentation Reagent (Invitrogen, Waltham, MA, USA). Around 10% of the volume of fragmented RNA was set aside as an input group. Magnetic beads A/G were mixed with anti-m6A antibodies and set aside. Next, fragmented RNA was incubated with the prepared anti-m6A antibody in an immunoprecipitation buffer, and anti-immunoglobulin G (IgG) served as a negative control. After incubation, RNA was eluted from the beads and subjected to RT-qPCR analysis.

### CCK8 assay

The proliferation of HLE-B3 cells was assessed using a CCK-8 kit (C0037; Beyotime Biotechnology, Shanghai, China). Briefly, HLE-B3 cells were processed into single-cell suspensions with a density of 1 × 10^4^ cells/mL and were seeded into a 6-well plate with 1000 cells/well. Each sample set had six replicates. At the indicated times of 0, 24, 48, 72, and 96 h, 10 µL CCK-8 solution was added to each well. The absorbance value at 450 nm was detected after 1 h.

### Flow cytometry

At 6 h post-transfection, the medium was replaced with fresh medium. The cells were cultured for 48 h for flow cytometry. The cells were harvested and gently washed with PBS. Cells were resuspended with 195 µL of 1 × Binding Buffer to a cell density of 2–5 × 10^5^ cells/mL. A total of 5 µL of Annexin V-FITC (C1062; Beyotime, Shanghai, China) was added to the cell resuspension and incubated for 15 min at room temperature in the dark. Next, the cells were washed with 200 µL 1 × Binding Buffer and centrifuged at 1000 rpm for 5 min to remove the supernatant. Finally, the cells were resuspended in 190 µL of 1 × Binding Buffer, and 10 µL of propidium iodide was added. A flow cytometry assay was performed within 4 h.

### TUNEL analysis

The apoptosis of HLE-B3 cells was assessed with the One Step TUNEL Apoptosis Assay Kit (green fluorescence) (C1086; Beyotime Biotechnology, Shanghai, China). Briefly, HLE-B3 cells were fixed with 4% paraformaldehyde for 30 min and then washed with PBS, followed by permeabilization in PBS containing 0.3% Triton X-100 for 5 min. Afterward, 50 µL TUNEL solution was added to the HLE-B3 cells and incubated for 60 min in the dark. Lastly, HLE-B3 cells were sealed with antifluorescence quenching sealing tablets and photographed on a fluorescence microscope.

### Transcriptome sequencing

RNA extracted from HLE-B3 cells after has_circ_0007905 knockdown (*n* = 3) or NC (*n* = 3) was used for transcriptome sequencing. RNA sequencing was performed by Yingbio Technology (Shanghai, China) on an Illumina HiSeq 2500 platform. The statistical power of this experimental design was calculated in RNASeqPower and was 0.896. The raw data were filtered using the FastQC method. Clean reads were subjected to screening of differentially expressed genes (DEGs) using the DEGSeq algorithm upon thresholds of Log2FC >1 or <-1 and FDR <0.05. GO and KEGG enrichment were analyzed on DEGs. The RNA-seq data generated in this study are available in the Sequence Read Archive under accession number PRJNA868911.

### Western blot

HLE-B3 cells were harvested and lysed using a lysis buffer, and the protein concentration of cell extracts was quantified with a BSA kit. Next, appropriately 20 µg protein was loaded on 10 SDS-PAGE gels and transferred into PVDF membranes, followed by blocking with TBST solution containing 5% skim milk at 4 °C for 3 h. Membranes were incubated with primary antibodies at 4 °C overnight and then incubated with secondary antibodies of Goat Anti-Mouse IgG H&L (HRP) (1:1000, ab205719; Abcam, Cambridge, UK) and Goat Anti-Rabbit IgG H&L (HRP) (1:20000, ab6721; Abcam, Cambridge, UK). Finally, the protein bands were imaged using the Bio-Rad ChemiDoc XRS system. Primary antibodies, including GAPDH (1:2000, 60004-1-Lg; Proteintech, Rosemont, IL, USA), METTL3 (1:1000, 15073-I-AP; Proteintech), and EIF4EBP1 (1:2000, ab32024; Abcam, Cambridge, UK), were used.

### RNA immunoprecipitation (RIP)

The RIP assay was performed in this study using the Magna RIP RNA-Binding Protein IP Kit (Millipore, Burlington, MA, USA) according to the product instruction. Cells were collected and washed twice with pre-cooled PBS and centrifuged at 1500 rpm for 5 min at 4 °C to discard supernatant, followed by adding RIP Lysis Buffer to lyse the cells on ice for 5 min. Diluted 50 µL of magnetic beads in 100 µL of RIP Wash Buffer was used. The sample was incubated with 5 µg of Argonaute-2 antibody or 1 µg IgG antibody for 30 min at room temperature with rotation. After light centrifugation, the supernatant was removed, and the precipitate was resuspended in 0.5 mL RIP Wash Buffer. After that, 900 µL of RIP Immunoprecipitation Buffer was added to the bead-antibody mixture and then incubated with 100 cell lysis products overnight at 4 °C with rotation. Finally, 150 µL proteinase K buffer was added to the complex product and incubated at 55 °C for 30 min, followed by RNA extraction for RT-qPCR.

### Dual-luciferase activity assay

HLE-B3 cells were seeded into 96-well plates and co-transfected with has-miR-6749-3p mimic/NC and psiCHECK™-2 vectors containing has_circ_0007905 wild type (WT) or mutant (Mut) 3′ UTR sequence, and psiCHECK™-2 vectors containing EIF4EBP1 WT or Mut 3′ UTR sequence. At 48 h after transfection, the cells were lysed and incubated with firefly luciferase buffer to detect firefly luciferase activity, followed by incubation with Renilla luciferase to detect firefly luciferase activity (Promega, Madison, WI, USA).

### Statistical analysis

Data analysis was executed using GraphPad Prism 9.0. The *t*-test was used to assess significant differences between two groups, and one-way analysis of variance (ANOVA) following Turkey’s test to assess significant differences among three groups. All data are displayed as mean ± standard deviation (mean ± SD), and a *P*-value lower than 0.05 was considered significant.

## Results

### Identification of hypermethylated-upregulated expression of circRNAs

**Figure 1:**
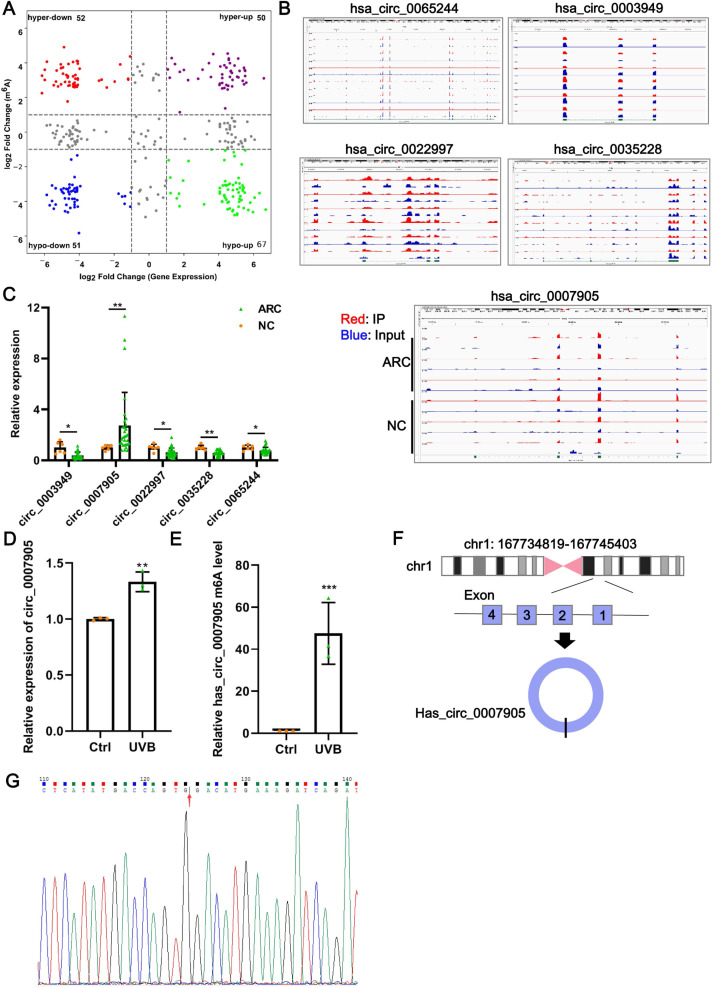
Has_circ_0007905 promotes apoptosis and inhibits the proliferation of lens epithelial cells. (A) Four-quadrant volcano plot. The abscissa represents the differentially expressed circRNAs, and the ordinate represents the differentially methylated circRNAs. (B) The distribution of m6A peaks of five circRNAs. m6A enrichment was shown by immunoprecipitation (IP) in red, and the input control was in blue. (C) The expression of five candidate circRNAs in clinical ARC tissue (*n* = 10) and transparent lenses (*n* = 3) was measured using RT-qPCR. (D) The expression of has_circ_0007905 in HLE-B3 cells after UVB exposure was measured using RT-qPCR. (E) The m6A level of has_circ_0007905 in HLE-B3 cells after UVB exposure was measured using MeRIP-qPCR. (G) Sanger sequencing was performed to verify the back-splicing site of has_circ_0007905. (F) Diagram illustrating that has_circ_0007905 is generated from back-splicing between exons 1 and 4 of chromosome 1. ns indicates *P* > 0.05, an asterisk (*) indicates *P* < 0.05, two asterisks (**) indicate *P* < 0.01, three asterisks (***) indicate *P* < 0.001.

Data from GSE153722 were analyzed to screen differentially expressed circRNAs and differentially methylated circRNAs. According to GSE153722 circRNA-seq data, a total of 220 differentially expressed circRNAs were identified, including 117 upregulated and 103 downregulated in the ARC group compared to the NC group (Fig. 1A). According to GSE153722 MeRIP-seq data, a total of 220 differentially methylated circRNAs were identified, including 102 hypermethylation circRNAs and 118 hypomethylation circRNAs in the ARC group compared to the NC group (Fig. 1A). Subsequently, as shown in Fig. 1A, there were 50 overlapping circRNAs that satisfied the hypermethylation upregulated in the ARC group. These overlapping circRNAs have attracted our attention. To further narrow the scope of candidate hypermethylated-upregulated expression circRNAs, five circRNAs (has_circ_0007905, has_circ_0065244, has_circ_0003949, has_circ_0022997, and has_circ_0035228) with the most significantly changed were selected for validation. The distribution of m6A peaks of the five circRNAs is shown in Fig. 1B, and they all harbored more than three m6A peaks. We also examined the expression of five candidate circRNAs in ARC tissue using RT-qPCR. Compared with the NC group, only the expression of has_circ_0007905 (*P* = 0.00114) was upregulated in the ARC group, whereas the remaining four circRNA expressions were opposite (Fig. 1C). Next, we determined the expression pattern and m6A level of has_circ_0007905 in ARC cell model induced by UVB exposure. As expected, a significant increase of has_circ_0007905 expression in HLE-B3 cells after UVB exposure was observed (Fig. 1D; *P* = 0.00292). The total m6A level of has_circ_0007905 was also significantly elevated in the UVB treatment group compared with the control group (Fig. 1E; *P* = 0.00543). According to the annotation in UCSC Genome Browser on Human (GRCh37/hg19) (https://genome.mdc-berlin.de/index.html), has_circ_0007905 was formed by the back-splicing of exons 1 and 4 (Fig. 1F). The back-splicing site of has_circ_0007905 was confirmed with Sanger sequencing (Fig. 1G). Collectively, we obtained the dataset of hypermethylated-upregulated expression circRNAs in ARC tissue and focused on has_circ_0007905.

### Silencing of has_circ_0007905 promotes proliferation and inhibits apoptosis of lens epithelial cells

**Figure 2:**
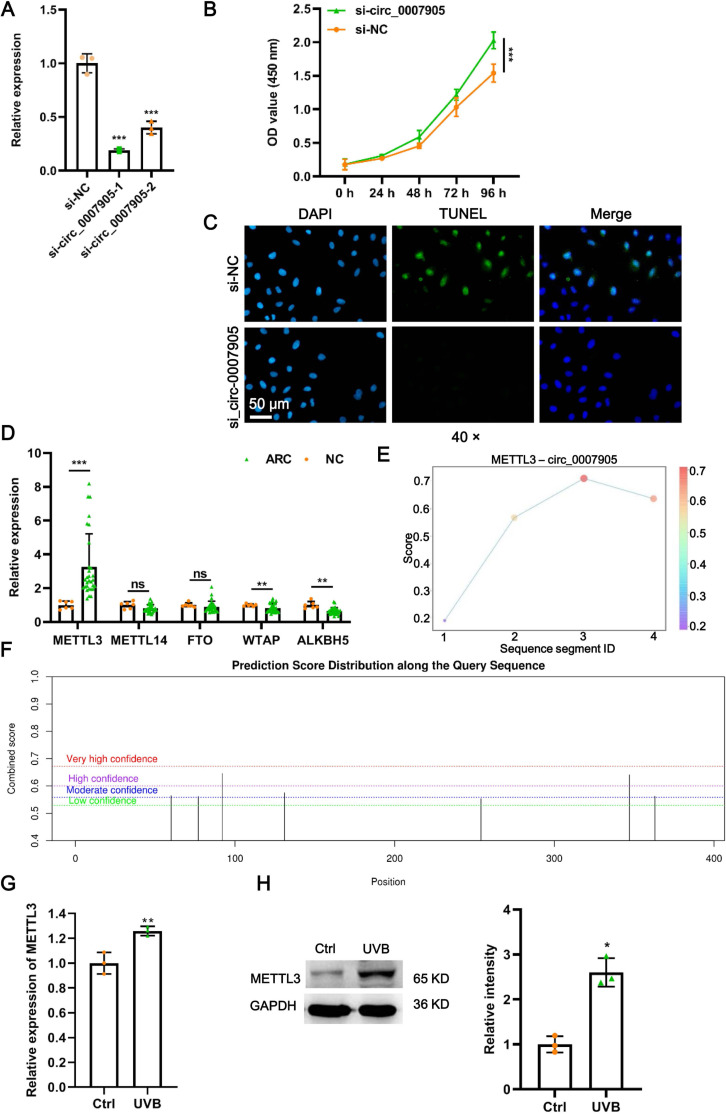
METTL3 mediates the m6A modification of has_circ_0007905. (A) The silencing efficacy of has_circ_0007905 was confirmed by RT-qPCR. (B) The proliferation of HLE-B3 cells after silencing of has_circ_0007905 was measured by CCK-8 assays. (C) The apoptosis of HLE-B3 cells after silencing of has_circ_0007905 was measured by TUNEL staining. (D) The expression of five candidate m6A modification enzymes in clinical ARC tissue (*n* = 10) and transparent lenses (*n* = 3) were measured using RT-qPCR. (E) Four RNA-protein binding sites between has_circ_0007905 and METTL3 were predicted using the RBPsuite database. (F) Seven potential m6A modification sites of has_circ_0007905 were predicted by SRAMP. The mRNA (G) and protein (H) expression of METTL3 in HLE-B3 cells after UVB exposure was measured using RT-qPCR and western blot, respectively. ns indicates *P* > 0.05, an asterisk (*) indicates *P* < 0.05, two asterisks (**) indicate *P* < 0.01, three asterisks (***) indicates *P* < 0.001.

To further investigate the function of has_circ_0007905 in ARC progression *in vitro*, we interfered with has_circ_0007905 expression in the HLE-B3 cell line using two siRNA sequences. We confirmed that has_circ_0007905 expression was significantly reduced upon the presence of siRNA, especially siRNA-1 (si-has_circ_0007905-1; *P* < 0.0001) (Fig. 2A). Meanwhile, CCK-8 assays indicated that silencing of has_circ_0007905 significantly boosted the proliferation of HLE-B3 cells (Fig. 2B; *P* < 0.0001). TUNEL staining demonstrated that apoptosis of HLE-B3 cells was significantly decreased after silencing of has_circ_0007905 compared with the NC group (Fig. 2C). These results highlight that has_circ_0007905 promotes the progression of ARC *in vitro*.

### METTL3 mediates m6A modification of has_circ_0007905

As we all know, m6A modification is driven by the m6A writer composed of METTL3, METTL14, and WTAP, which are removed by m6A erasers, such as FTO and ALKBH5 (Deng et al., 2018). To further examine which enzyme is engaged in m6A modification of has_circ_0007905, we detected the expression of METTL3, METTL14, WTAP, FTO, and ALKBH5 in ARC tissues. Intriguingly, only METTL3 expression was significantly upregulated in ARC tissues compared to the NC group (Fig. 2D; *P* < 0.0001). Four RNA-protein binding sites between has_circ_0007905 and METTL3 (Fig. 2E) were predicted by the RBPsuite database (http://www.csbio.sjtu.edu.cn/bioinf/RBPsuite/). Seven potential m6A modification sites in has_circ_0007905 were also expected using SRAMP (http://www.cuilab.cn/sramp) (Fig. 2F). Moreover, the mRNA (*P* = 0.00882) and protein (*P* = 0.00162) expressions of METTL3 were significantly elevated in HLE-B3 cells upon UVB exposure (Figs. 2G and 2H). Thus, we initially guessed that the m6A modification of has_circ_0007905 was mediated by METTL3.

### METTL3 inhibits proliferation and promotes apoptosis of lens epithelial cells *via* has_circ_0007905

**Figure 3:**
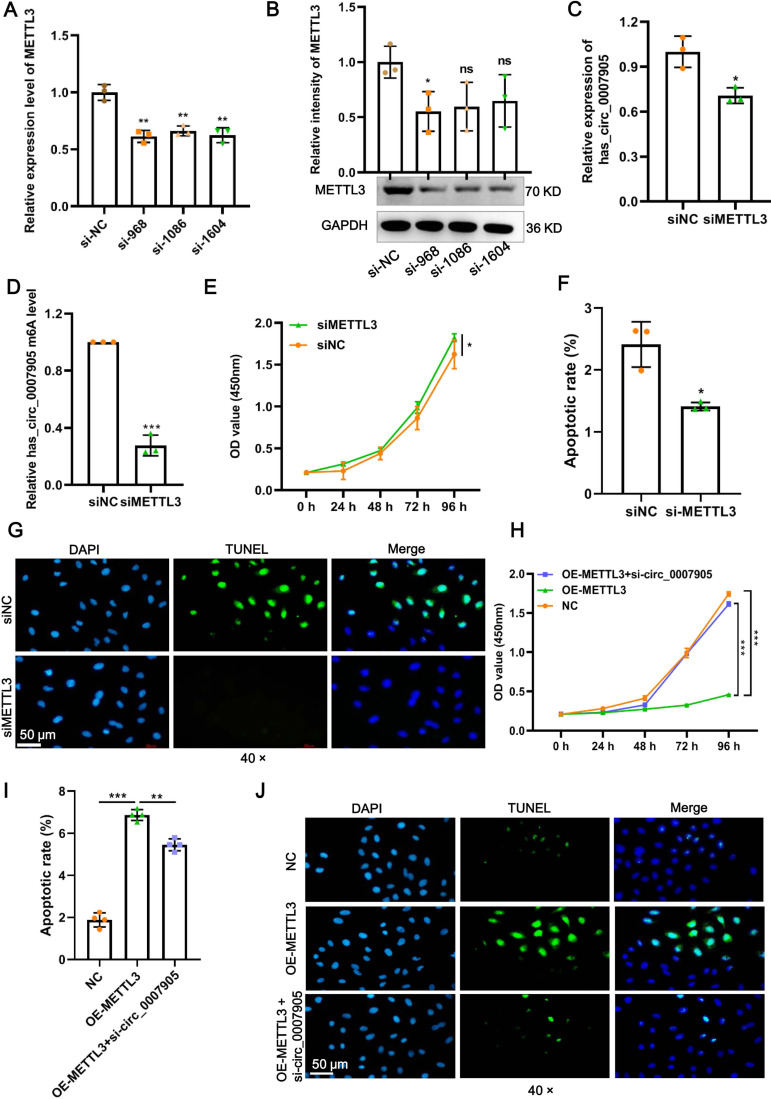
METTL3 inhibits proliferation and promotes apoptosis *via* has_circ_0007905 in lens epithelial cells. (A–B) Knockdown efficacy of METTL3 was confirmed by RT-qPCR and western blot. (C) The expression of has_circ_0007905 in HLE-B3 cells after the knockdown of METTL3 was measured using RT-qPCR. (D) The m6A level of has_circ_0007905 in HLE-B3 cells after the knockdown of METTL3 was measured using MeRIP-qPCR. (E) The proliferation of HLE-B3 cells after silencing METTL3 was measured by CCK-8 assays. The apoptosis of HLE-B3 cells after silencing of METTL3 was measured by flow cytometry (F) and TUNEL staining (G). The effect of simultaneous METTL3 overexpression and silencing of has_circ_0007905 on the proliferation (H) and apoptosis (I–J) of HLE-B3 cells was measured using CCK-8 assays, flow cytometry, and TUNEL staining, respectively. An asterisk (*) indicates *P* < 0.05, three asterisks (***) indicate *P* < 0.001.

Four siRNAs against METTL3 were synthesized to explore the functional role of METT L3 in ARC progression *in vitro*. It was shown in the results of RT-qPCR and western blot that the mRNA and protein expression of METTL3 was significantly decreased after transfection with siRNA-968. Thus, siRNA-968 was used for METTL3 knockdown experiments (Figs. 3A and 3B). Expectedly, METTL3 knockdown significantly diminished the expression (Fig. 3C; *P* = 0.012) and m6A modification level (Fig. 3D; *P* < 0.0001) of has_circ_0007905. In addition, METTL3 knockdown significantly facilitated proliferation (Fig. 3E; *P* = 0.0202) and inhibited apoptosis detected by flow cytometric assay (Fig. 3F; *P* = 0.010) (Fig. S1) and TUNEL (Fig. 3G) of HLE-B3 cells. These results suggested that knockdown of METTL3 attenuated ARC progression *in vitro*.

We conducted rescue experiments to further clarify whether the function of METTL3 in ARC progression is has_circ_0007905-dependent. CCK-8 assay demonstrated that the proliferation of HLE-B3 cells was significantly impaired (*P* < 0.0001) by the overexpression of METTL3. However, the proliferation was significantly restored by the simultaneous METTL3 overexpression and silencing of has_circ_0007905 (*P* < 0.0001) (Fig. 3H). In contrast, overexpression of METTL3 significantly accelerated apoptosis of HLE-B3 cells. Still, this phenotypic effect was prevented by has_circ_0007905 silencing, which was detected using flow cytometric assay (Fig. 3I; NC *vs.* OE-METTL3, *P* < 0.0001, OE-METTL3 *vs.* OE-METTL3+ si-circ_0007905, *P* = 0.0071) (Fig. S2) and TUNEL (Fig. 3J). Therefore, we concluded that METTL3 promotes ARC progression *in vitro via* has_circ_0007905 in an m6A-dependent manner.

### Silencing of has_circ_0007905 leads to an alteration in transcriptome landscape in lens epithelial cells

**Figure 4:**
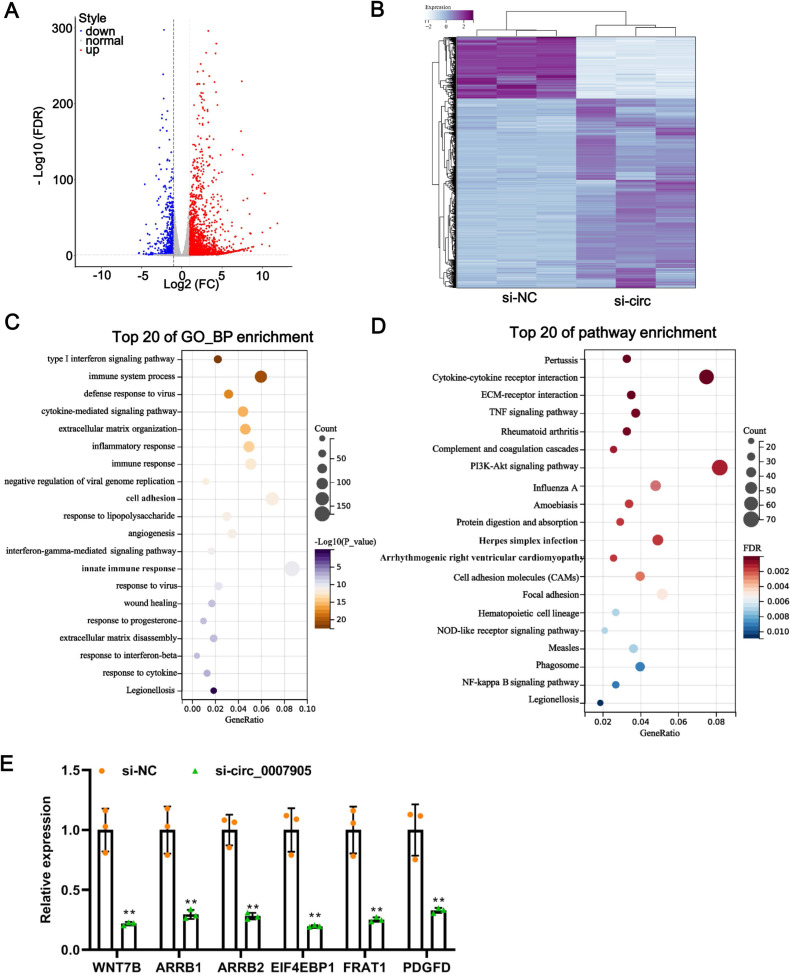
Silencing of has_circ_0007905 leads to an altered transcriptome landscape in lens epithelial cells. Volcano plot (A) and Heat map (B) of the differentially expressed genes (DEGs) between the NC group and the has_circ_0007905 silencing group. (C) GO enrichment analysis based on DEGs. (D) KEGG pathway enrichment analysis based on DEGs. (E) The expression of six DEGs in RNA-Seq was verified by RT-qPCR.

One of the mechanisms of circRNA is to regulate the expression of target genes through the ceRNA mechanism. Thus, we mined the downstream target genes of has_circ_0007905 using transcriptome sequencing. We found that has_circ_0007905 interference resulted in 707 mRNAs downregulation and 2184 mRNAs upregulation compared with the NC group (Fig. 4A). These DEGs were clearly clustered into two branches (Fig. 4B). To investigate the physiological significance of DEGs in ARC progression, GO and KEGG pathway enrichment were performed for DEGs. GO enrichment revealed that these DEGs were mainly associated with the immune-related process, interestingly, 13 of the 20 high-ranking significantly enriched GO entries were related to immune processes, such as the immune system process, inflammatory response, immune response, and innate immune response (Fig. 4C). KEGG pathway enrichment showed that these DEGs were mainly involved in immune-related pathways, including cytokine-cytokine receptor interaction, complement and coagulation cascades, and proliferation-related pathways, such as the PI3K-Akt and NF-kappa B signaling pathways (Fig. 4D). These results implicated that has_circ_0007905 interference may be involved in the progression of ARC by affecting the immune response processes and proliferation-related pathways of HLE-B3 cells through DEGs.

To verify the reliability of the sequencing results, five down-regulated DEGs in the has_circ_0007905 interference group with high abundance and significant *P*-values were selected for RT-qPCR. Compared with the NC group, the expression of these five DEGs was significantly reduced in the has_circ_0007905 interference group (Fig. 4E). Given that ARC manifests as cellular senescence and loss and diminished proliferation, we focused on a target gene associated with proliferation and apoptosis (Yang et al., 2020). EIF4EBP1 has been reported to be associated with proliferation and apoptosis (Zhang & Su, 2022), so we focused on EIF4EBP1.

### Has_circ_0007905 inhibits proliferation and promotes apoptosis *via* EIF4EBP1 in lens epithelial cells

**Figure 5:**
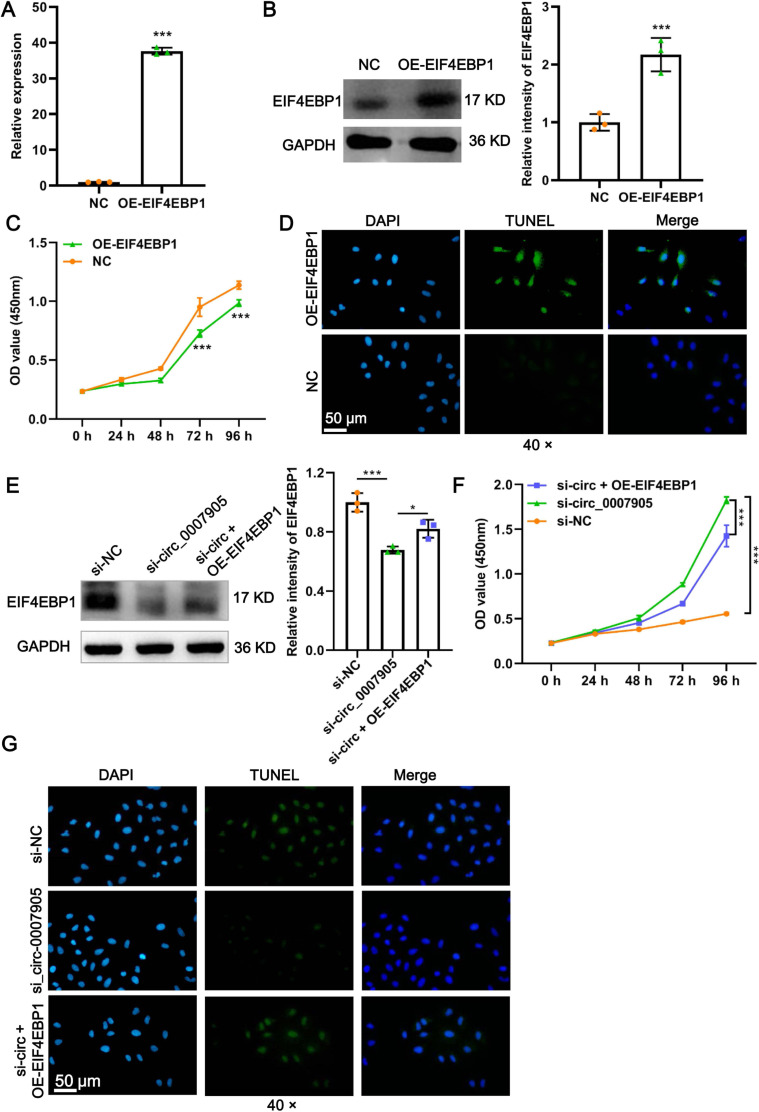
Has_circ_0007905 inhibits proliferation and promotes apoptosis *via* EIF4EBP1 in lens epithelial cells. The mRNA (A) and protein (B) expression of EIF4EBP1 in HLE-B3 cells after overexpression of EIF4EBP1. The proliferation (C) and apoptosis (D) of HLE-B3 cells after overexpression of EIF4EBP1 were measured using CCK-8 assays and TUNEL staining, respectively. The effects of simultaneous METTL3 overexpression and silencing of has_circ_0007905 on EIF4EBP1 protein expression (E), proliferation (F), and apoptosis (G) of HLE-B3 cells were measured using western blot, CCK-8 assays, and TUNEL staining, respectively. An asterisk (*) indicates *P* < 0.05, three asterisks (***) indicate *P* < 0.001.

To explore the function of EIF4EBP1 in ARC progression *in vitro*, we constructed plasmids that overexpressed EIF4EBP1 and transfected them into HLE-B3 cells. A significant increase in EIF4EBP1 expression at mRNA and protein levels was confirmed by RT-qPCR (Fig. 5A; *P* < 0.0001) and western blot (Fig. 5B; *P* = 0.00330) after overexpression of EIF4EBP1. Moreover, we observed that proliferation was significantly prevented (Fig. 5C; *P* < 0.0001) and that apoptosis was enhanced (Fig. 5D) in HLE-B3 cells after EIF4EBP1 overexpression, indicating that EIF4EBP1 contributes to ARC progression *in vitro*. EIF4EBP1 expression was significantly reduced by has_circ_0007905 interference (*P* = 0.0007) and this effect could be reversed with EIF4EBP1 overexpression (*P* = 0.0344) (Fig. 5E). The CCK8 results showed that the excessive proliferation caused by has_circ_0007905 interference was significantly attenuated by EIF4EBP1 overexpression (Fig. 5F; *P* < 0.0001). TUNEL staining proved that has_circ_0007905 interference resulted in the attenuation of apoptosis, which was significantly rescued by EIF4EBP1 overexpression (Fig. 5G). Taken together, these results supported that has_circ_0007905 inhibits proliferation and promotes apoptosis *via* EIF4EBP1 in HLE-B3 cells.

### Has_circ_0007905 inhibits proliferation and promotes apoptosis *via* miR-6749-3 p/EIF4EBP1 in lens epithelial cells

**Figure 6:**
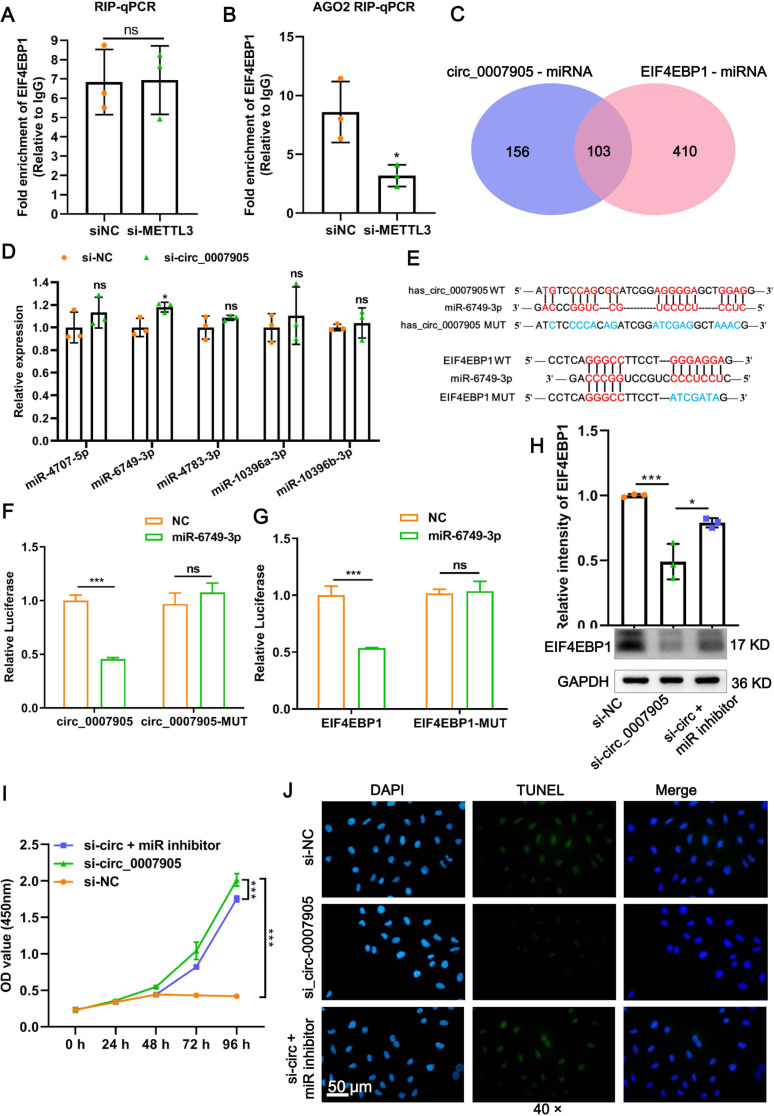
Has_circ_0007905 inhibits proliferation and promotes apoptosis *via* miR-6749-3p/EIF4EBP1 in lens epithelial cells. (A) RIP-qPCR to detect the binding between has_circ_0007905 and EIF4EBP1. (B) AGO2-RIP-qCPR detects whether the binding of has_circ_0007905 and EIF4EBP1 is AGO protein dependent. (C) Venn diagram of the predicted miRNA of has_circ_0007905 and EIF4EBP1. (D) Five miRNAs were selected for RT-qPCR validation. (E) WT binding sites and MUT binding sites of miR-6749-3p and has_circ_0007905/EIF4EBP1. The red font indicates complementary paired bases, and the blue font indicates mutation sites. (F) The binding relationship between has_circ_0007905 and miR-6749-3p was verified using a dual-luciferase reporter assay. (G) The binding relationship between EIF4EBP1 and miR-6749-3p was verified using dual-luciferase reporter assay. The effects of simultaneous has_circ_0007905 silencing and miR-6749-3p inhibitor on EIF4EBP1 protein expression (H), proliferation (I), and apoptosis (J) of HLE-B3 cells were measured using western blot, CCK-8 assays, and TUNEL staining, respectively. ns indicates *P* > 0.05, an asterisk (*) indicates *P* < 0.05, three asterisks (***) *P* < 0.001.

To confirm the mode of interaction between has_circ_0007905 and EIF4EBP1, RIP-PCR and AGO2-dependent AGO2-RIP were performed, respectively. The results showed that the enrichment of EIF4EBP1 in the absence of an AGO2 antibody was not affected by the knockdown of has_circ_0007905 (Fig. 6A; *P* = 0.942). However, the enrichment of EIF4EBP1 was significantly reduced by has_circ_0007905 interference in the presence of an AGO2 antibody (Fig. 6A; *P* = 0.027). These results suggested that EIF4EBP1 is regulated by has_circ_0007905 through an AGO2-dependent ceRNA mechanism.

Given that has_circ_0007905 regulates the expression of EIF4EBP1 through the ceRNA mechanism, we next explored the miRNA that acts as a communication bridge between has_circ_0007905 and EIF4EBP1. According to the RNAhybrid and Miranda databases, has_circ_0007905 and EIF4EBP1 potentially bind 259 and 513 miRNAs, respectively. Among these, 103 miRNAs were shared by has_circ_0007905 and EIF4EBP1 (Fig. 6A). Five miRNAs with the highest binding energy were selected for RT-qPCR validation. It was found that only the expression of miR-6749-3p was significantly upregulated after the knockdown of has_circ_0007905 (Fig. 6B; *P* = 0.0272).

Furthermore, the WT binding sites and MUT binding sites of miR-6749-3p and has_circ_0007905/EIF4EBP1 displayed in Fig. 6C. As shown in Fig. 6D, miR-6749-3p mimic significantly decreased the luciferase activities of has_circ_0007905 in the cells carrying the WT plasmid rather than the MUT plasmid (*P* < 0.0001), also reduced the luciferase activities of EIF4EBP1-WT (*P* = 0.000561) (Fig. 6E). The decrease in EIF4EBP1 expression induced by has_circ_0007905 silencing (*P* = 0.0007) was significantly reversed by the miR-6749-3p inhibitor (*P* = 0.0103) (Fig. 6F). Importantly, has_circ_0007905 silencing conferred that the elevation in the proliferation of HLE-B3 cells was significantly mitigated by the miR-6749-3p inhibitor (Fig. 6G). The effects of has_circ_0007905 silencing on apoptosis were also significantly blocked by the miR-6749-3p inhibitor (Fig. 6H). In summary, these results demonstrated that has_circ_0007905 functions as a sponge for miR-6749-3p to regulate EIF4EBP1 expression to involve the proliferation and apoptosis of HLE-B3 cells.

## Discussion

Emerging studies have proved that the vital role of epigenetic modifications (Li et al., 2020; Wang et al., 2015; Wang et al., 2017) and circRNA (Liang et al., 2020; Liu et al., 2021; Liu et al., 2022b) in the pathogenesis of ARC. However, the role of m6A modification on circRNA during ARC has not been revealed yet. To the best of our knowledge, our study is the first to investigate the function and mechanism of m6A modification in circRNA in ARC. We uncovered that METTL3-mediated m6A modification of has_circ_0007905 promotes apoptosis and inhibits the proliferation of HLE-B3 cells through miR-6749-3p/EIF4EBP1, leading to ARC progression.

METTL3 is an m6A methylase catalytic center (Wang, Doxtader & Nam, 2016). METTL3-mediated m6A modification of RNAs has a non-negligible role in eye-related diseases, such as proliferative vitreoretinopathy (Wang, Doxtader & Nam, 2016) and diabetes-induced pericyte dysfunction (Suo et al., 2022). Currently, limited by the short study period in which circRNA catalyzed by METTL3 for m6A modification can mediate ARC progression has not yet been reported. In 2020, Yang et al. revealed that METTL3 contributes to diabetic cataract progression by stabilizing ICAM-1 mRNA stability to promote the proliferation and apoptosis of HLE cells (Yang et al., 2020). This study affirms the important role of METTL3 in diabetic cataract but not in those of circRNA or ARC. The report by Li et al. was similar to our results. In that study, m6A-tagged circRNAs in ARC were characterized using MeRIP-seq, regrettably, they did not reveal any specific functions or mechanisms of m6A modification on circRNAs (Li et al., 2020). Our findings addressed deficiencies in those two studies. We demonstrated that m6A modified has_circ_0007905 is mediated by METTL3, which promoting apoptosis and inhibiting proliferation in ARC.

In the present study, has_circ_0007905 with hypermethylation and upregulation in ARC tissues relative to control tissue was identified. Has_circ_0007905 was first reported by Huang et al., compared with paired para-cancerous cervical tissues, has_circ_0007905 was upregulated in cervical cancer, suggesting a cancer-promoting potential (Huang, Chen & Huang, 2021). Has_circ_0007905 was produced from the STX6 mRNA located on chr1 (q25, 3) thereby it was termed as circSTX6 by Meng et al. Pancreatic ductal adenocarcinoma progression *in vitro* and *in vivo* in a ceRNA manner through sponging miR-449b-5p was also provoked by has_circ_0007905 (Meng et al., 2022). There is no more research on has_circ_0007905. Still, our conclusion is consistent with those two studies, in which it was shown that has_circ_0007905 has pathogenic potentials. Conclusively, these data indicated that has_circ_0007905 was involved in ARC by promoting apoptosis and inhibiting proliferation of HLE-B3 cells.

Among the mechanisms of circRNA function, ceRNA theory is commonly considered (Qi et al., 2015), this was also the case for has_circ_0007905 in this study. We found that has_circ_0007905 could directly bind miR-6749-3p to release EIF4EBP1 mRNA, and either overexpression of EIF4EBP1 or inhibition of miR-6749-3p could alleviate the effects of has_circ_0007905 silencing on the proliferation and apoptosis of HLE-B3 cells. Except for the fact that miR-6749-3p from serum can be used as a diagnostic biomarker of intervertebral disc degeneration together with miR-766-3p and miR-4632-5p (Cui et al., 2020b), there is no other study on miR-6749-3p. This unique report implicates the engagement of miR-6749-3p in disease progression, and our study enriches our knowledge related to miR-6749-3p. However, more research is needed to support our conclusions. Moreover, EIF4EBP1, the target gene of has_circ_0007905/miR-6749-3p, has been reported to be extensively involved in cell proliferation and apoptosis, such as in acute myeloid leukemia cells (Jiang et al., 2021), ovarian cancer cells (Lee, Kim & Jeon, 2016), and extravillous trophoblast cells (Dong et al., 2022). Nonetheless, the role of EIF4EBP1 in the proliferation and apoptosis of ARC cells has not been reported. Thus, this is the first report on the role of EIF4EBP1 in ARC. This is the first report indicating that the has_circ_0007905/miR-6749-3p/EIF4EBP1 axis is involved in ARC cell proliferation and apoptosis, which will benefit our understanding of ARC pathogenesis.

Some interesting “contradictory results” should be highlighted. Generally, the up-regulated gene is a promoting factor of disease, which should promote cell proliferation. However, in our study, silencing of up-regulated has_circ_0007905 promotes proliferation and inhibits apoptosis of HLE-B3 cells. In fact, this result is not contradictory, because the pathological logic of “Generally” is usually applied to tumors, but does not apply in many diseases, especially in cataracts. In ARC, human lens epithelial cells apoptosis and proliferation inhibition is an initiating element in cataract development. Thus, in many cataract studies, knockdown of upregulated pathogenic genes promotes proliferative and inhibits apoptosis, and these studies support our conclusion. For example, lncRNA TUG1 expression was significantly higher in the anterior lens capsules of ARC than that in the normal anterior lens capsules, and si-TUG1 significantly inhibited apoptosis of human lens epithelial cell line SRA01/04 (Li et al., 2017); similarly, lncRNA TUG1 expression was significantly upregulated in SRA01/04 cells following oxidative stress, and si-TUG1 significantly increased cell viability and reduced levels of apoptosis (Shen & Zhou, 2021). Moreover, circHIPK3 was down-regulated in human lens epithelium samples of ARC patients, overexpression of circHIPK3 mediated the promotion of proliferation and inhibition of apoptosis (Cui, Wang & Huang, 2020a), while silencing of circHIPK3 significantly decreased cell viability and proliferation, and accelerated apoptosis upon oxidative stress in primary cultured human lens epithelial cells (Liu et al., 2018). Similar results were observed in circZNF292 (Xu et al., 2021), circ_0060,144 (Liu et al., 2022b), and circMRE11A_013 (Liu et al., 2021). In short, the result of up-regulated has_circ_0007905 promotes proliferation and inhibits apoptosis of HLE-B3 cells is not a contradictory result.

## Conclusion

In summary, the expression of has_circ_0007905 in HLE-B3 cells was promoted by METTL3-mediated m6A modification of has_circ_0007905. The upregulation of has_circ_0007905 promotes the expression of EIF4EBP1 by sponging miR-6749-3p, ultimately promoting apoptosis and inhibiting the proliferation of HLE-B3 cells and leading to ARC progression. The present study breaks down the existing knowledge boundaries and contributes to the development of circRNA-based ARC-targeting drugs.

##  Supplemental Information

10.7717/peerj.14863/supp-1 Supplemental Information 1 METTL3 knockdown significantly inhibited apoptosis detected by flow cytometric assay

10.7717/peerj.14863/supp-2 Supplemental Information 2 Flow cytometry was used to detect the effect of overexpression of METTL3 with simultaneous silencing of has_circ_0007905 on the apoptosis of HLE-B3 cells

10.7717/peerj.14863/supp-3 Supplemental Information 3 Detailed clinical data for each individual human subject

10.7717/peerj.14863/supp-4 Supplemental Information 4 The primers used in this study

10.7717/peerj.14863/supp-5 Supplemental Information 5 Raw data

## Additional Information and Declarations

### Data Availability

The following information was supplied regarding data availability: The RNA-seq data generated in this study is available in the Sequence Read Archive (SRA): PRJNA868911. The raw data is available in the Supplemental File.
