# Supplementary figures and images for: METTL3-mediated m6A modification of has_circ_0007905 promotes age-related cataract progression through miR-6749-3p/EIF4EBP1
Source: PeerJ. 2023 Mar 6;11:e14863. doi: 10.7717/peerj.14863 (PMC9997201; doi:10.7717/peerj.14863)

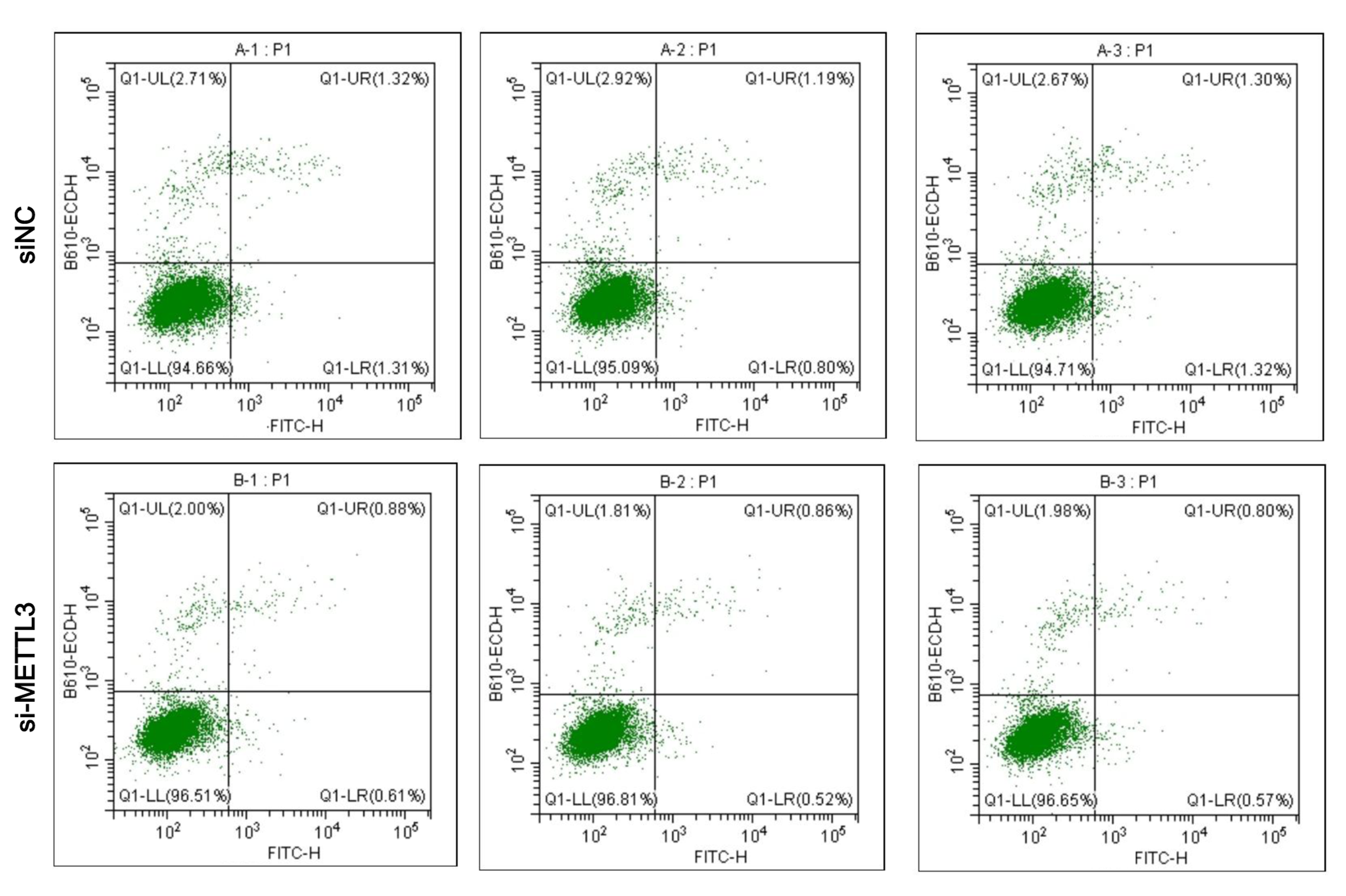

Supplement: Supplemental Information 1 [file peerj-11-14863-s001.tif]

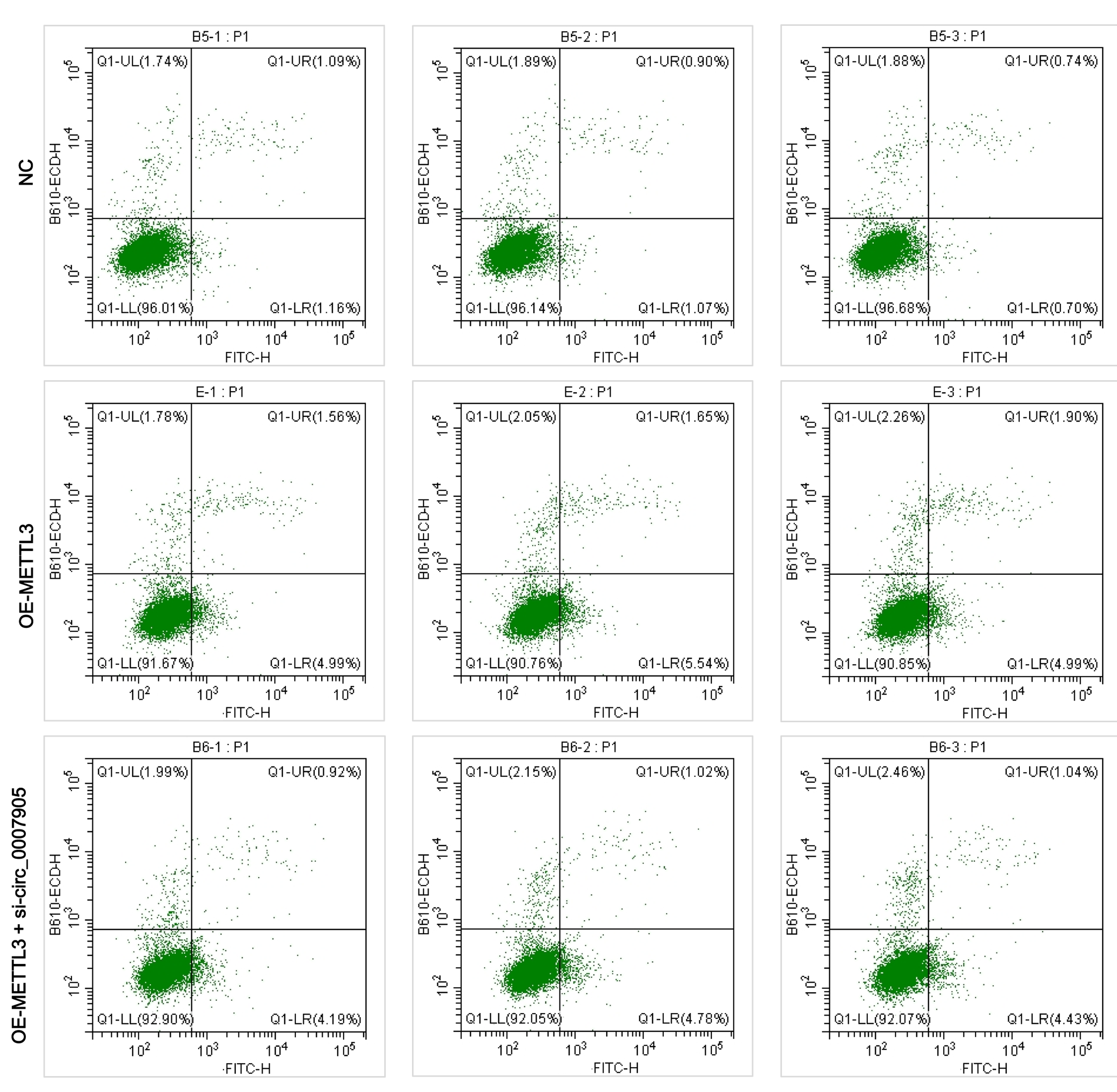

Supplement: Supplemental Information 2 [file peerj-11-14863-s002.tif]

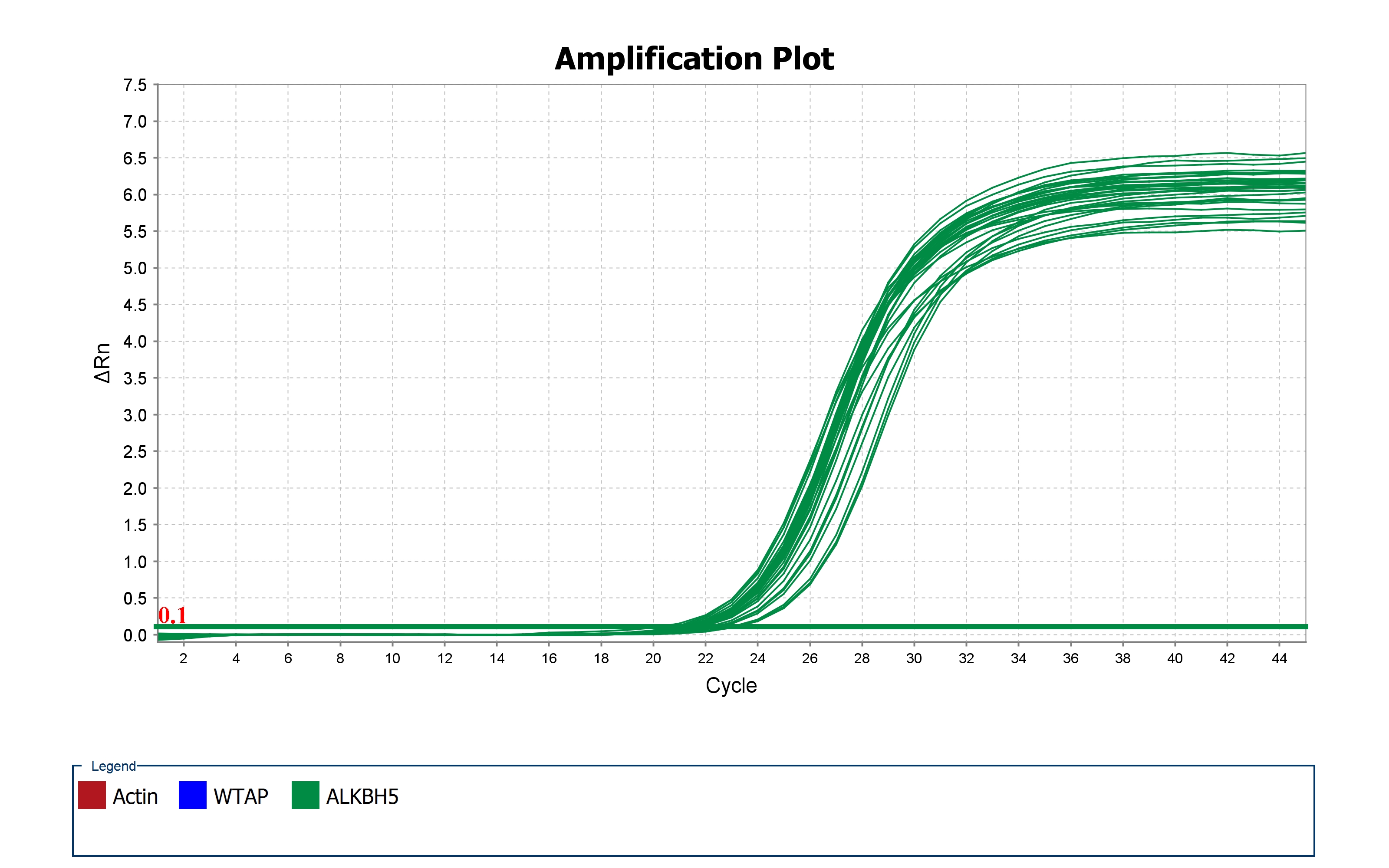

Supplement: Supplemental Information 5 [file peerj-11-14863-s005.zip › Raw data/Fig 1C and 2D/raw data/Amplification Plot ALKBH5.jpg]

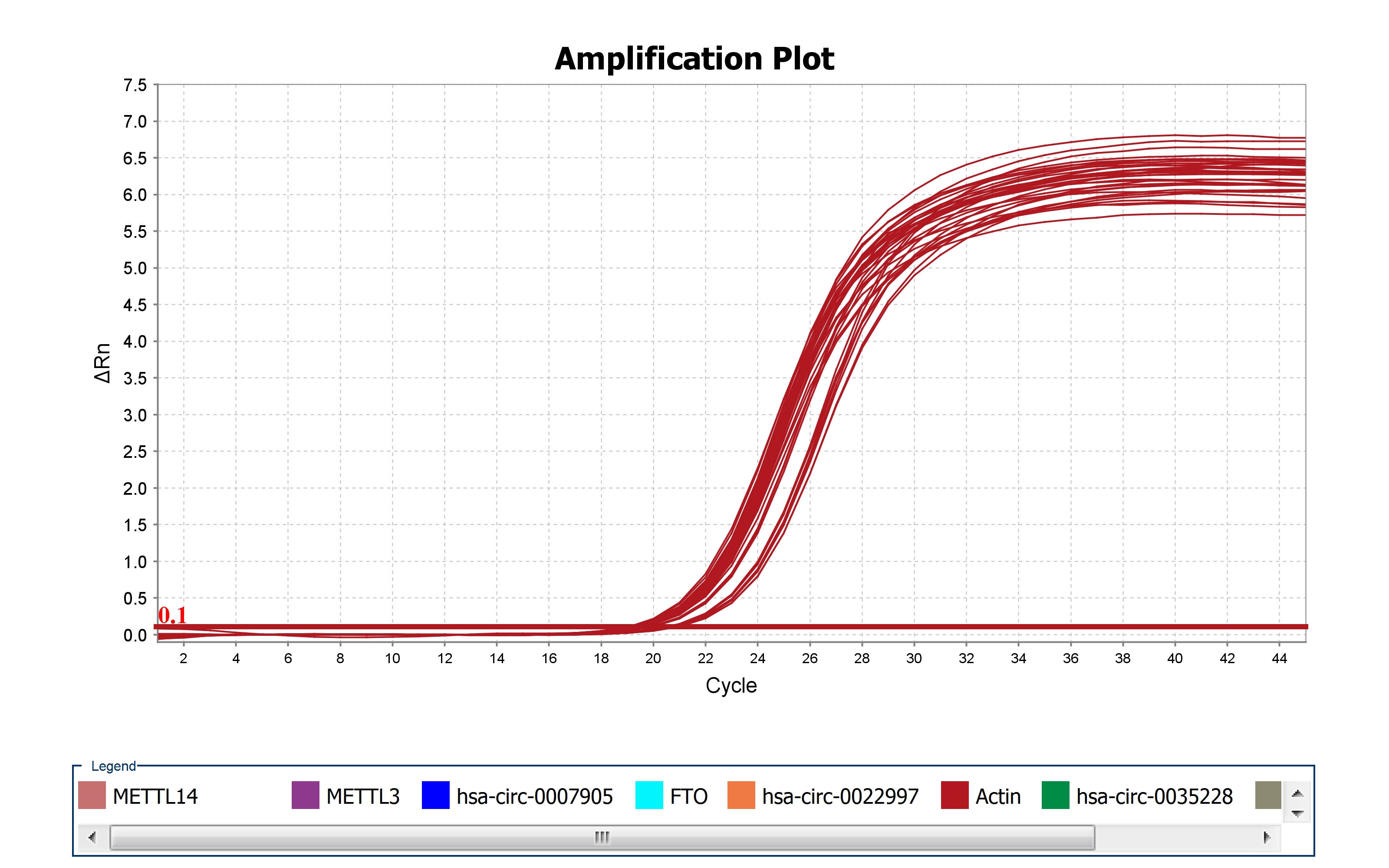

Supplement: Supplemental Information 5 [file peerj-11-14863-s005.zip › Raw data/Fig 1C and 2D/raw data/Amplification Plot Actin (2).jpg]

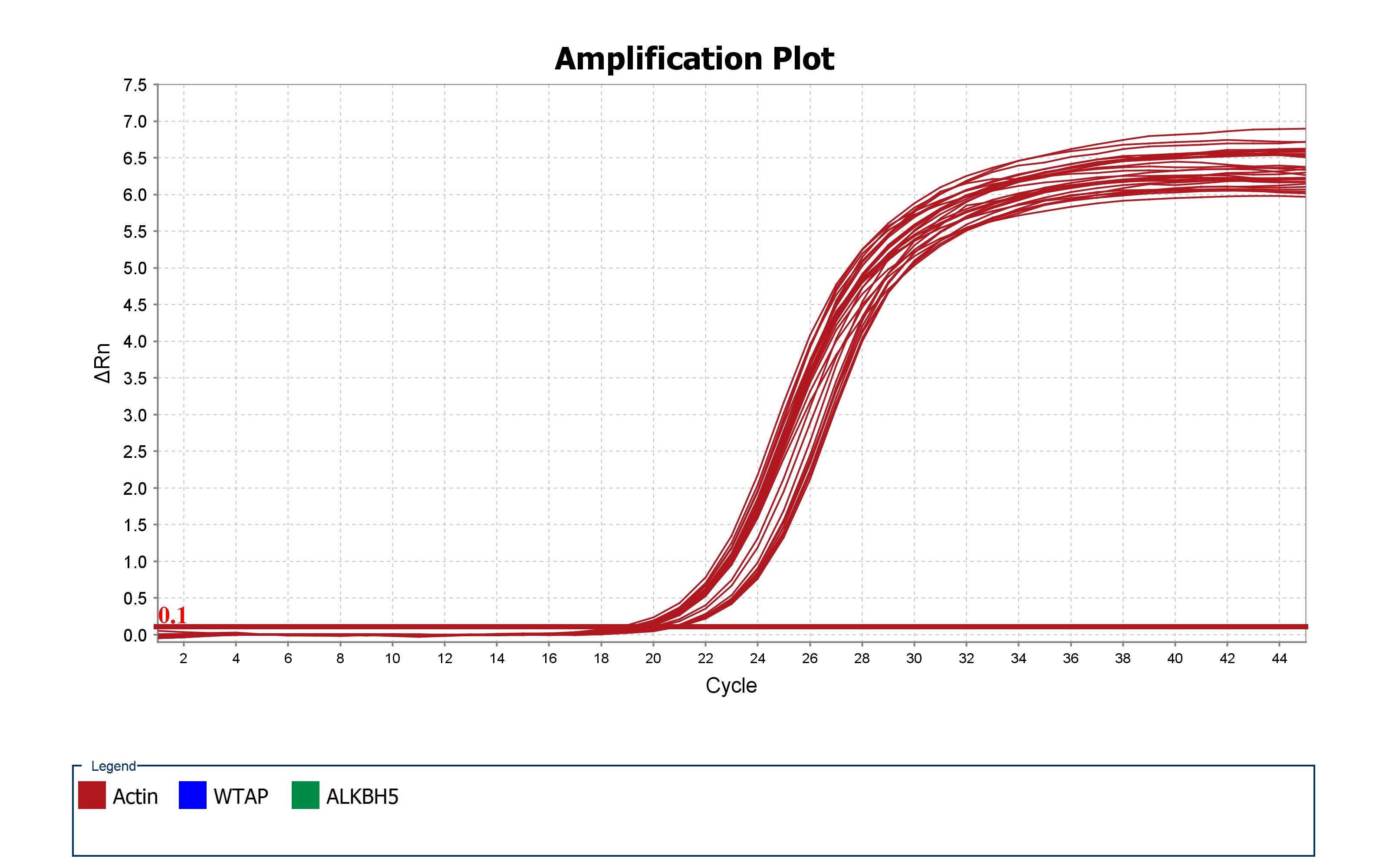

Supplement: Supplemental Information 5 [file peerj-11-14863-s005.zip › Raw data/Fig 1C and 2D/raw data/Amplification Plot Actin.jpg]

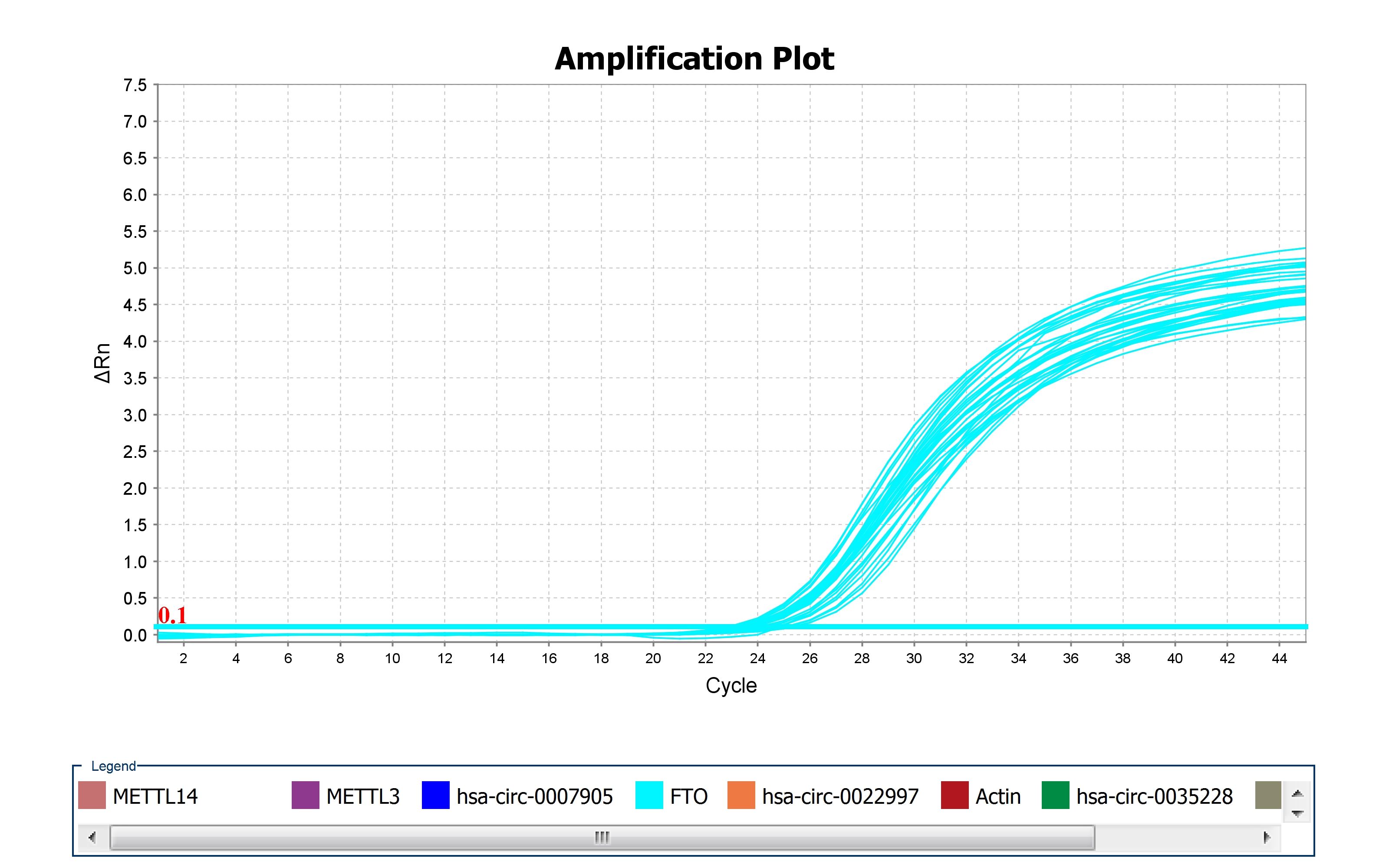

Supplement: Supplemental Information 5 [file peerj-11-14863-s005.zip › Raw data/Fig 1C and 2D/raw data/Amplification Plot FTO.jpg]

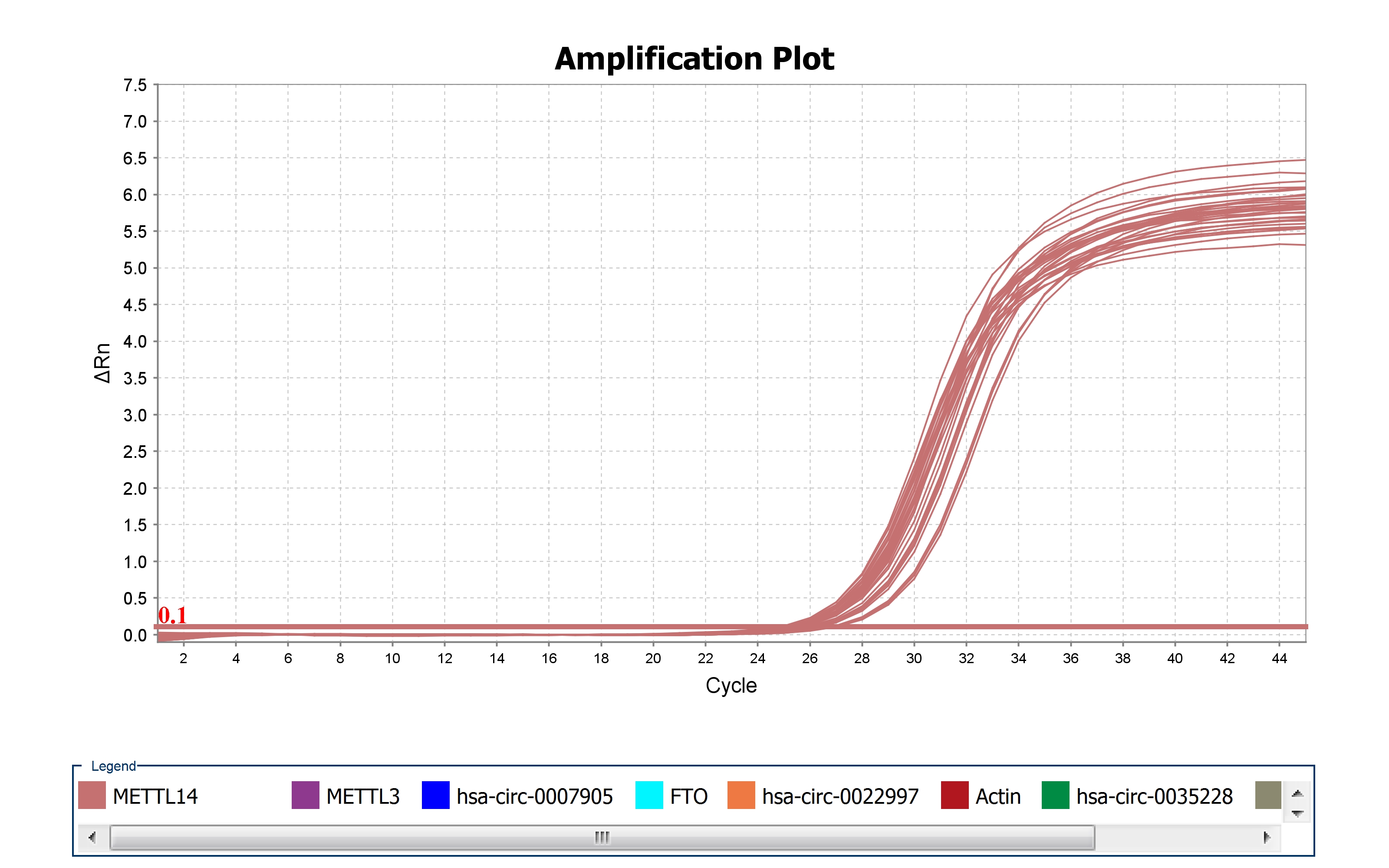

Supplement: Supplemental Information 5 [file peerj-11-14863-s005.zip › Raw data/Fig 1C and 2D/raw data/Amplification Plot METTL14.jpg]

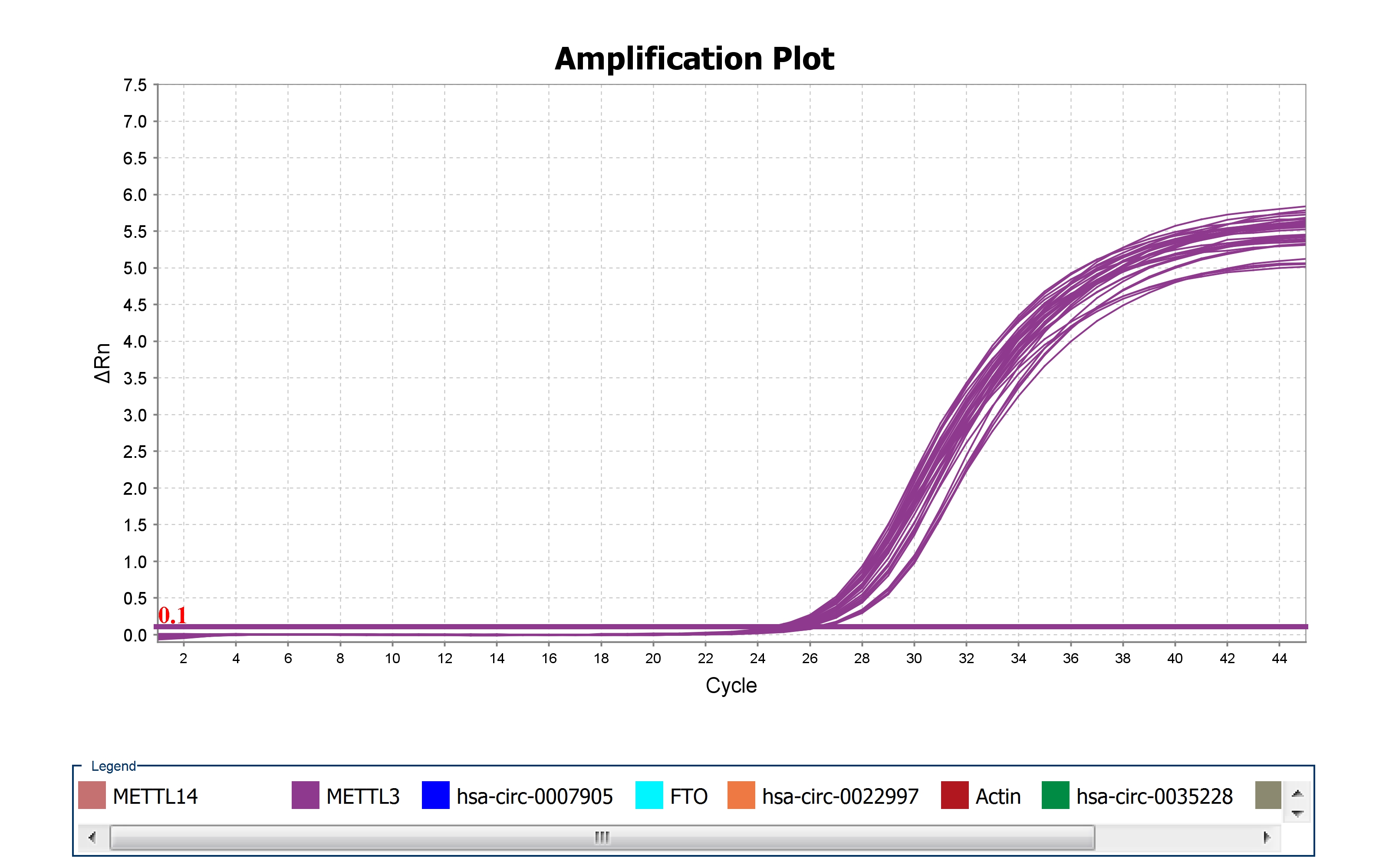

Supplement: Supplemental Information 5 [file peerj-11-14863-s005.zip › Raw data/Fig 1C and 2D/raw data/Amplification Plot METTL3.jpg]

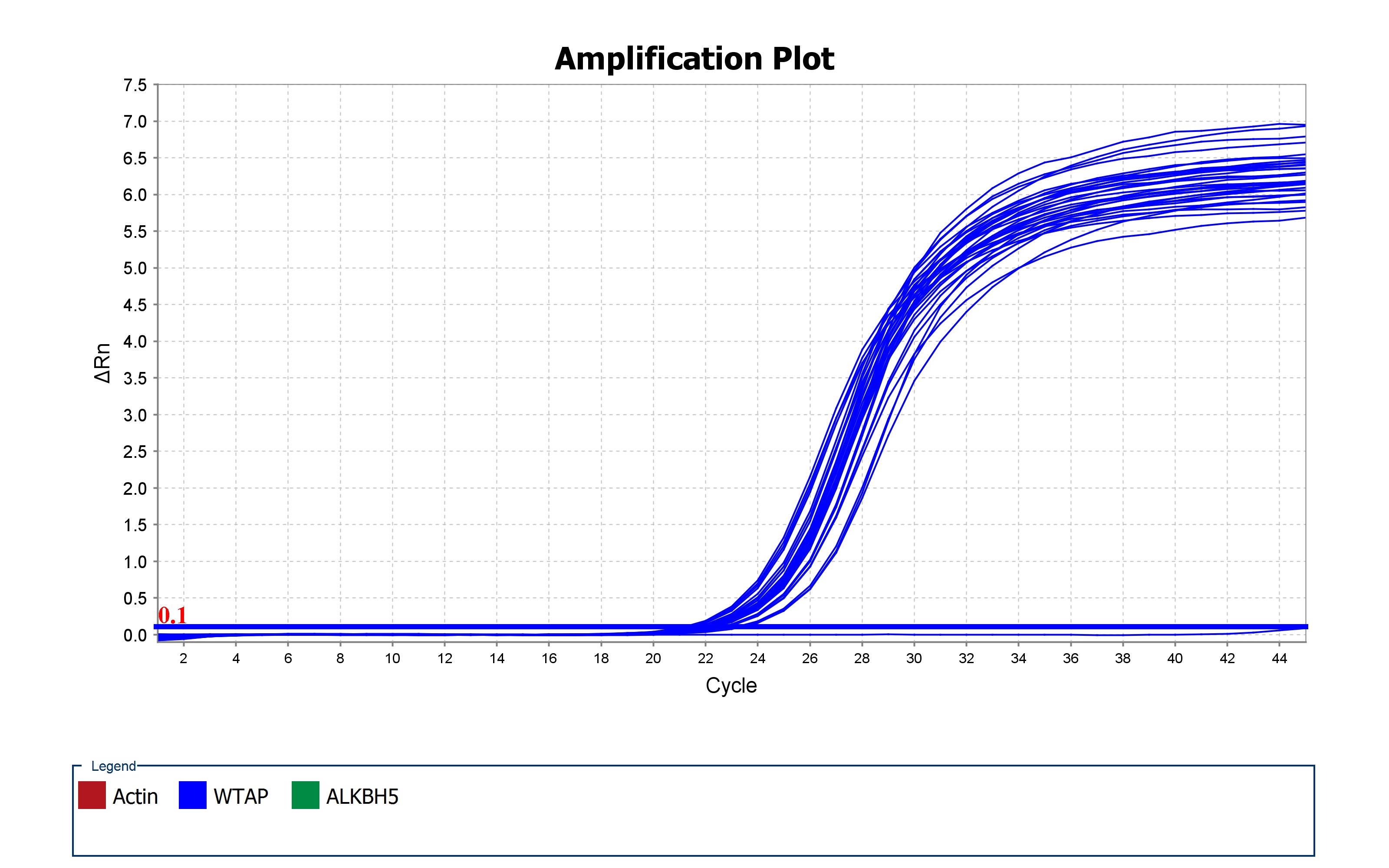

Supplement: Supplemental Information 5 [file peerj-11-14863-s005.zip › Raw data/Fig 1C and 2D/raw data/Amplification Plot WTAP.jpg]

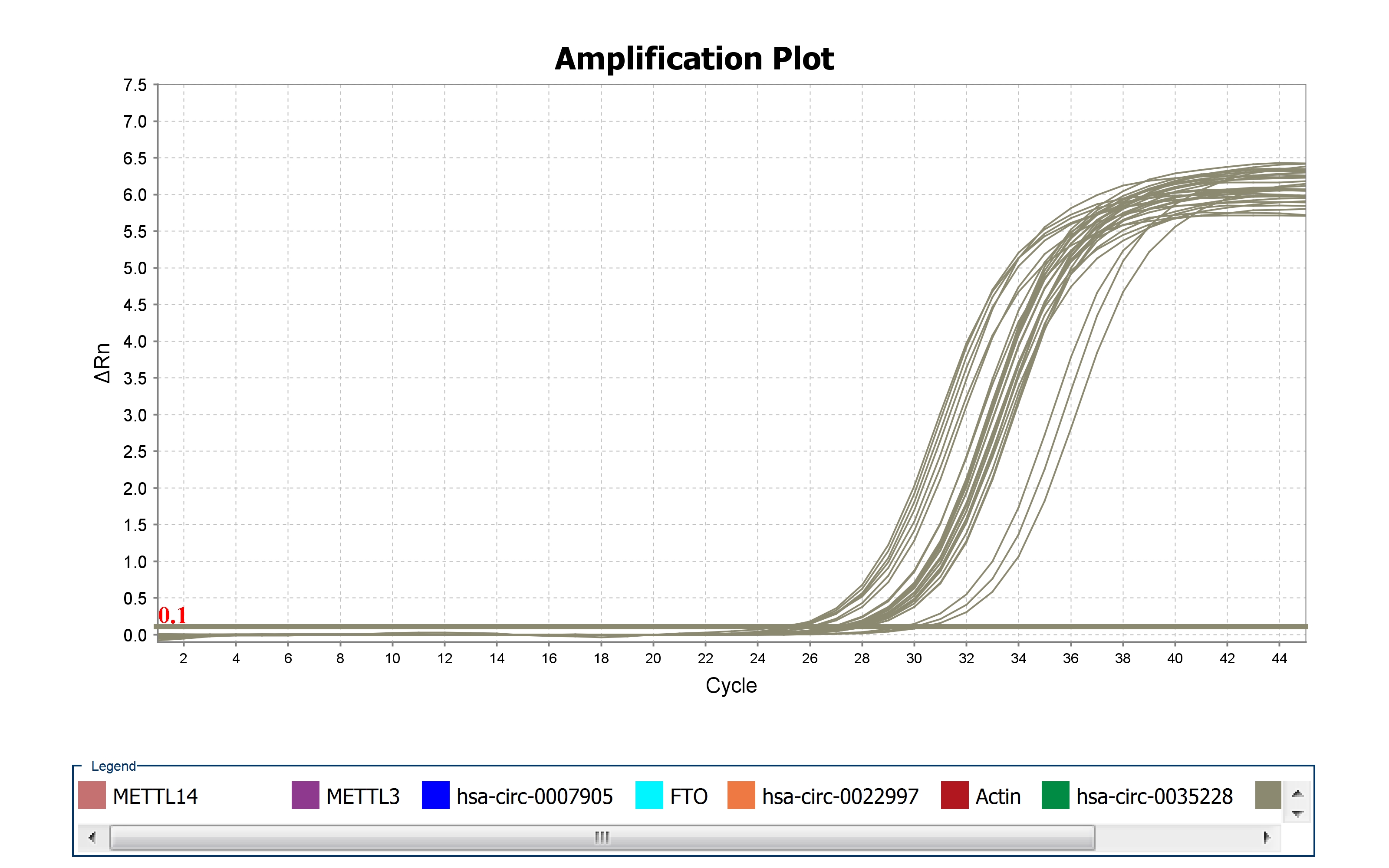

Supplement: Supplemental Information 5 [file peerj-11-14863-s005.zip › Raw data/Fig 1C and 2D/raw data/Amplification Plot hsa-circ-0003949.jpg]

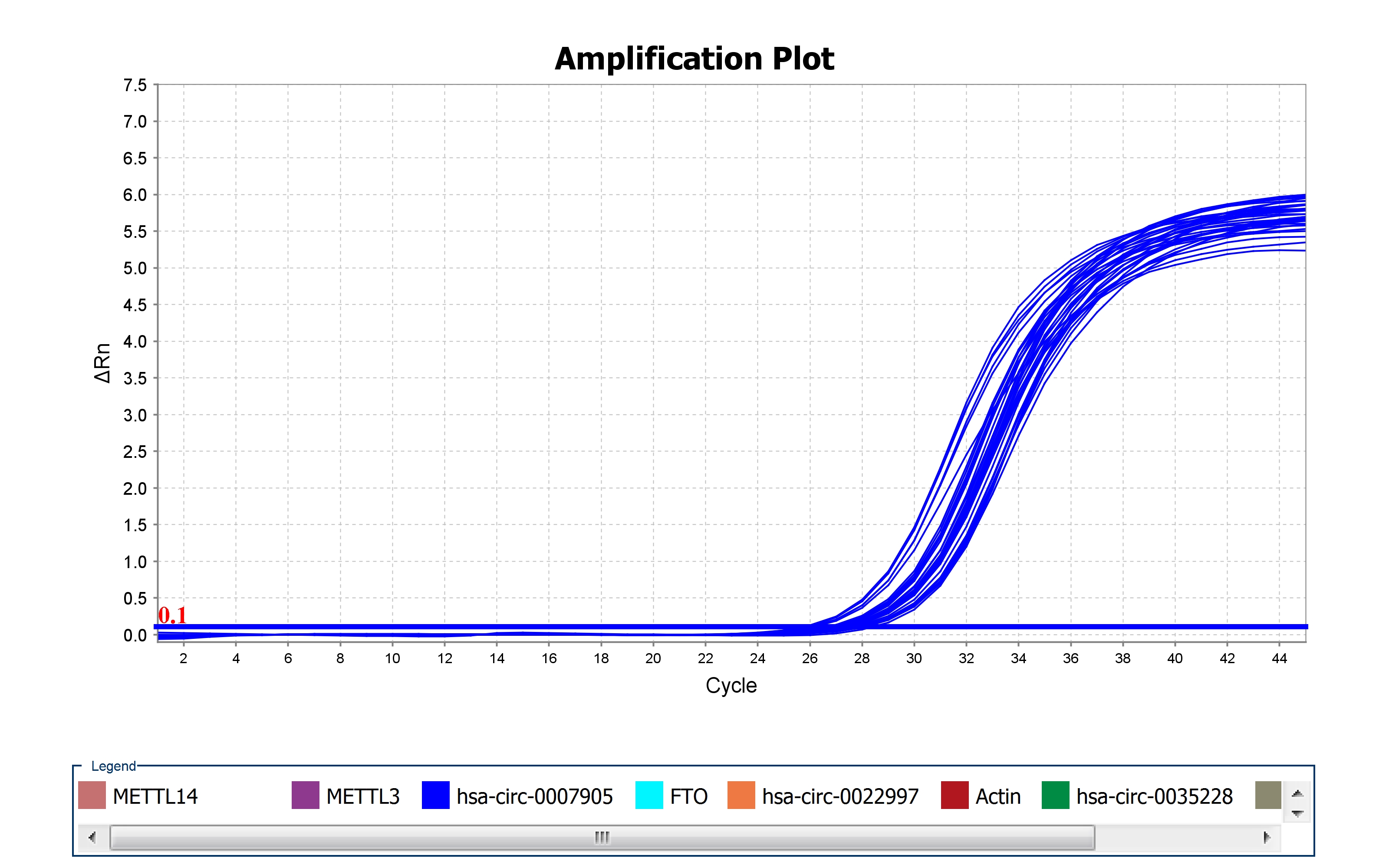

Supplement: Supplemental Information 5 [file peerj-11-14863-s005.zip › Raw data/Fig 1C and 2D/raw data/Amplification Plot hsa-circ-0007905.jpg]

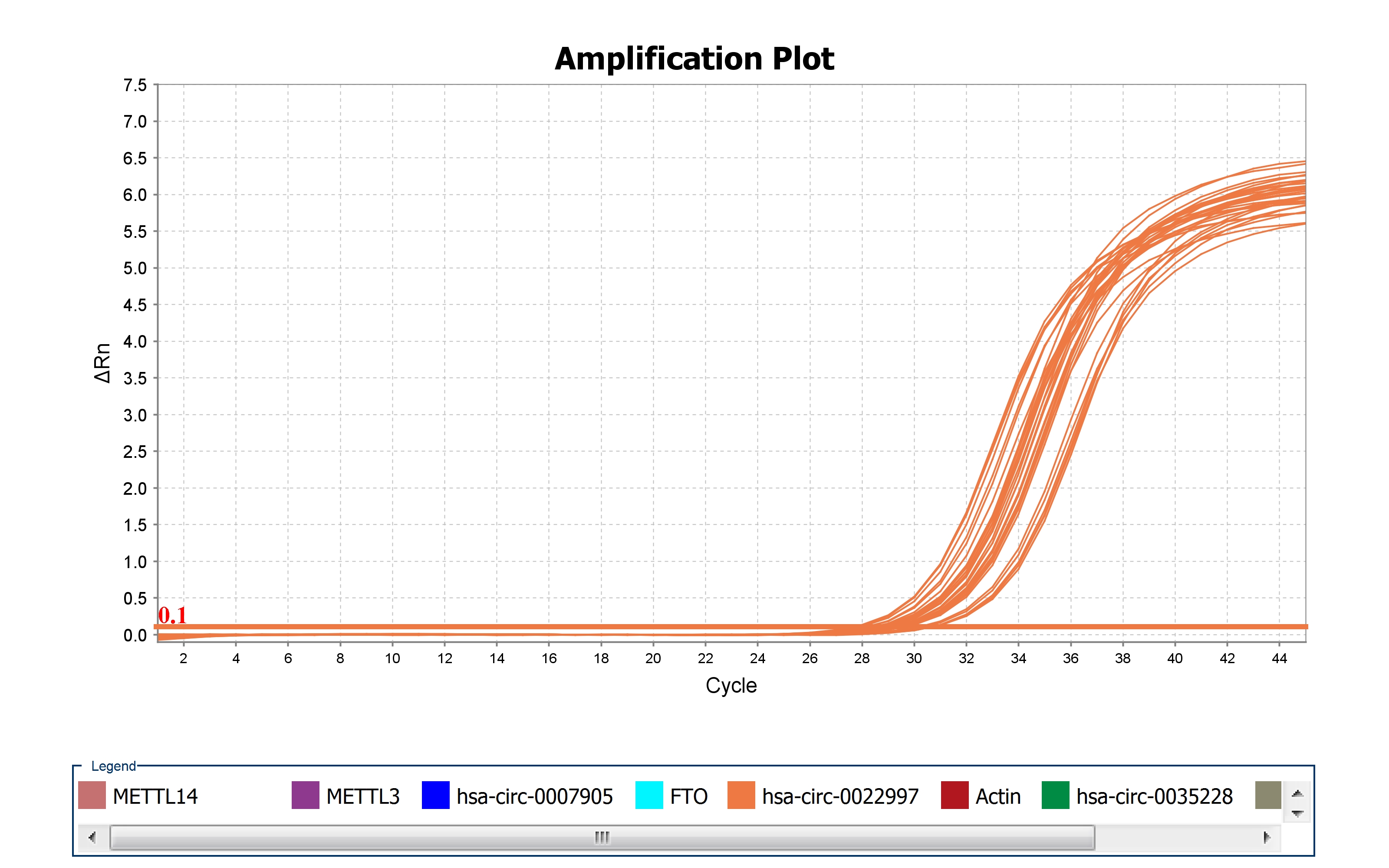

Supplement: Supplemental Information 5 [file peerj-11-14863-s005.zip › Raw data/Fig 1C and 2D/raw data/Amplification Plot hsa-circ-0022997.jpg]

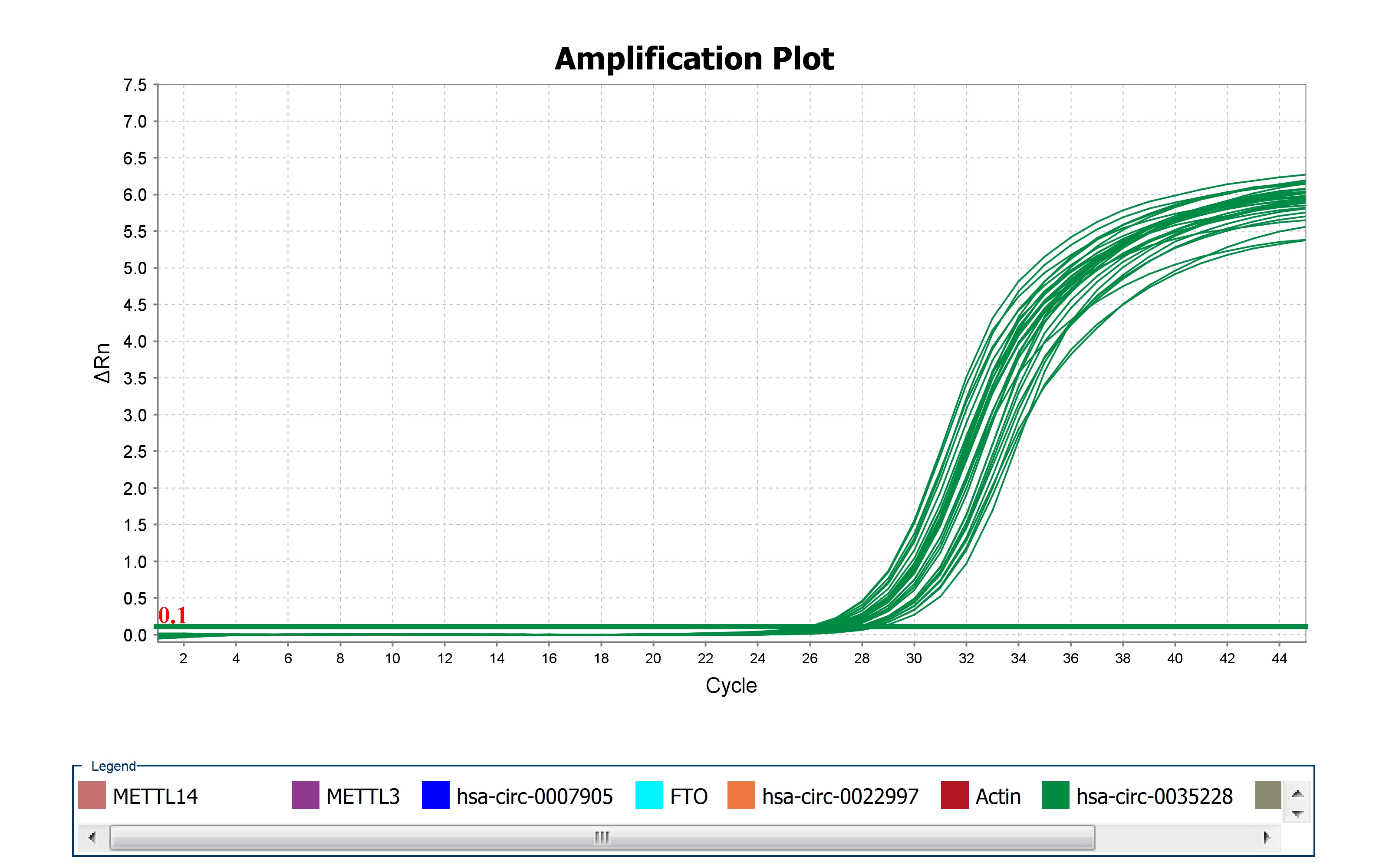

Supplement: Supplemental Information 5 [file peerj-11-14863-s005.zip › Raw data/Fig 1C and 2D/raw data/Amplification Plot hsa-circ-0035228.jpg]

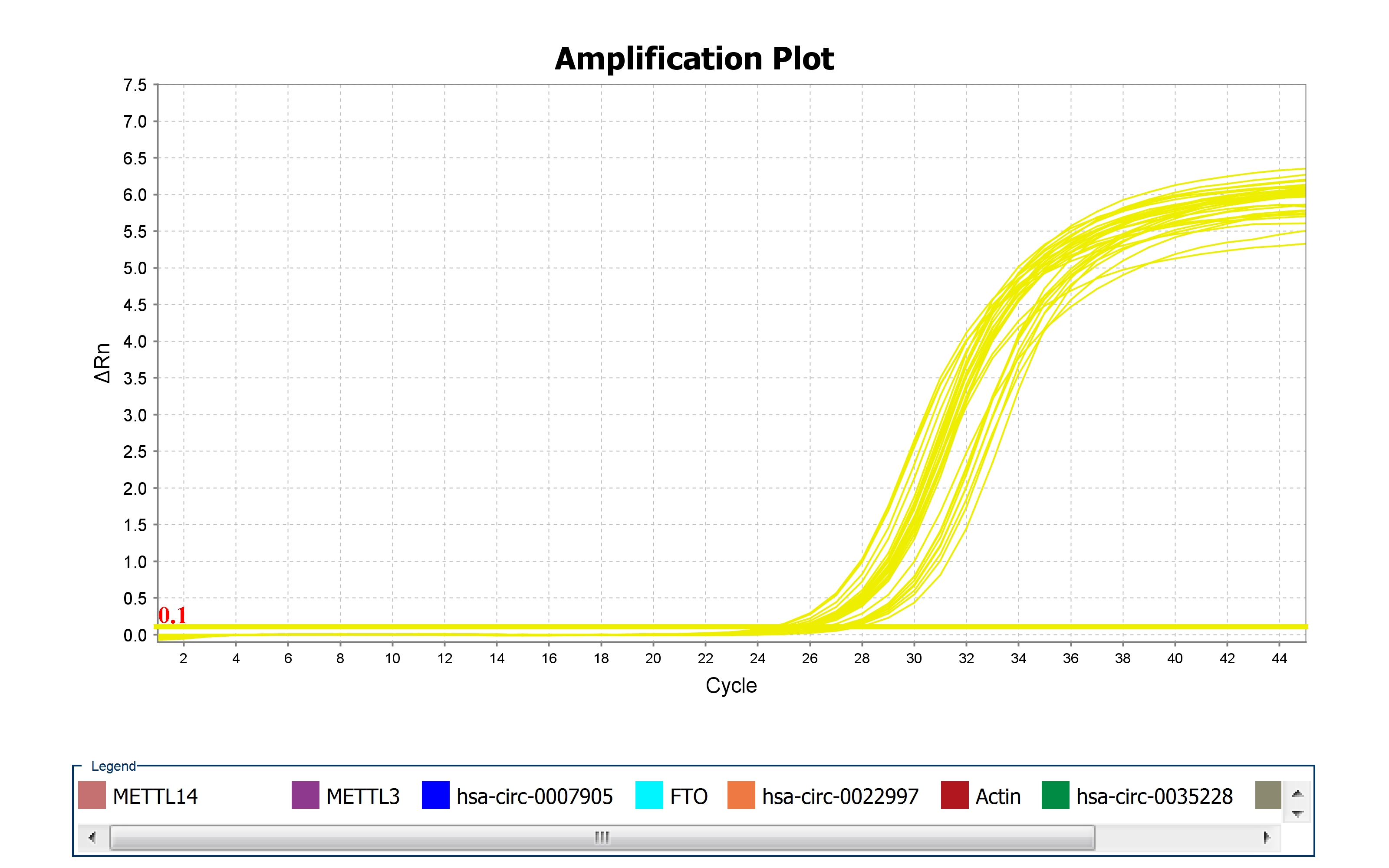

Supplement: Supplemental Information 5 [file peerj-11-14863-s005.zip › Raw data/Fig 1C and 2D/raw data/Amplification Plot hsa-circ-0065244.jpg]

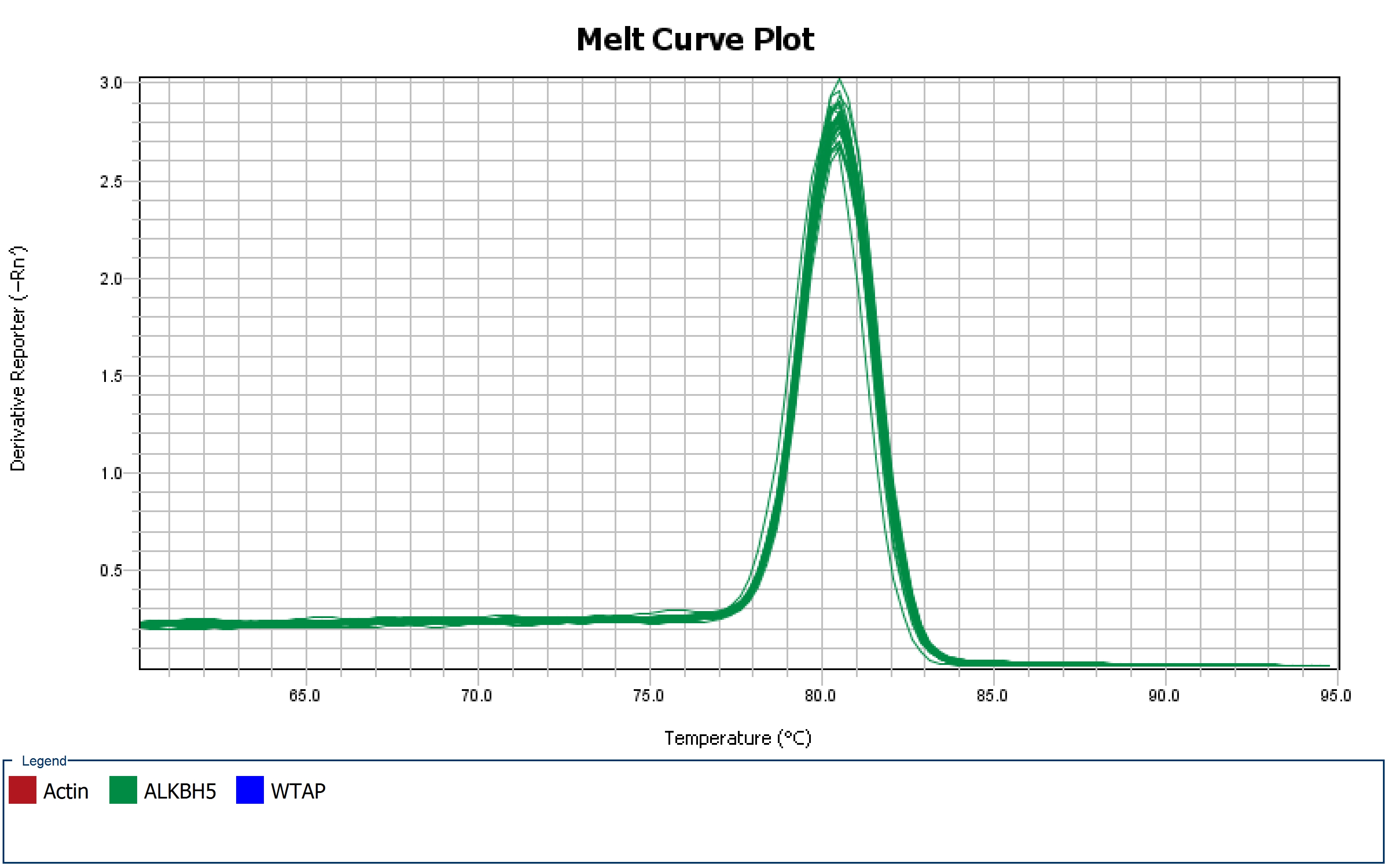

Supplement: Supplemental Information 5 [file peerj-11-14863-s005.zip › Raw data/Fig 1C and 2D/raw data/Melt Curve Plot ALKBH5.jpg]

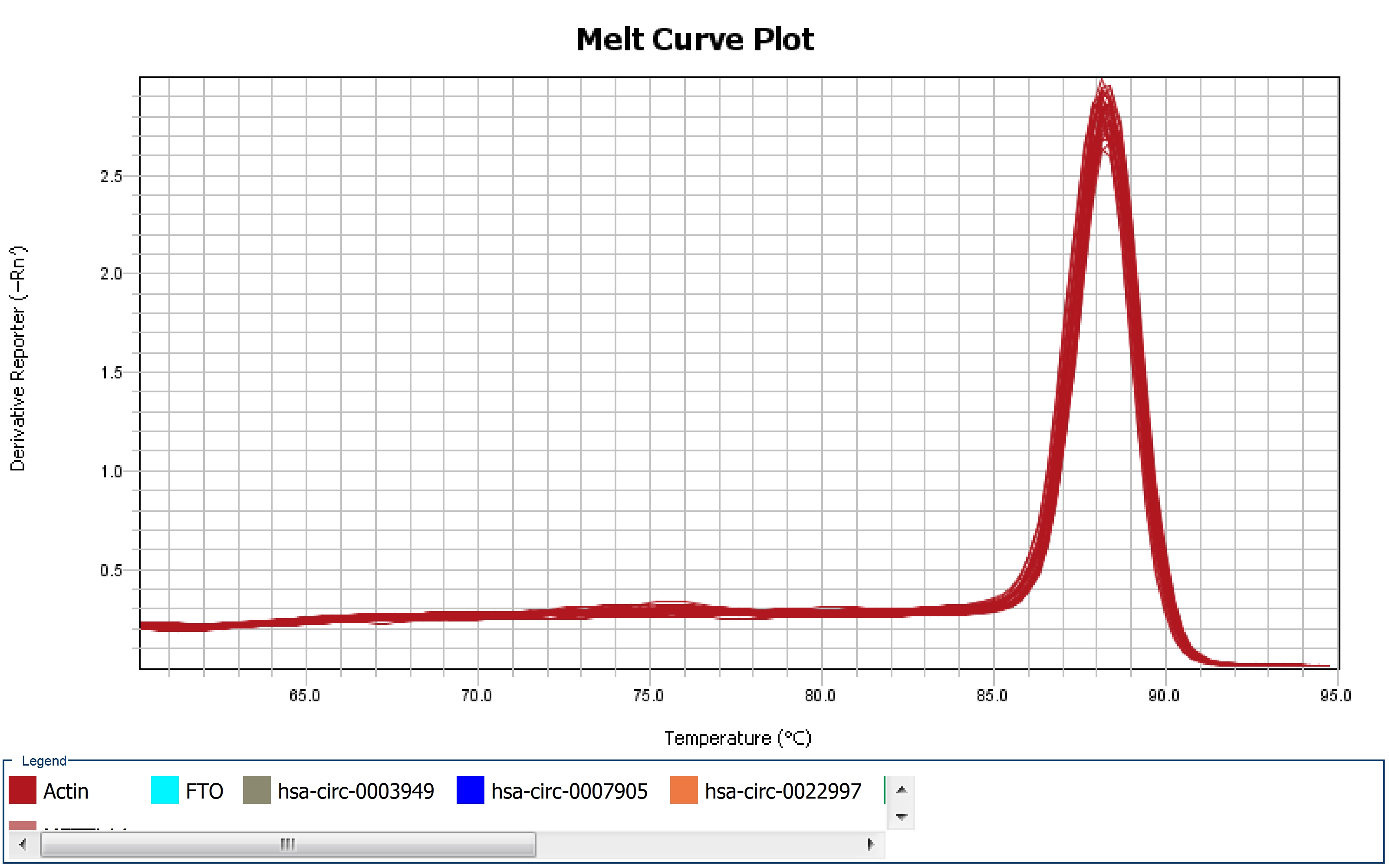

Supplement: Supplemental Information 5 [file peerj-11-14863-s005.zip › Raw data/Fig 1C and 2D/raw data/Melt Curve Plot Actin (2).jpg]

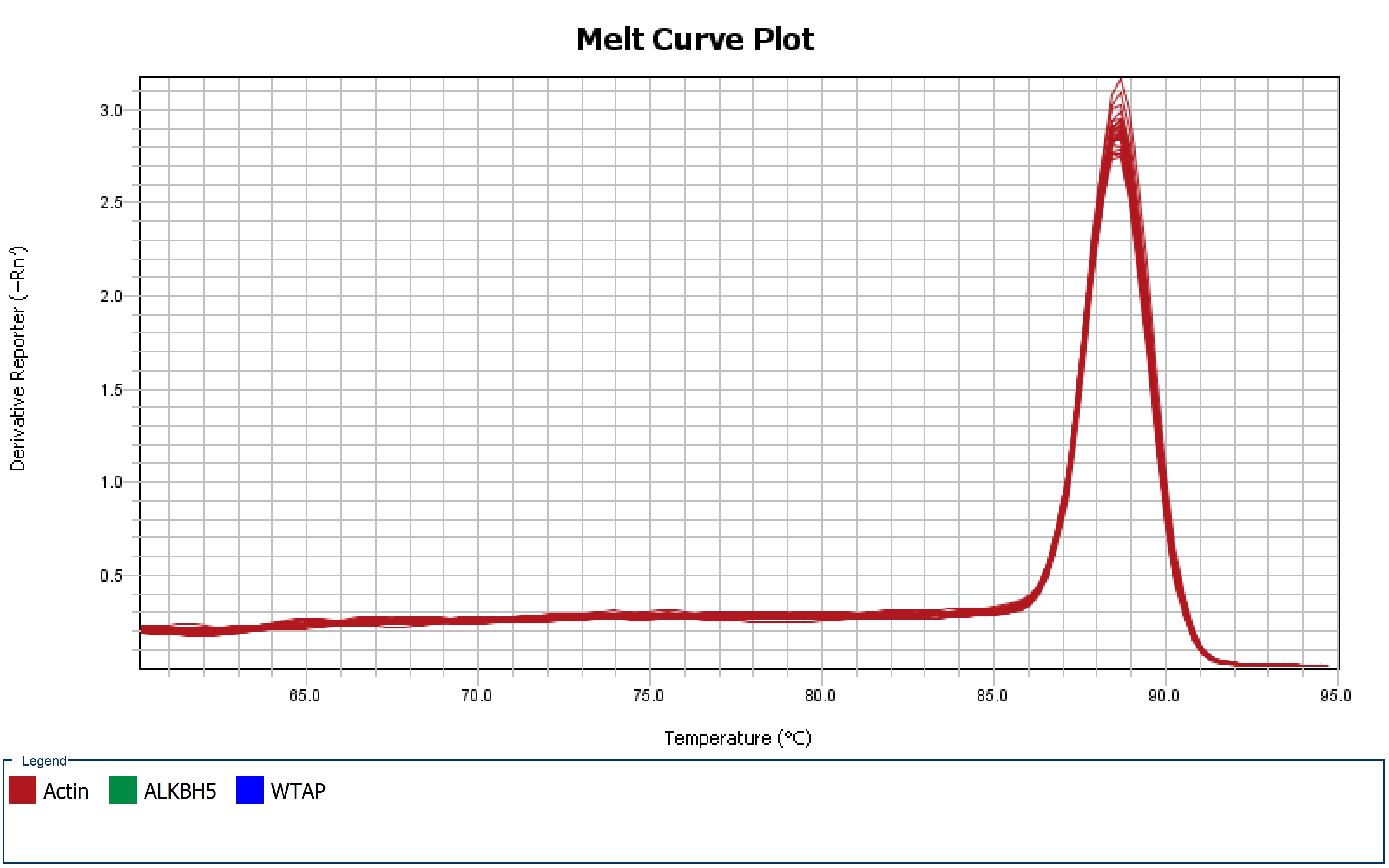

Supplement: Supplemental Information 5 [file peerj-11-14863-s005.zip › Raw data/Fig 1C and 2D/raw data/Melt Curve Plot Actin.jpg]

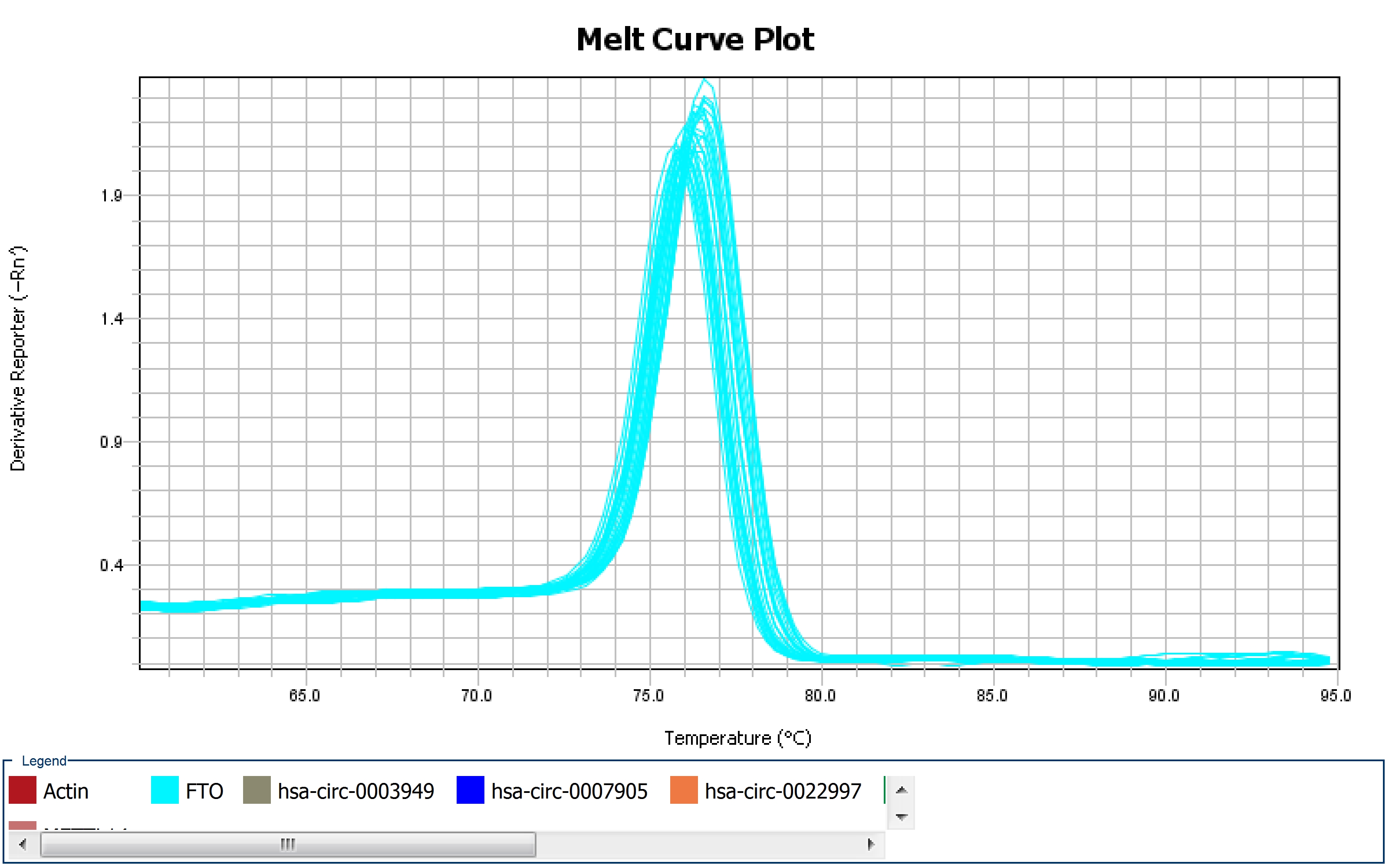

Supplement: Supplemental Information 5 [file peerj-11-14863-s005.zip › Raw data/Fig 1C and 2D/raw data/Melt Curve Plot FTO.jpg]

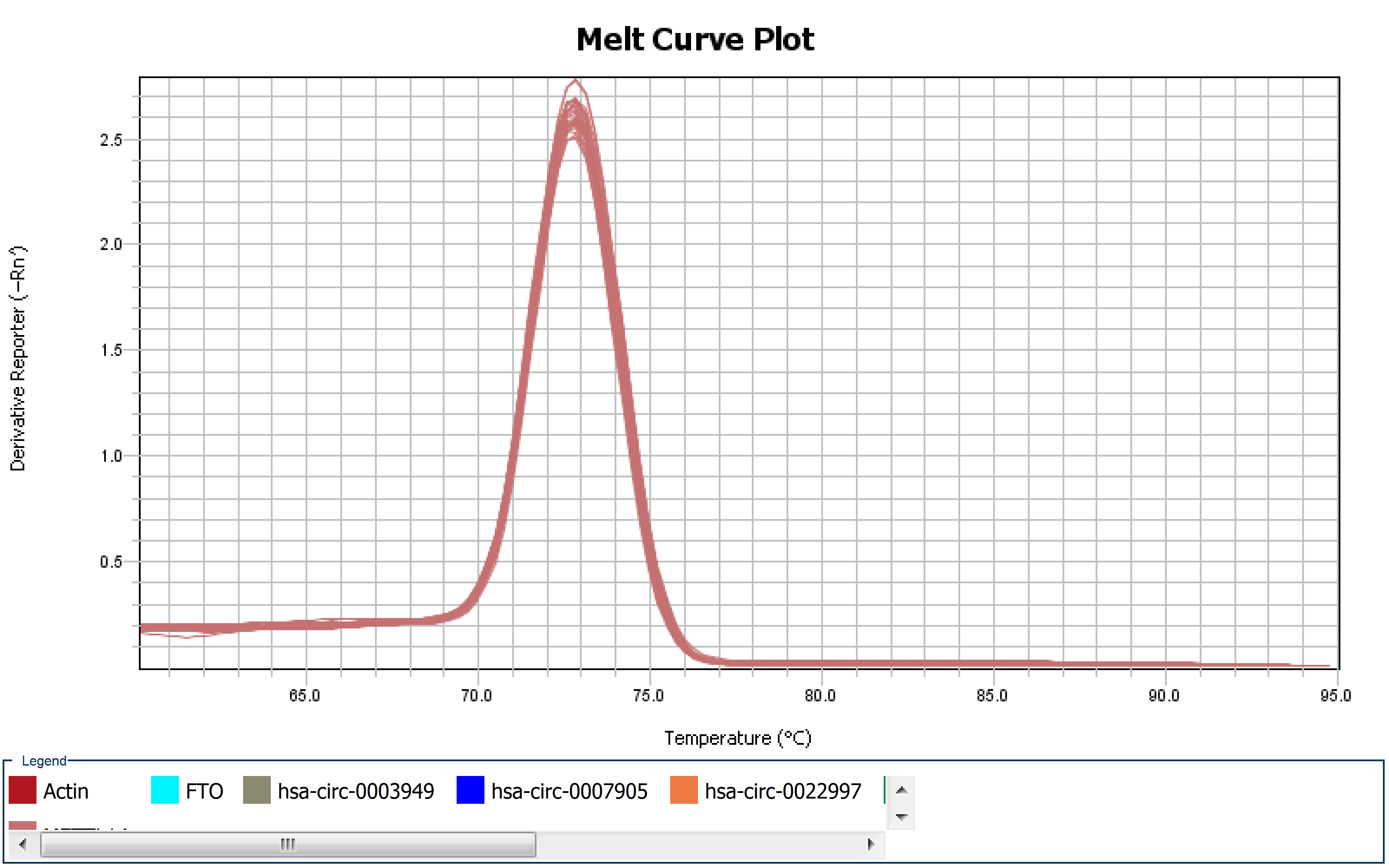

Supplement: Supplemental Information 5 [file peerj-11-14863-s005.zip › Raw data/Fig 1C and 2D/raw data/Melt Curve Plot METTL14.jpg]

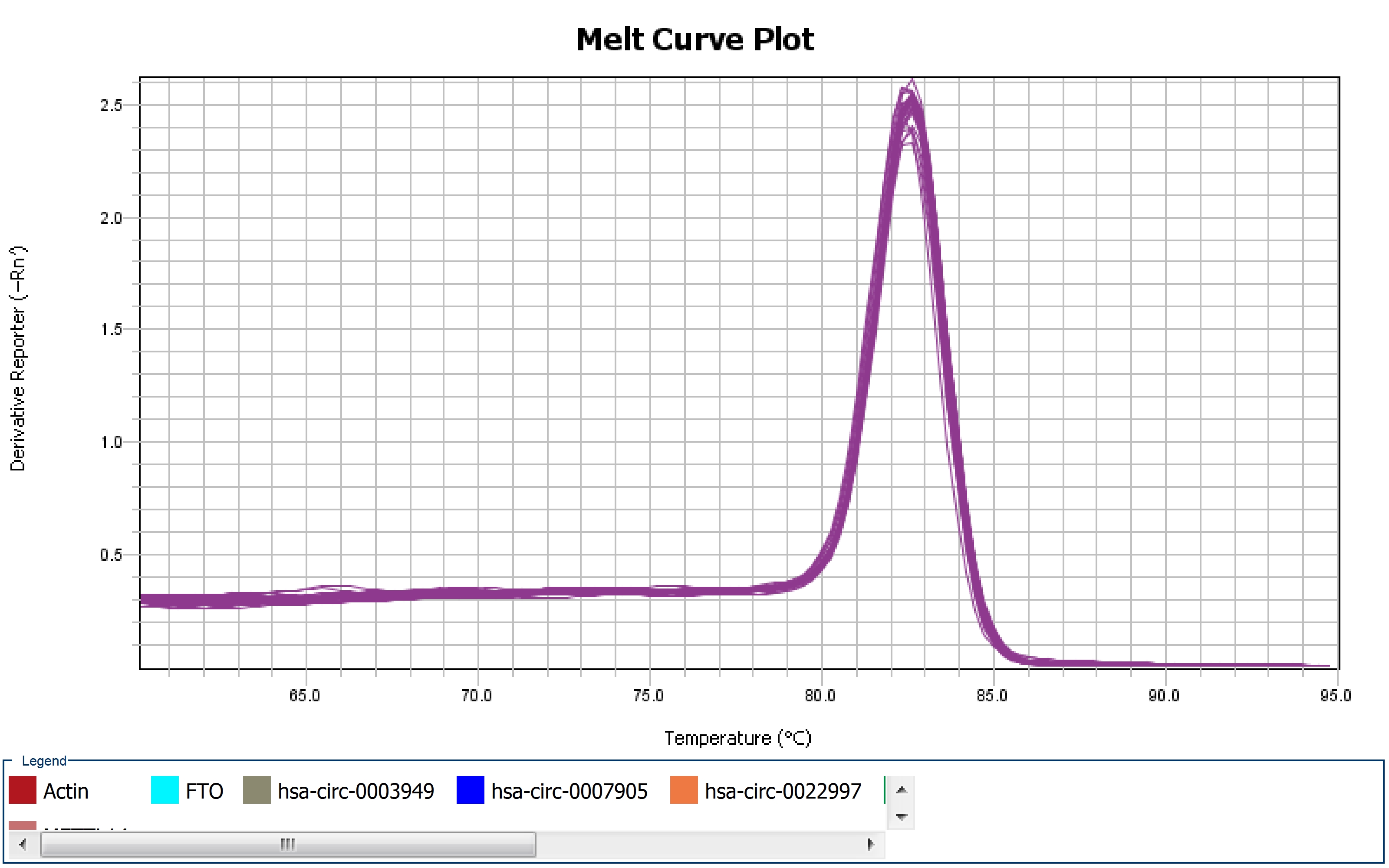

Supplement: Supplemental Information 5 [file peerj-11-14863-s005.zip › Raw data/Fig 1C and 2D/raw data/Melt Curve Plot METTL3.jpg]

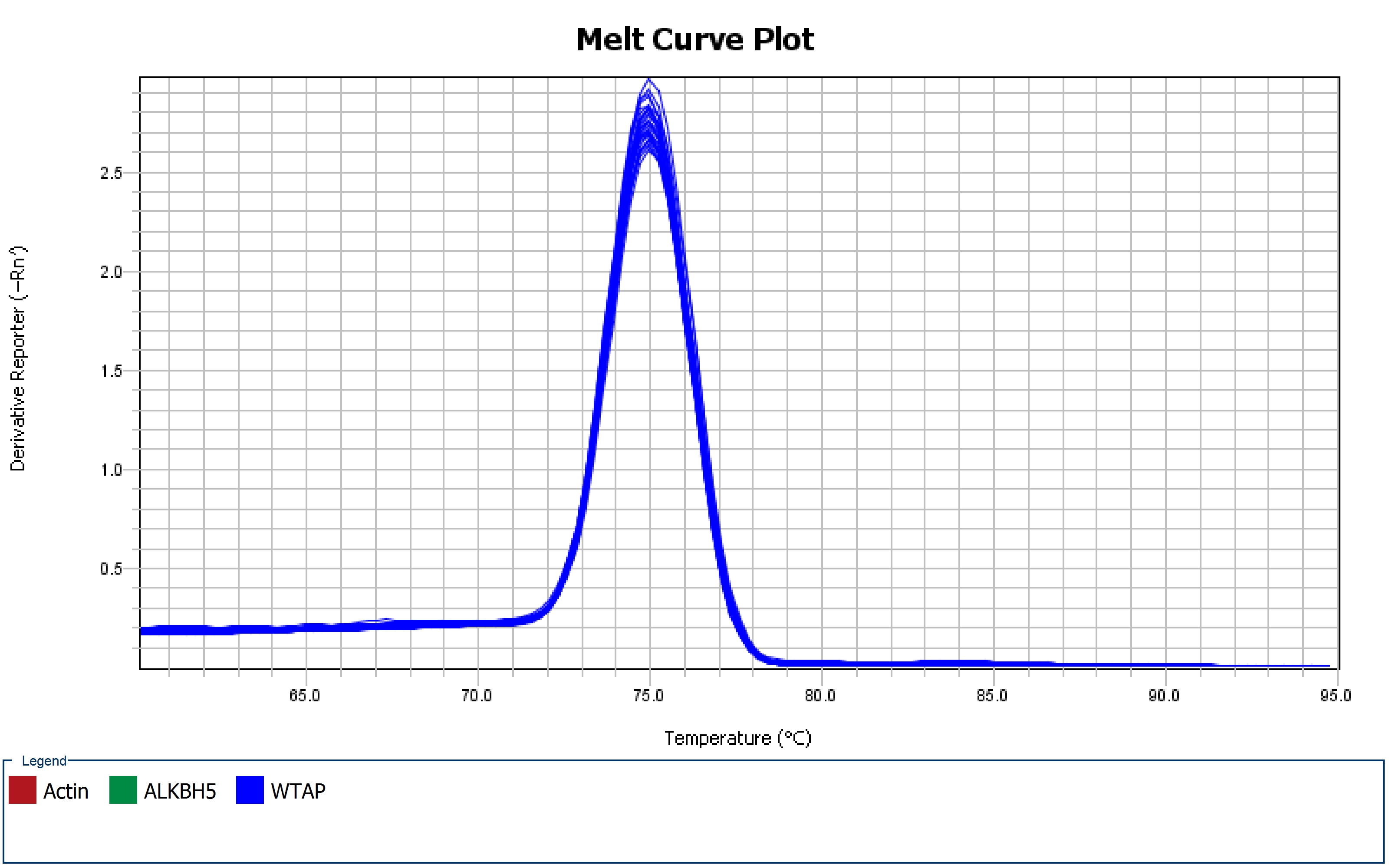

Supplement: Supplemental Information 5 [file peerj-11-14863-s005.zip › Raw data/Fig 1C and 2D/raw data/Melt Curve Plot WTAP.jpg]

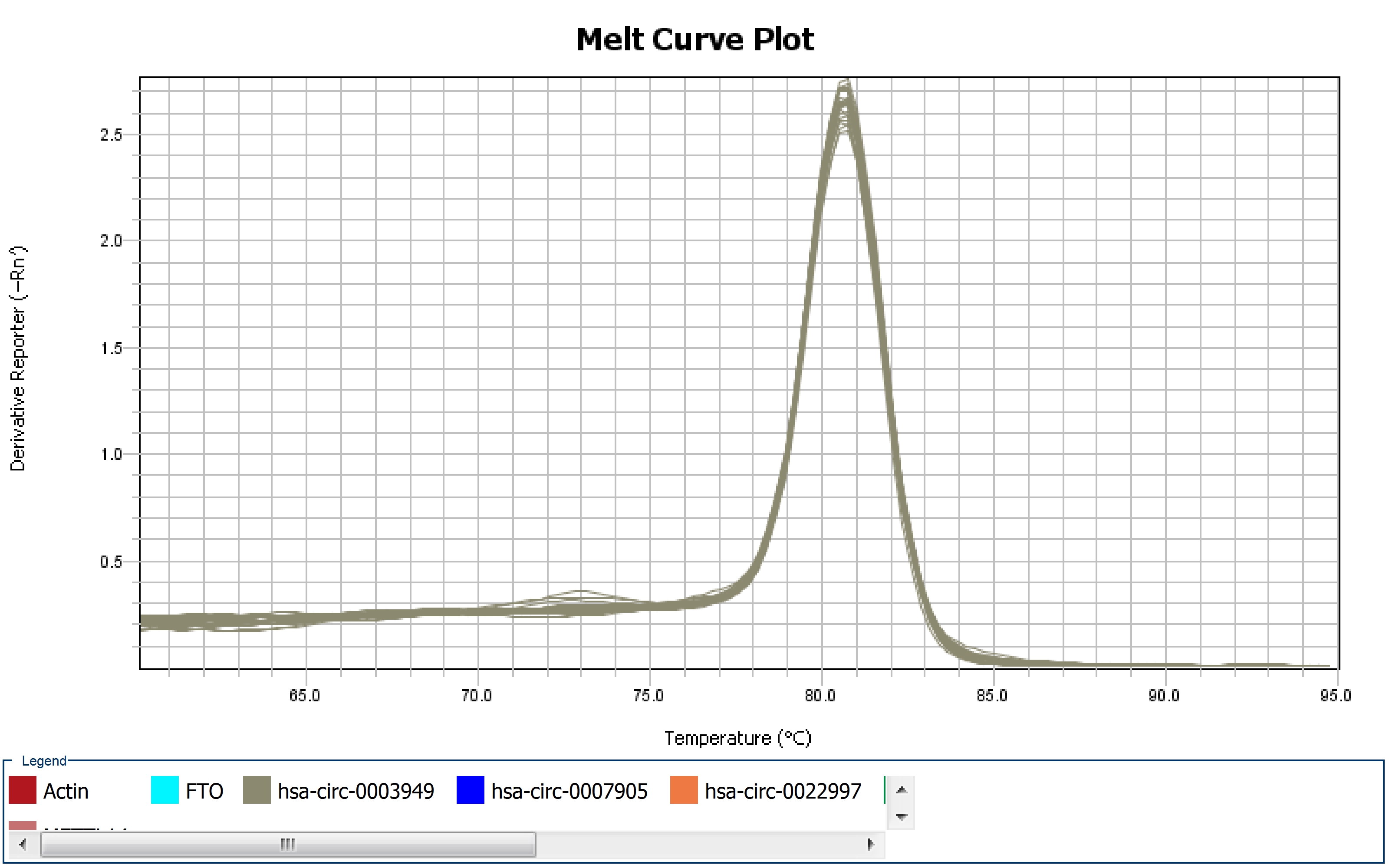

Supplement: Supplemental Information 5 [file peerj-11-14863-s005.zip › Raw data/Fig 1C and 2D/raw data/Melt Curve Plot hsa-circ-0003949.jpg]

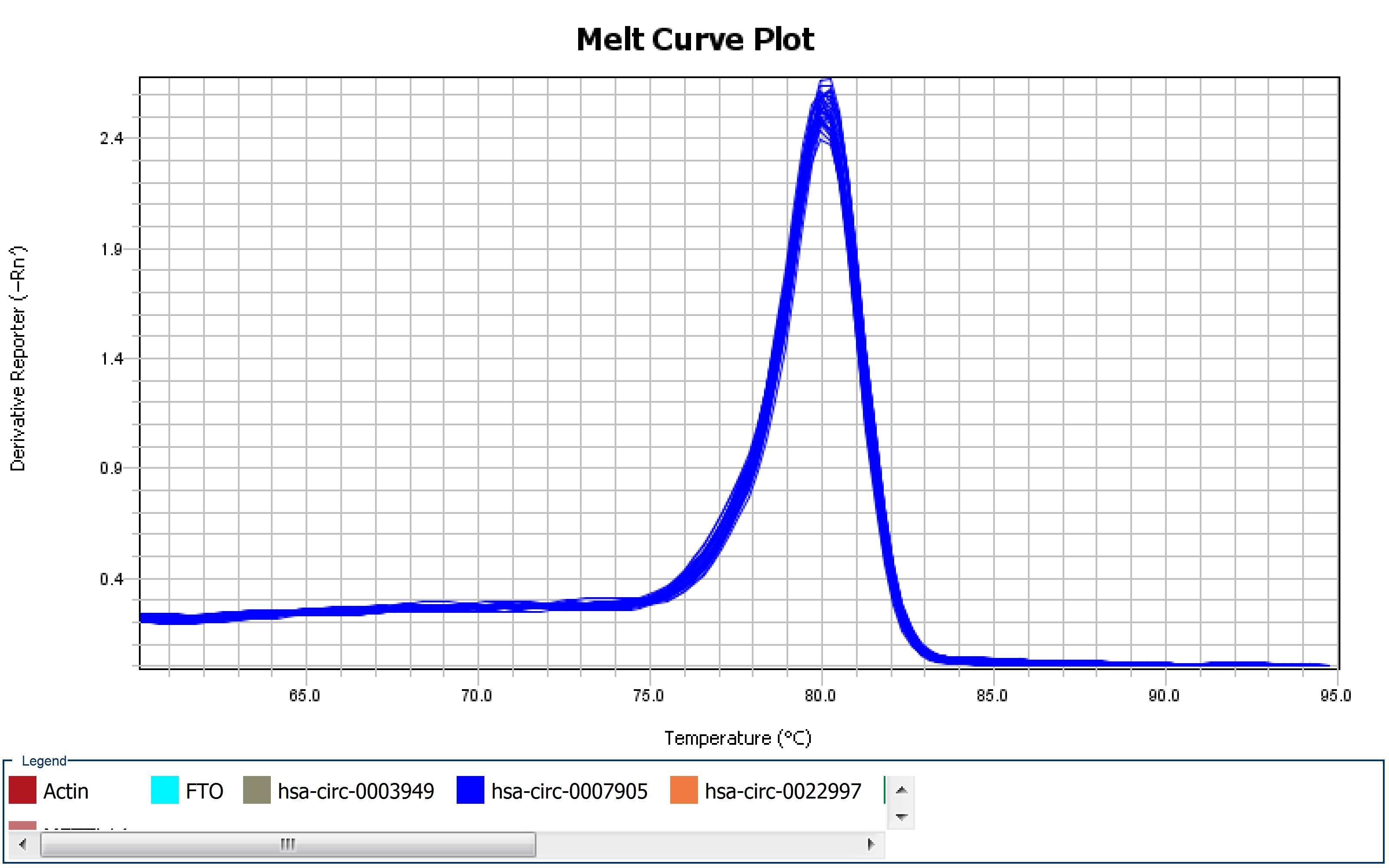

Supplement: Supplemental Information 5 [file peerj-11-14863-s005.zip › Raw data/Fig 1C and 2D/raw data/Melt Curve Plot hsa-circ-0007905.jpg]

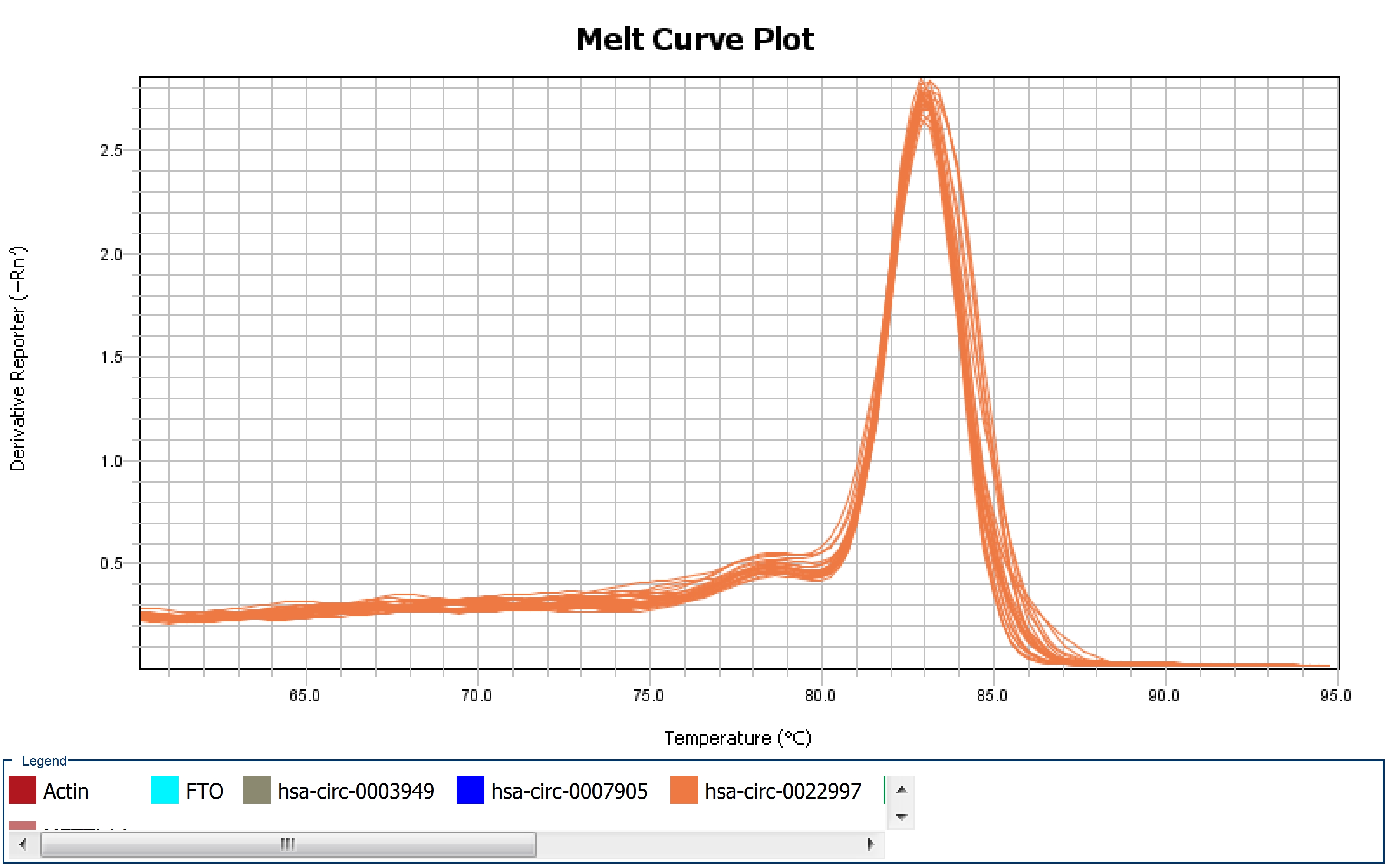

Supplement: Supplemental Information 5 [file peerj-11-14863-s005.zip › Raw data/Fig 1C and 2D/raw data/Melt Curve Plot hsa-circ-0022997.jpg]

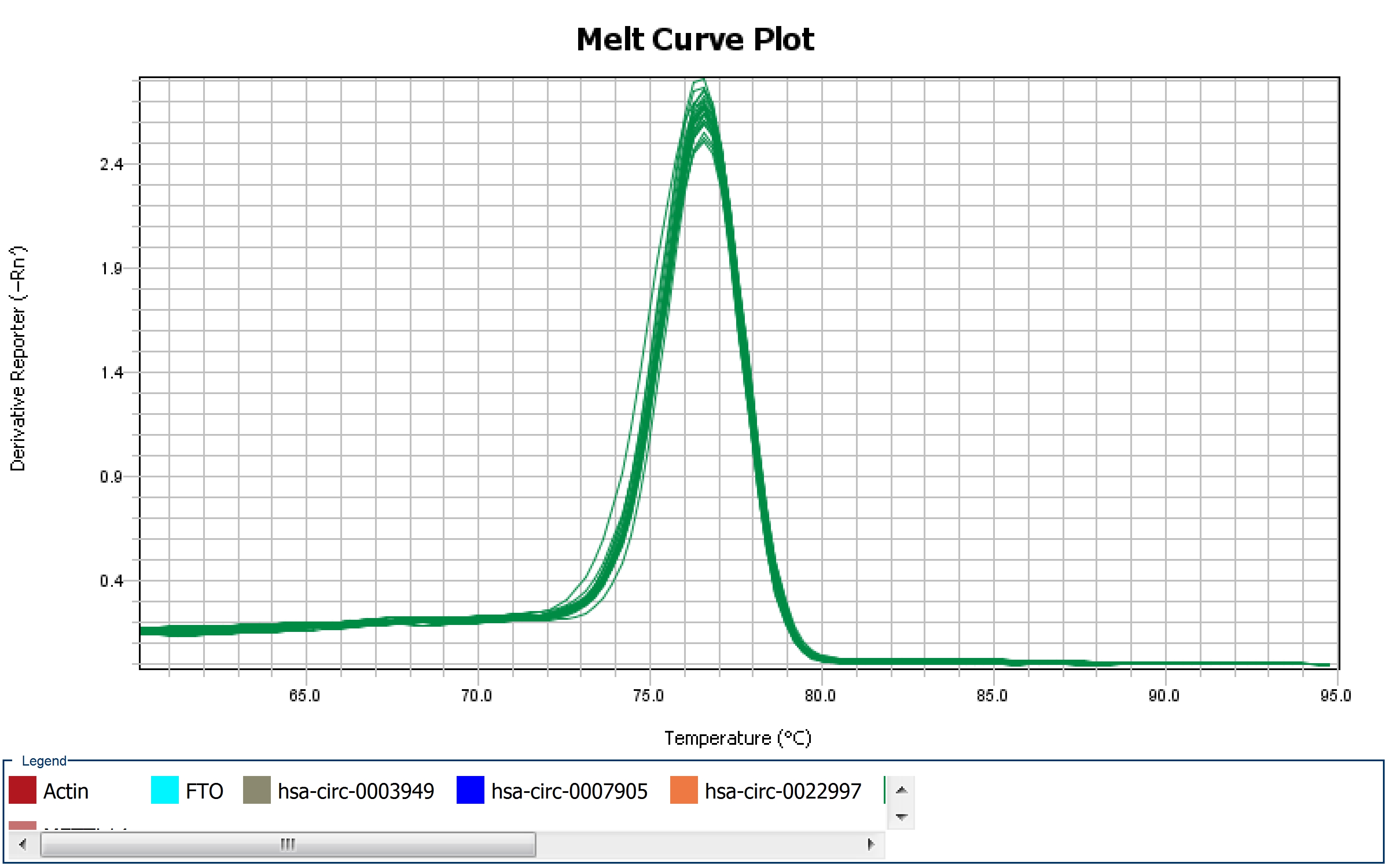

Supplement: Supplemental Information 5 [file peerj-11-14863-s005.zip › Raw data/Fig 1C and 2D/raw data/Melt Curve Plot hsa-circ-0035228.jpg]

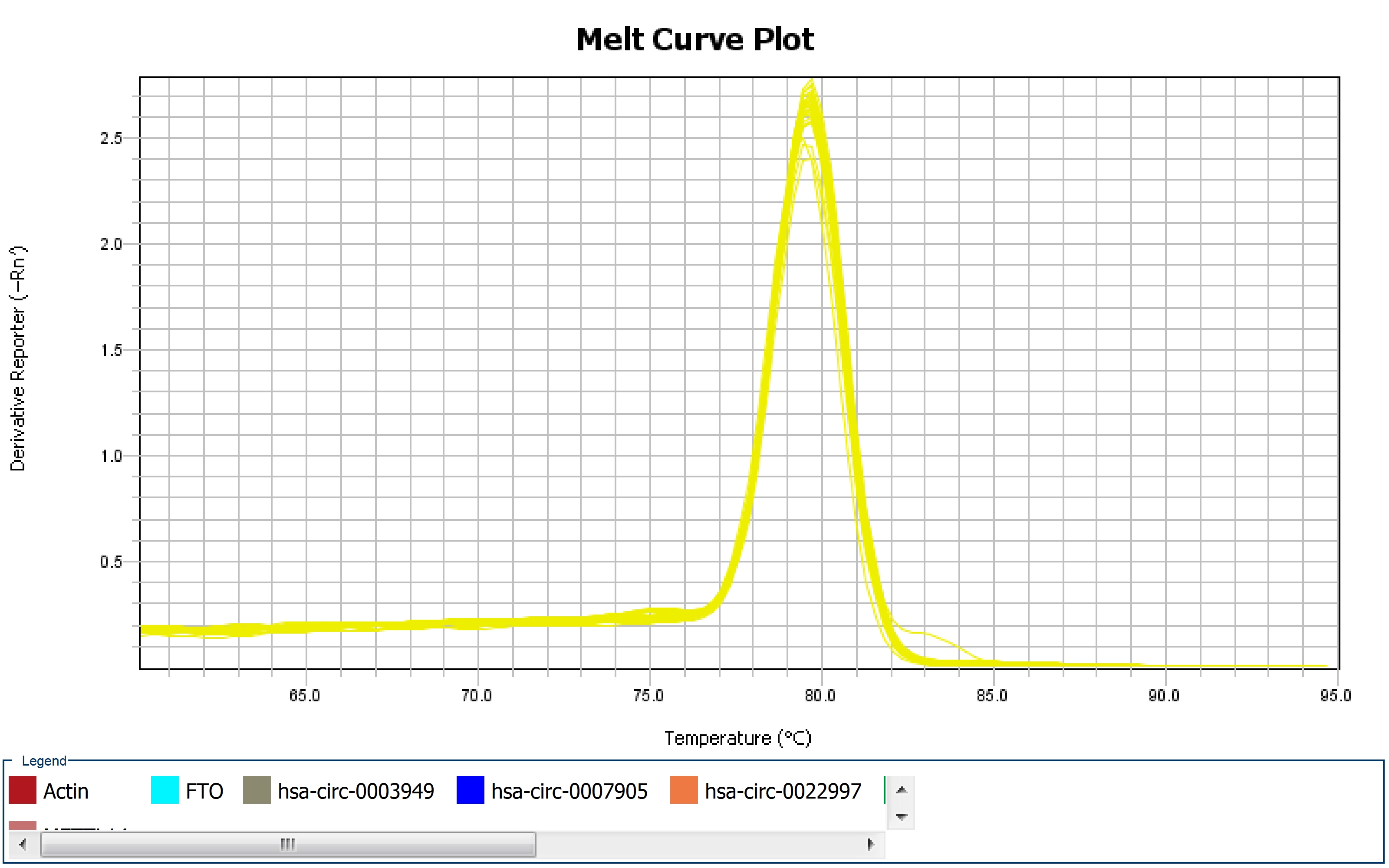

Supplement: Supplemental Information 5 [file peerj-11-14863-s005.zip › Raw data/Fig 1C and 2D/raw data/Melt Curve Plot hsa-circ-0065244.jpg]

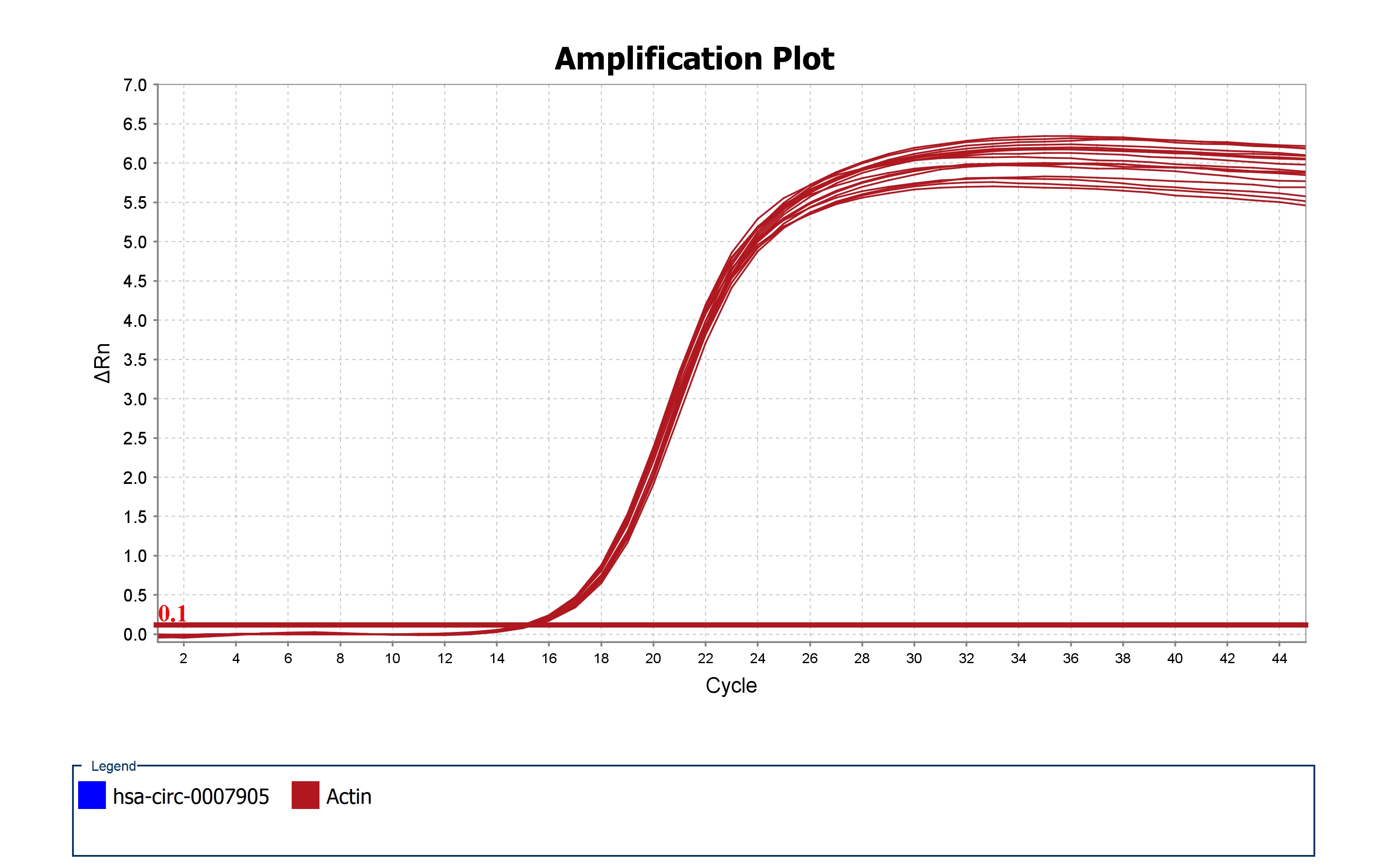

Supplement: Supplemental Information 5 [file peerj-11-14863-s005.zip › Raw data/Fig 1D and 2G/Raw data/Amplification Plot Actin (2).jpg]

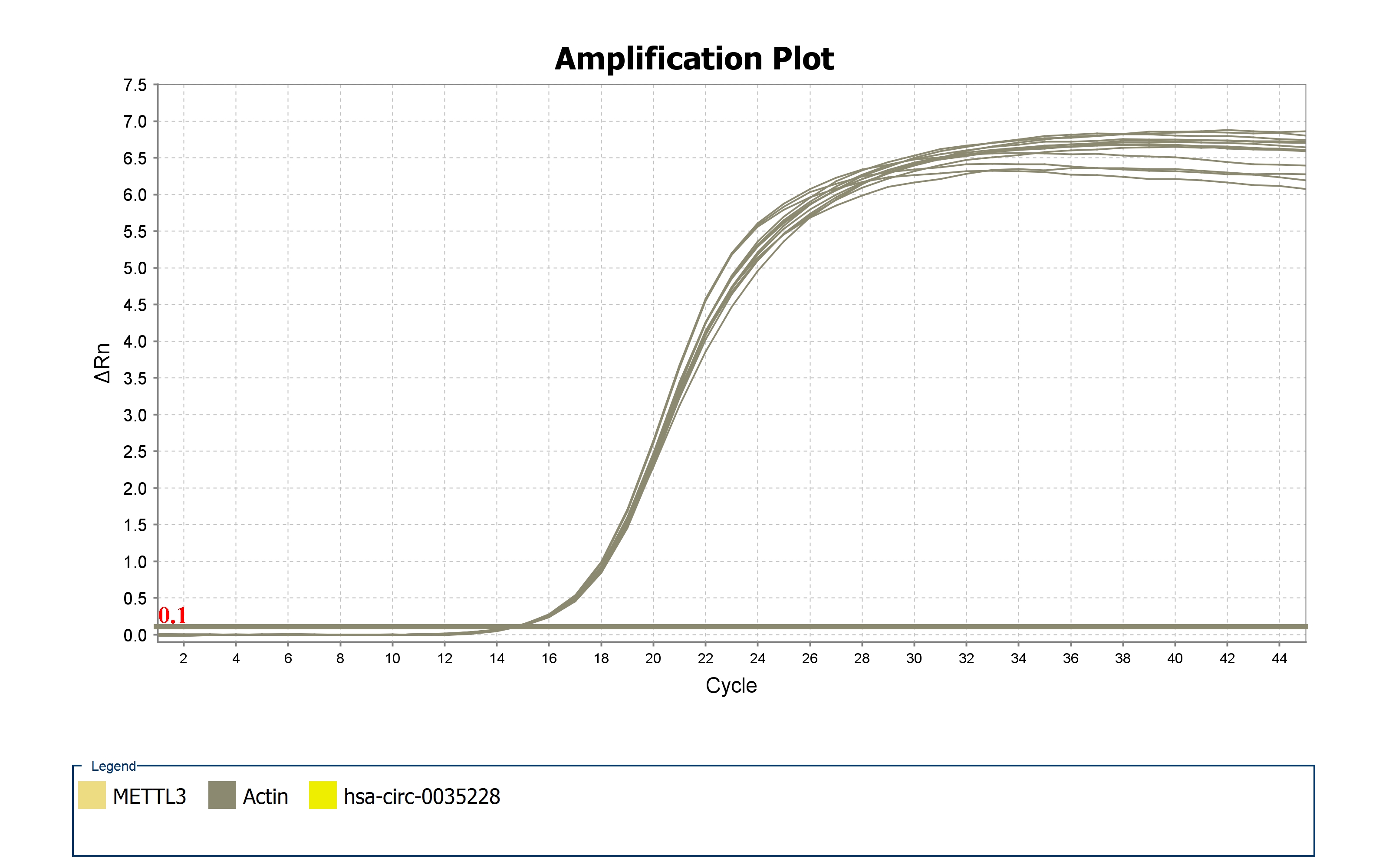

Supplement: Supplemental Information 5 [file peerj-11-14863-s005.zip › Raw data/Fig 1D and 2G/Raw data/Amplification Plot Actin.jpg]

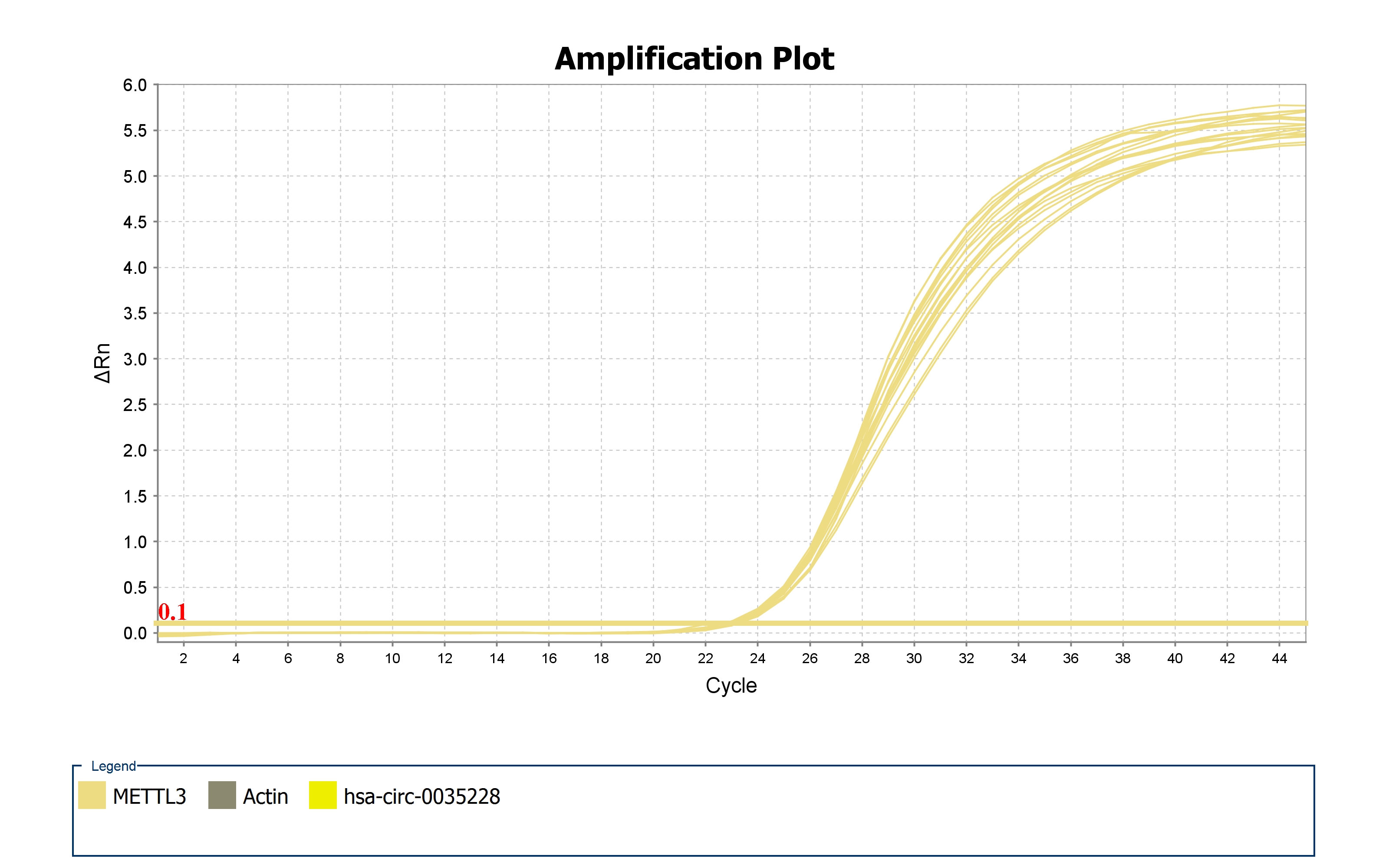

Supplement: Supplemental Information 5 [file peerj-11-14863-s005.zip › Raw data/Fig 1D and 2G/Raw data/Amplification Plot METTL3.jpg]

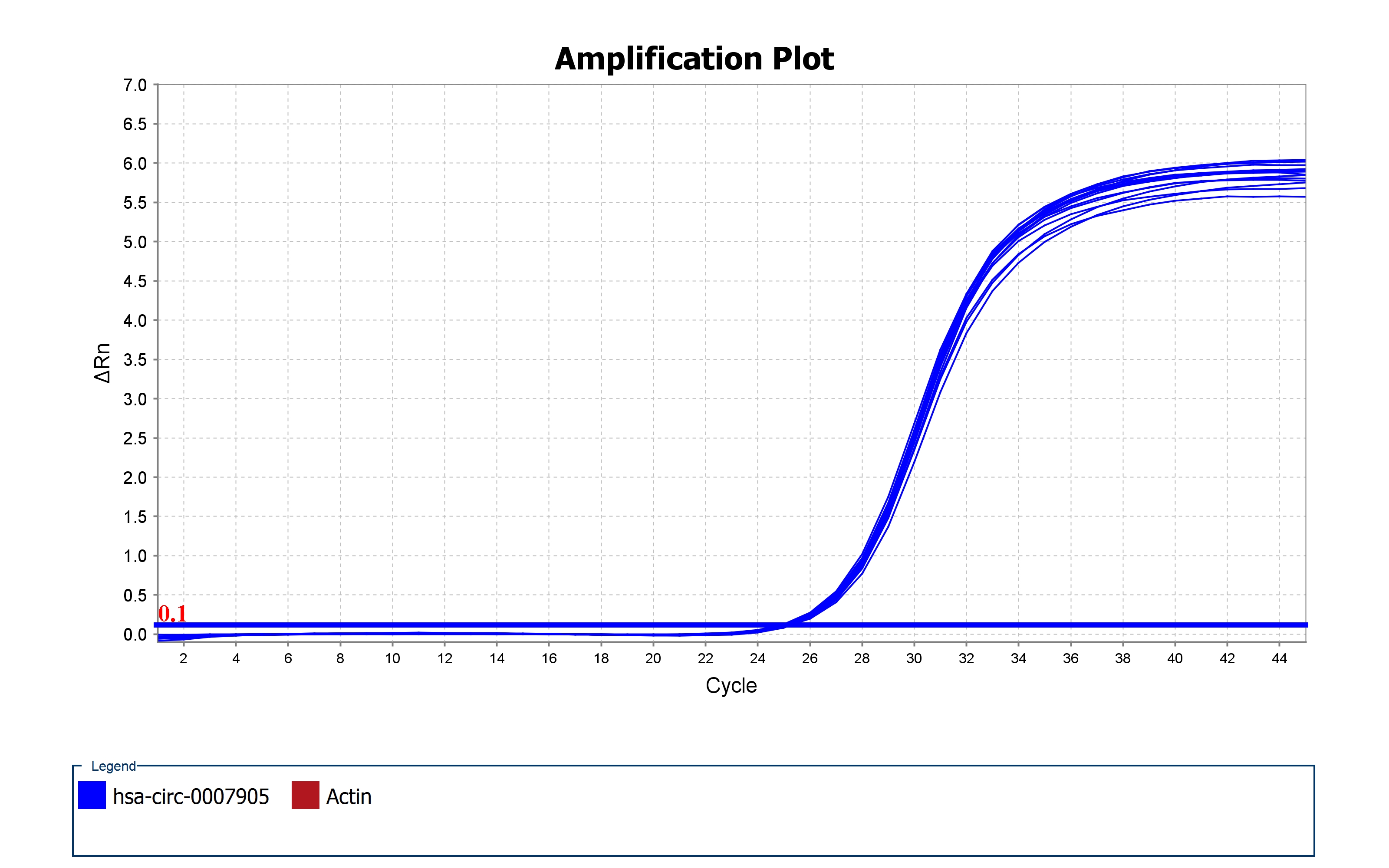

Supplement: Supplemental Information 5 [file peerj-11-14863-s005.zip › Raw data/Fig 1D and 2G/Raw data/Amplification Plot hsa-circ-0007905.jpg]

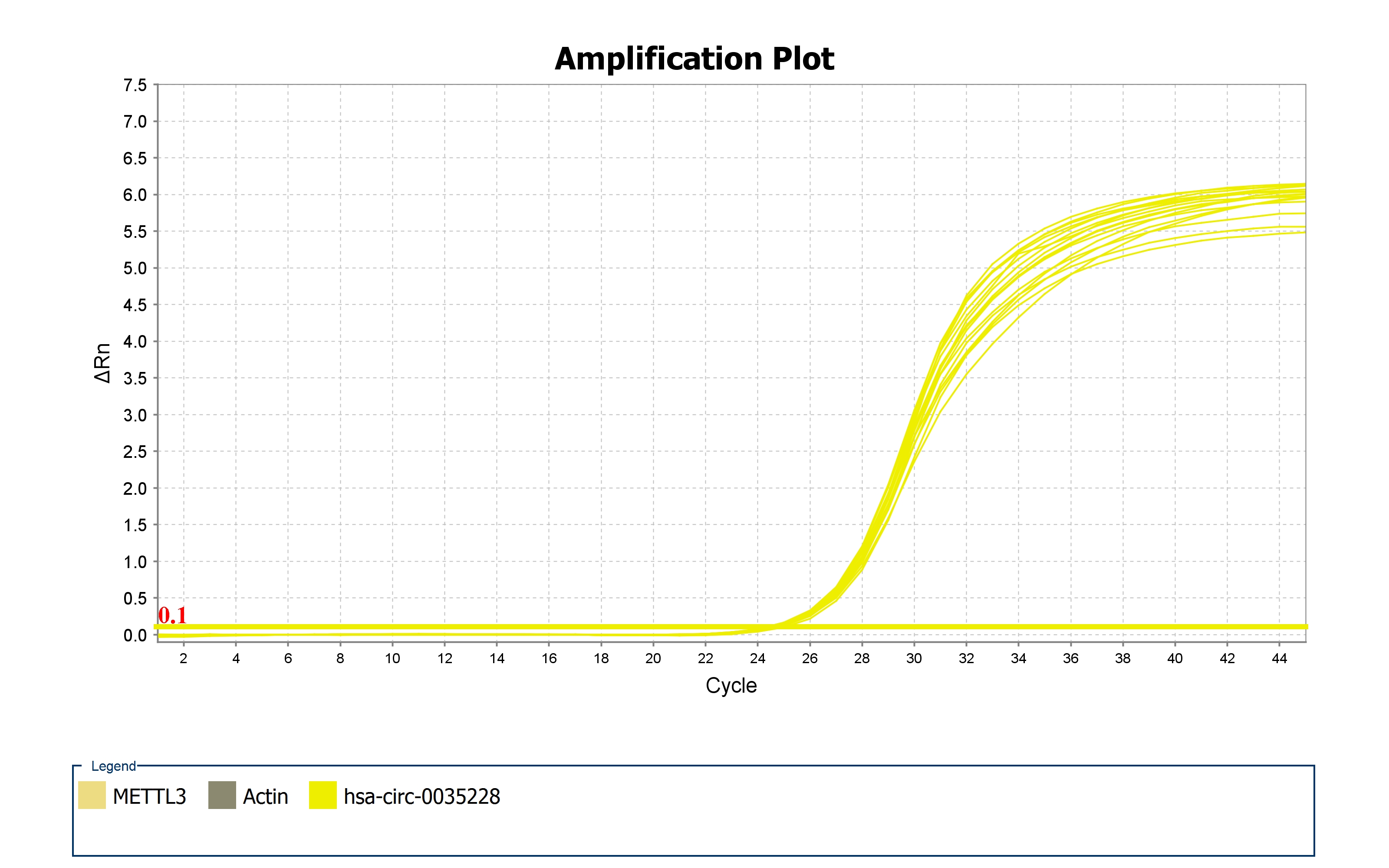

Supplement: Supplemental Information 5 [file peerj-11-14863-s005.zip › Raw data/Fig 1D and 2G/Raw data/Amplification Plot hsa-circ-0035228.jpg]

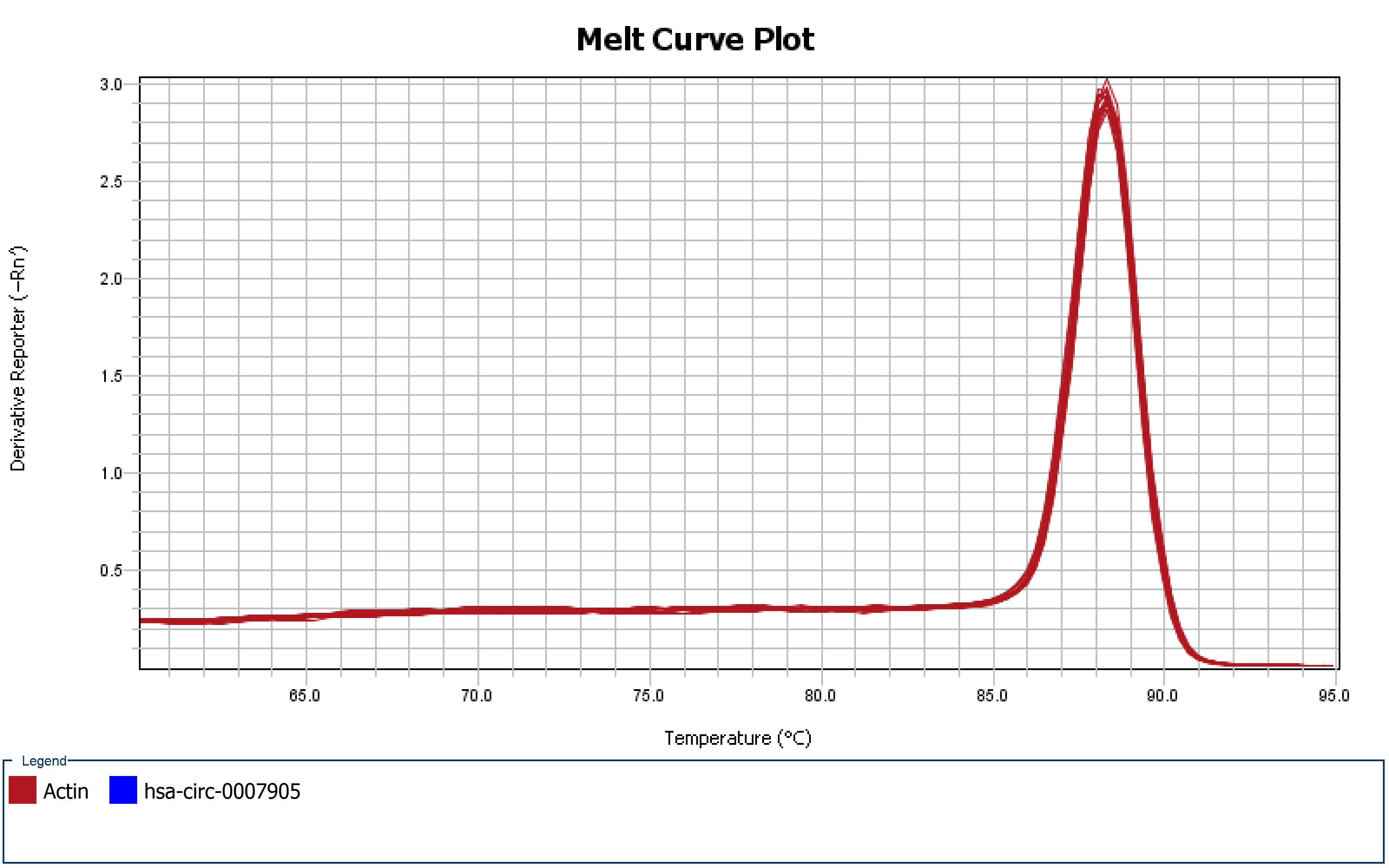

Supplement: Supplemental Information 5 [file peerj-11-14863-s005.zip › Raw data/Fig 1D and 2G/Raw data/Melt Curve Plot Actin (2).jpg]

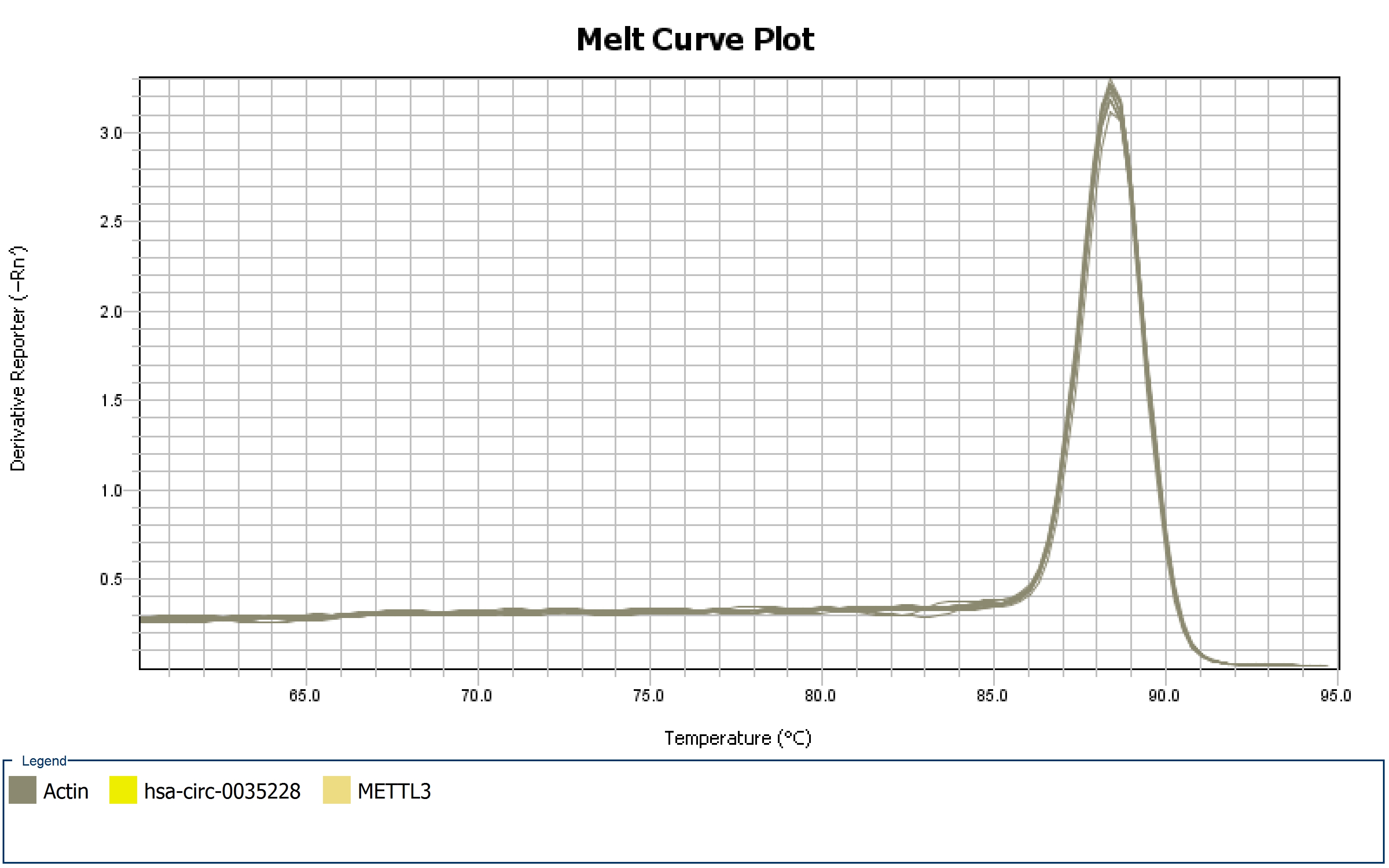

Supplement: Supplemental Information 5 [file peerj-11-14863-s005.zip › Raw data/Fig 1D and 2G/Raw data/Melt Curve Plot Actin.jpg]

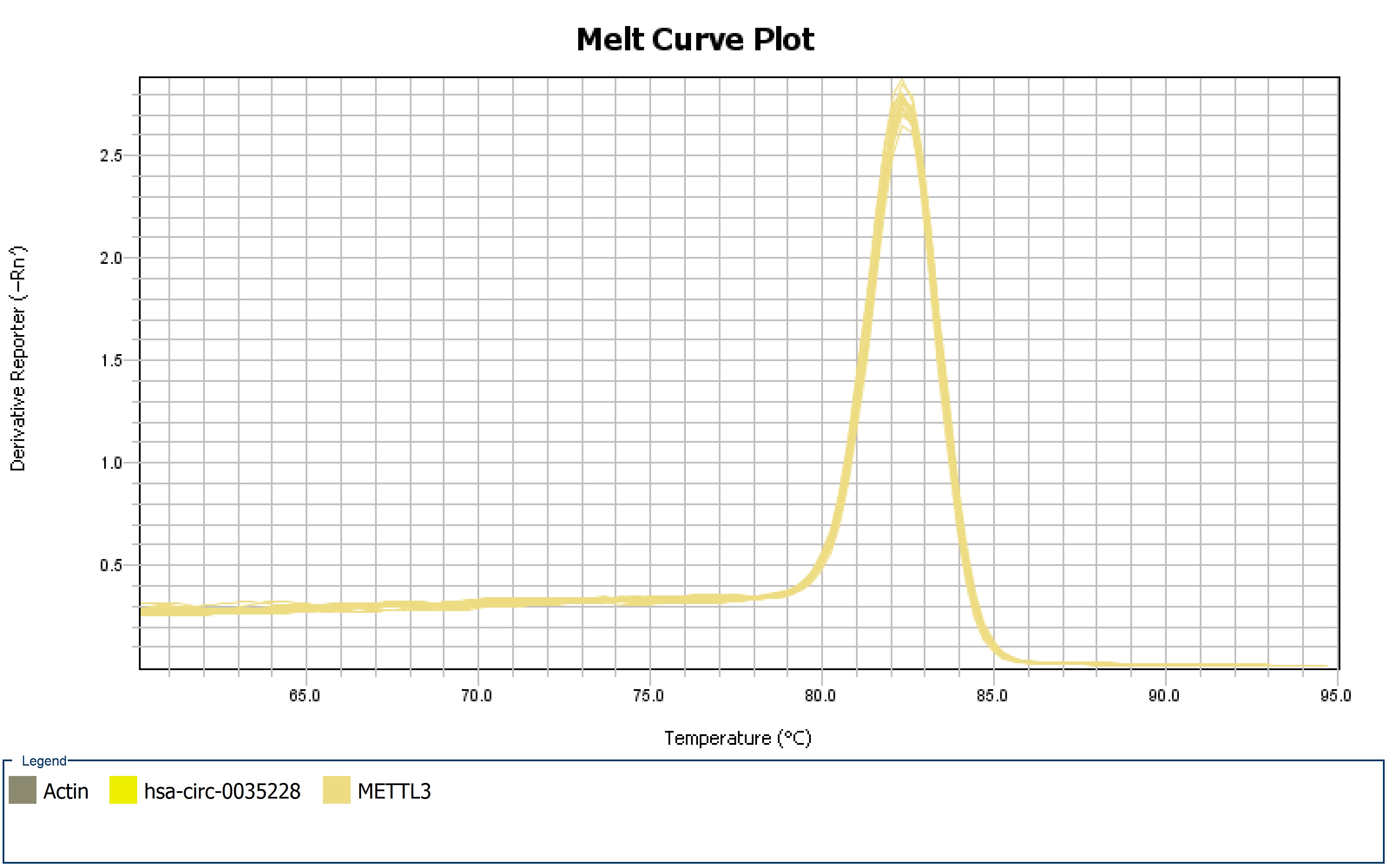

Supplement: Supplemental Information 5 [file peerj-11-14863-s005.zip › Raw data/Fig 1D and 2G/Raw data/Melt Curve Plot METTL3.jpg]

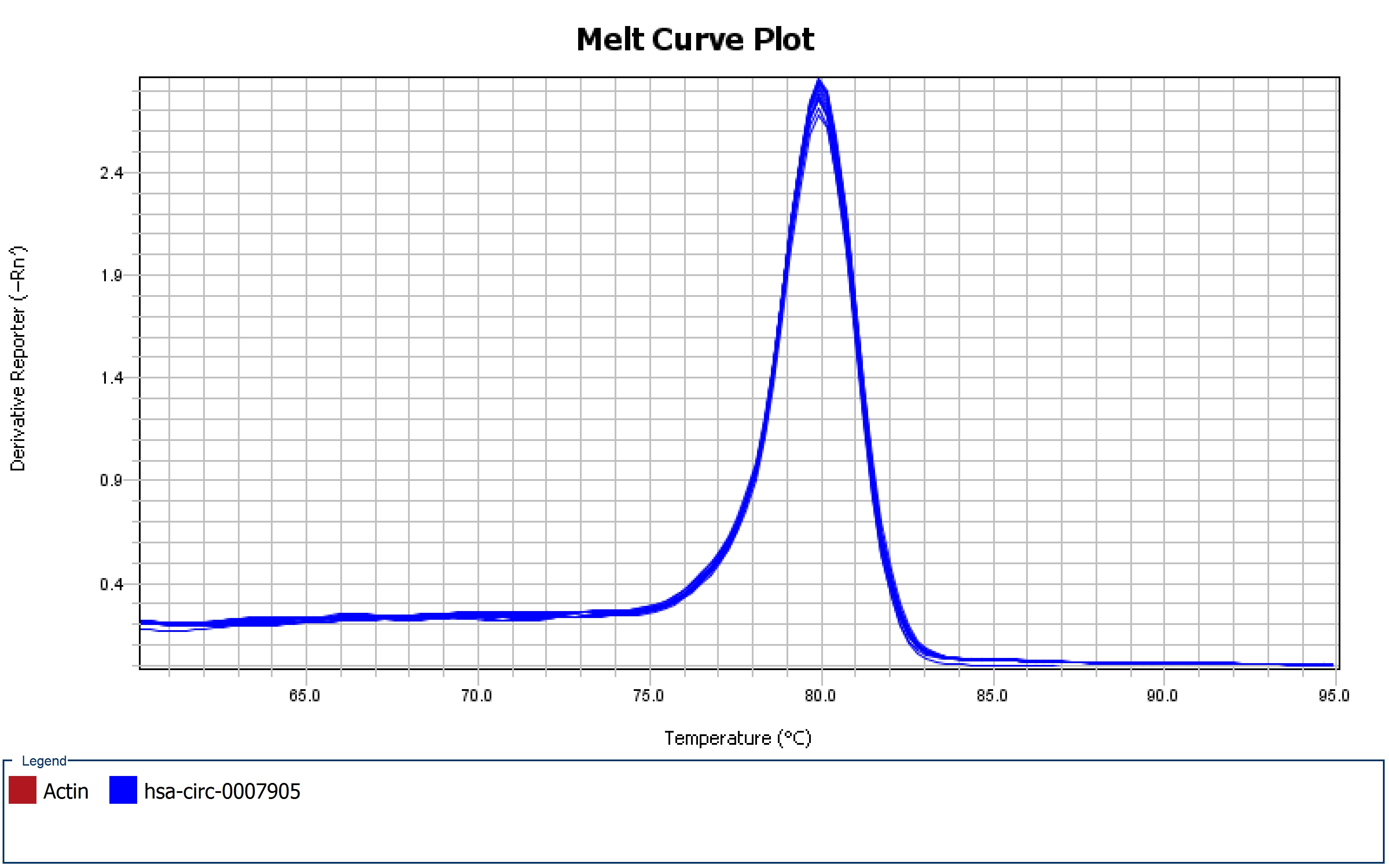

Supplement: Supplemental Information 5 [file peerj-11-14863-s005.zip › Raw data/Fig 1D and 2G/Raw data/Melt Curve Plot hsa-circ-0007905.jpg]

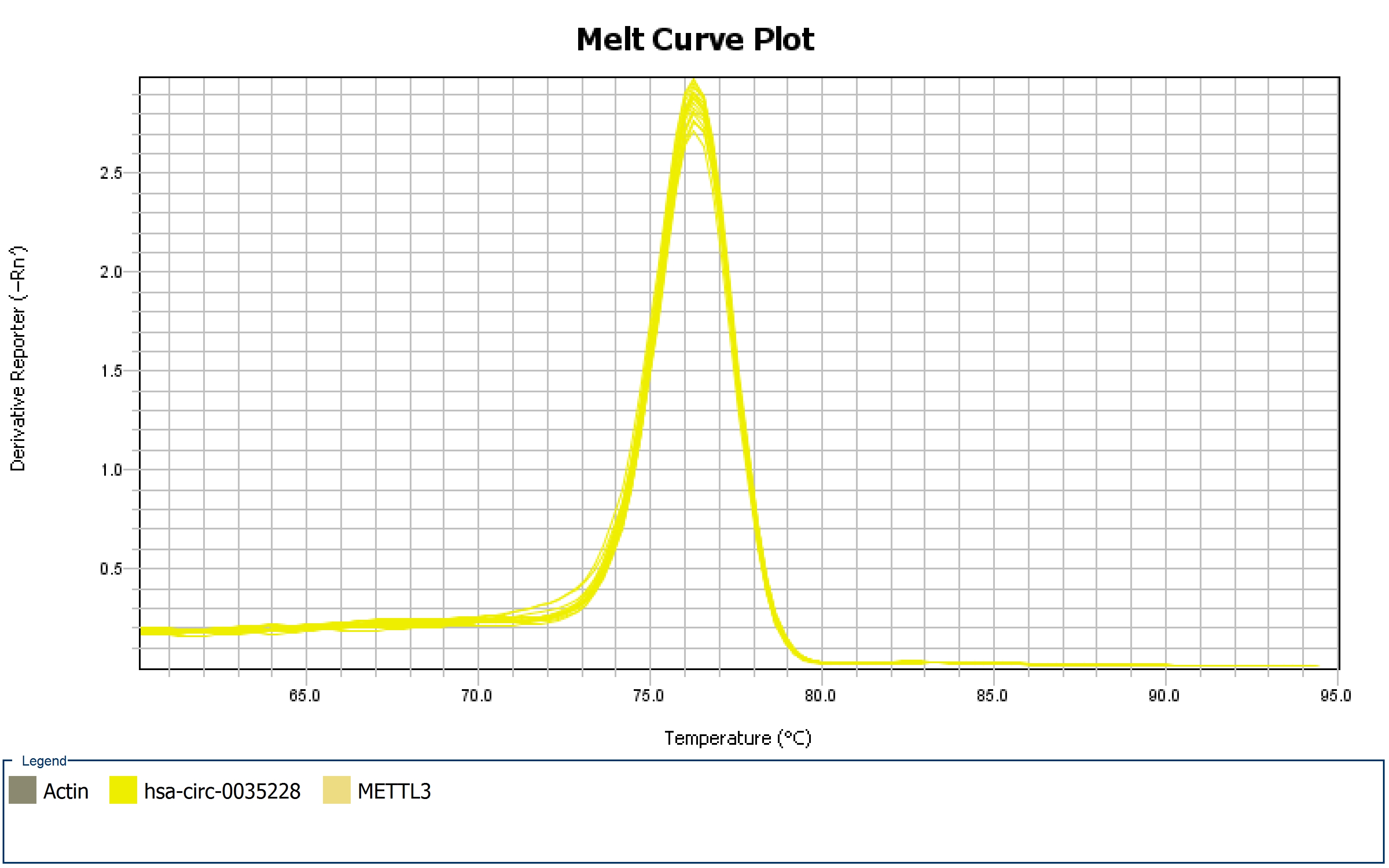

Supplement: Supplemental Information 5 [file peerj-11-14863-s005.zip › Raw data/Fig 1D and 2G/Raw data/Melt Curve Plot hsa-circ-0035228.jpg]

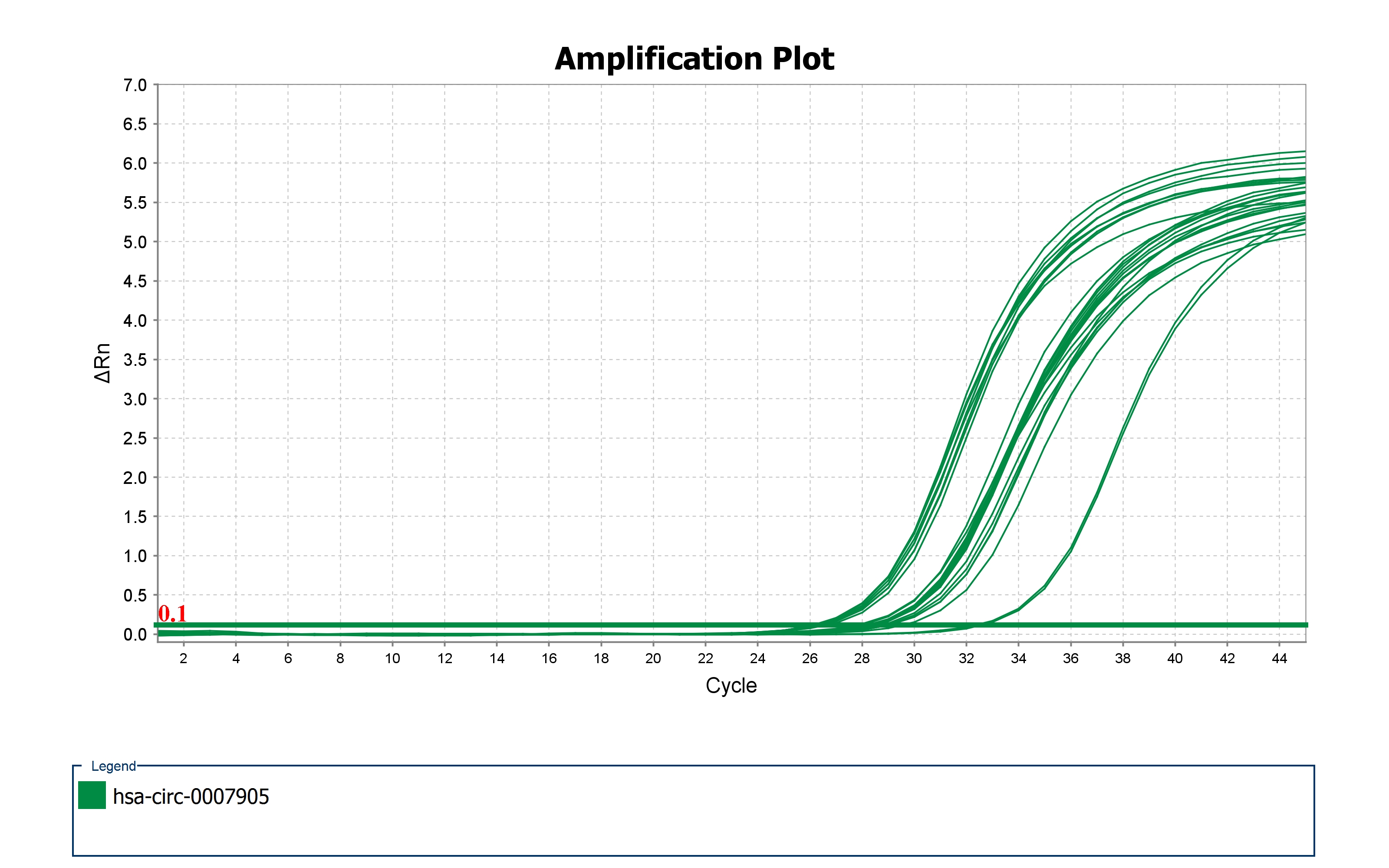

Supplement: Supplemental Information 5 [file peerj-11-14863-s005.zip › Raw data/Fig 1E/Raw data/Amplification Plot hsa-circ-0007905.jpg]

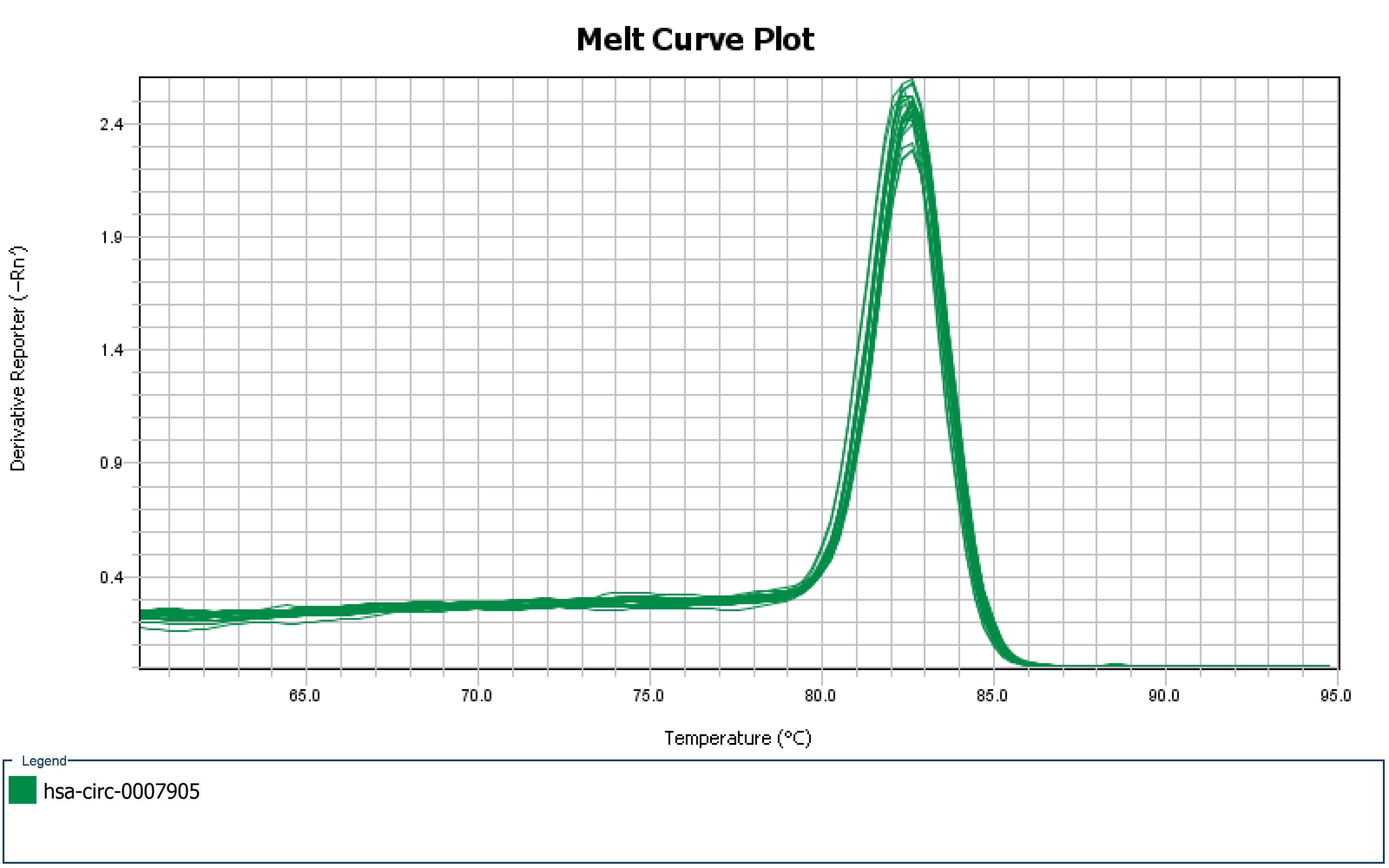

Supplement: Supplemental Information 5 [file peerj-11-14863-s005.zip › Raw data/Fig 1E/Raw data/Melt Curve Plot hsa-circ-0007905.jpg]

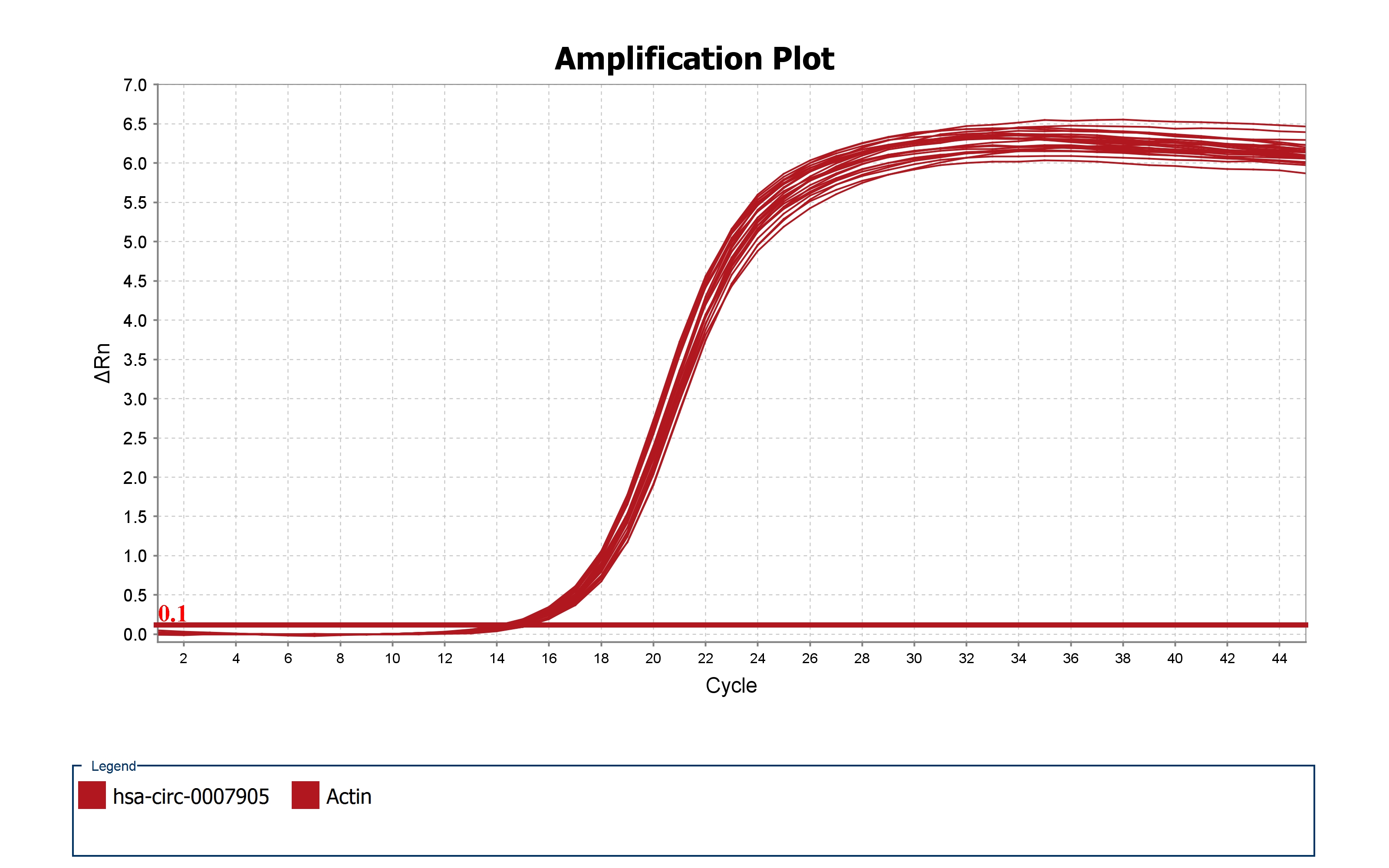

Supplement: Supplemental Information 5 [file peerj-11-14863-s005.zip › Raw data/Fig 2A/Raw data/Amplification Plot Actin.jpg]

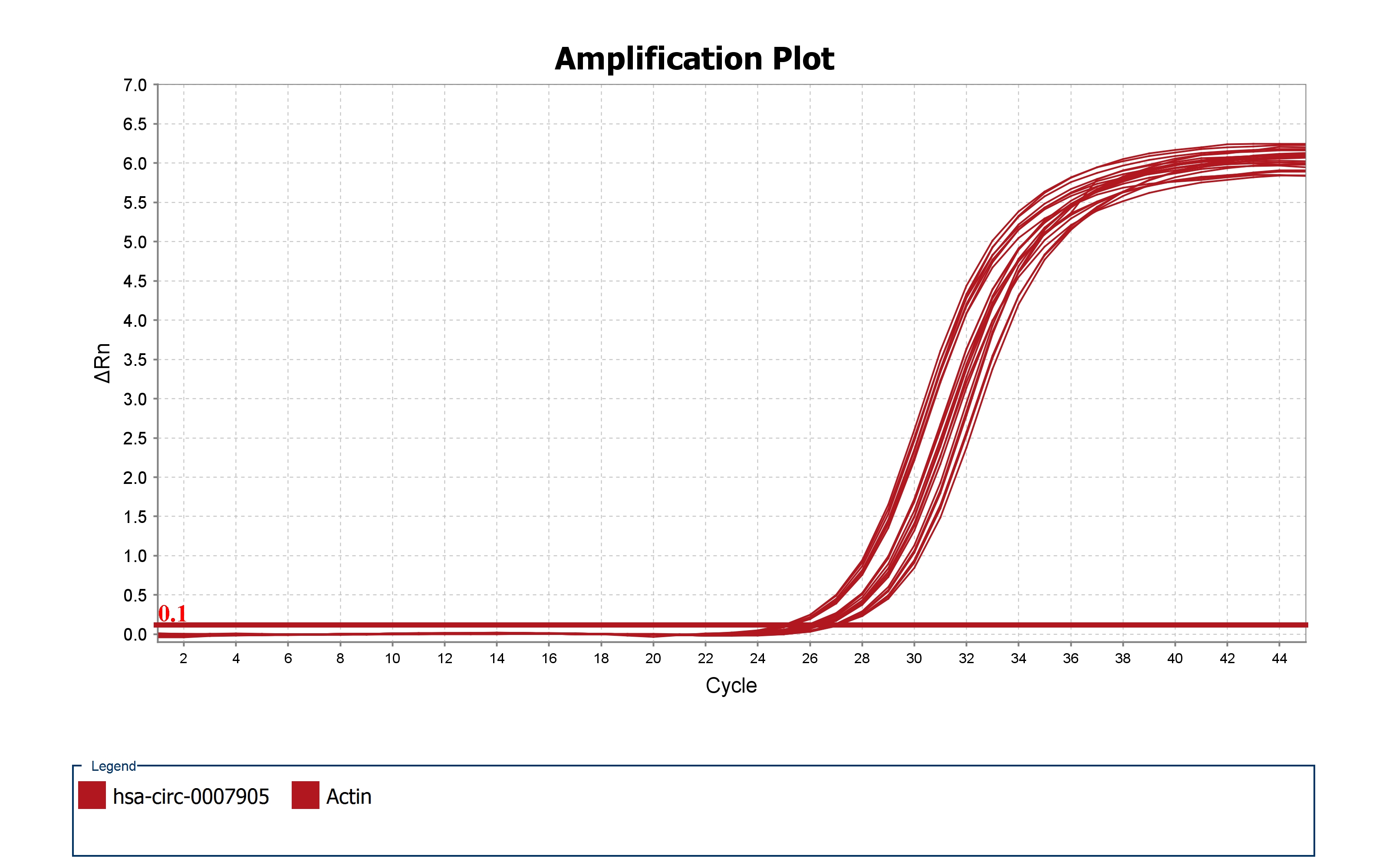

Supplement: Supplemental Information 5 [file peerj-11-14863-s005.zip › Raw data/Fig 2A/Raw data/Amplification Plot hsa-circ-0007905.jpg]

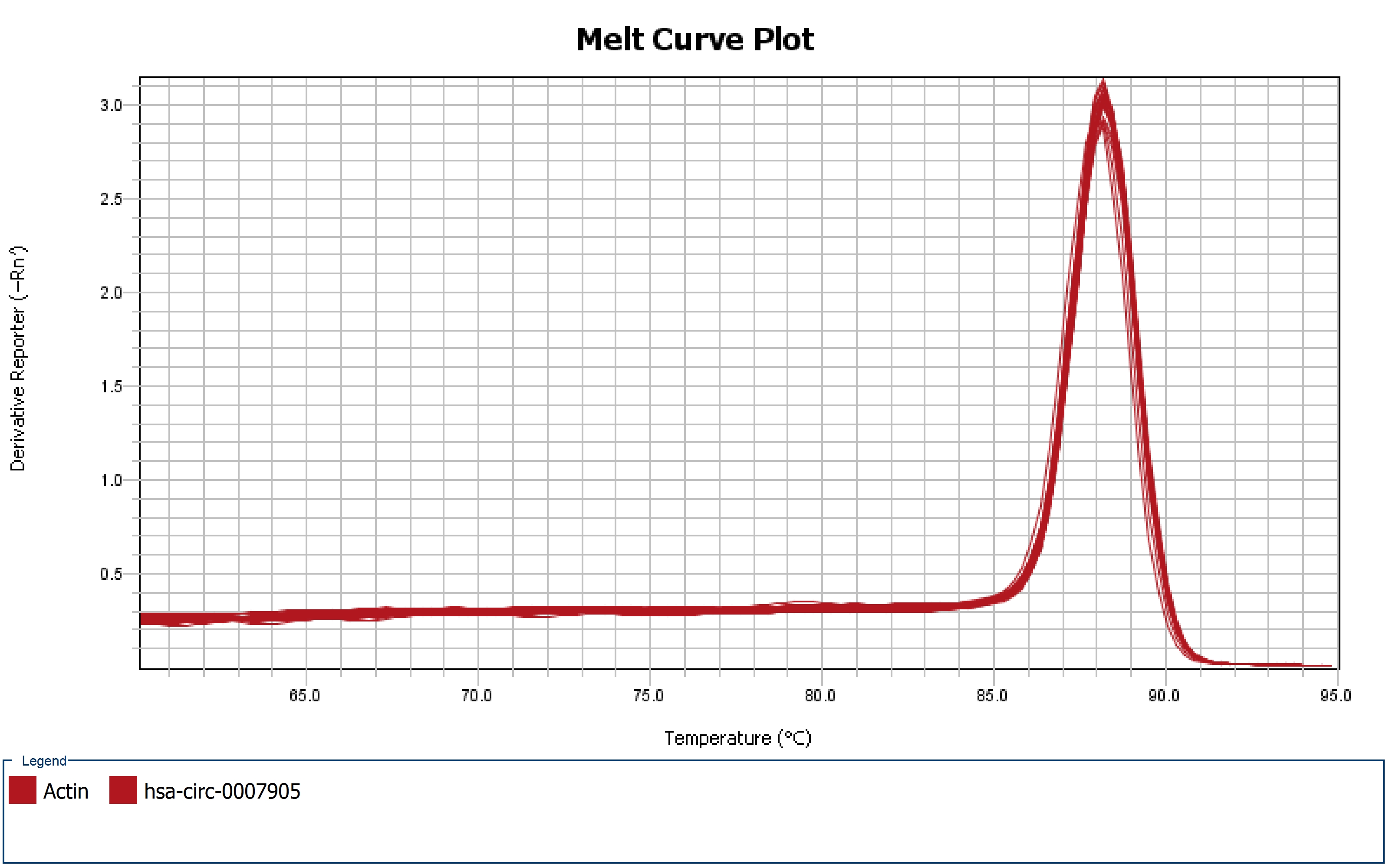

Supplement: Supplemental Information 5 [file peerj-11-14863-s005.zip › Raw data/Fig 2A/Raw data/Melt Curve Plot Actin.jpg]

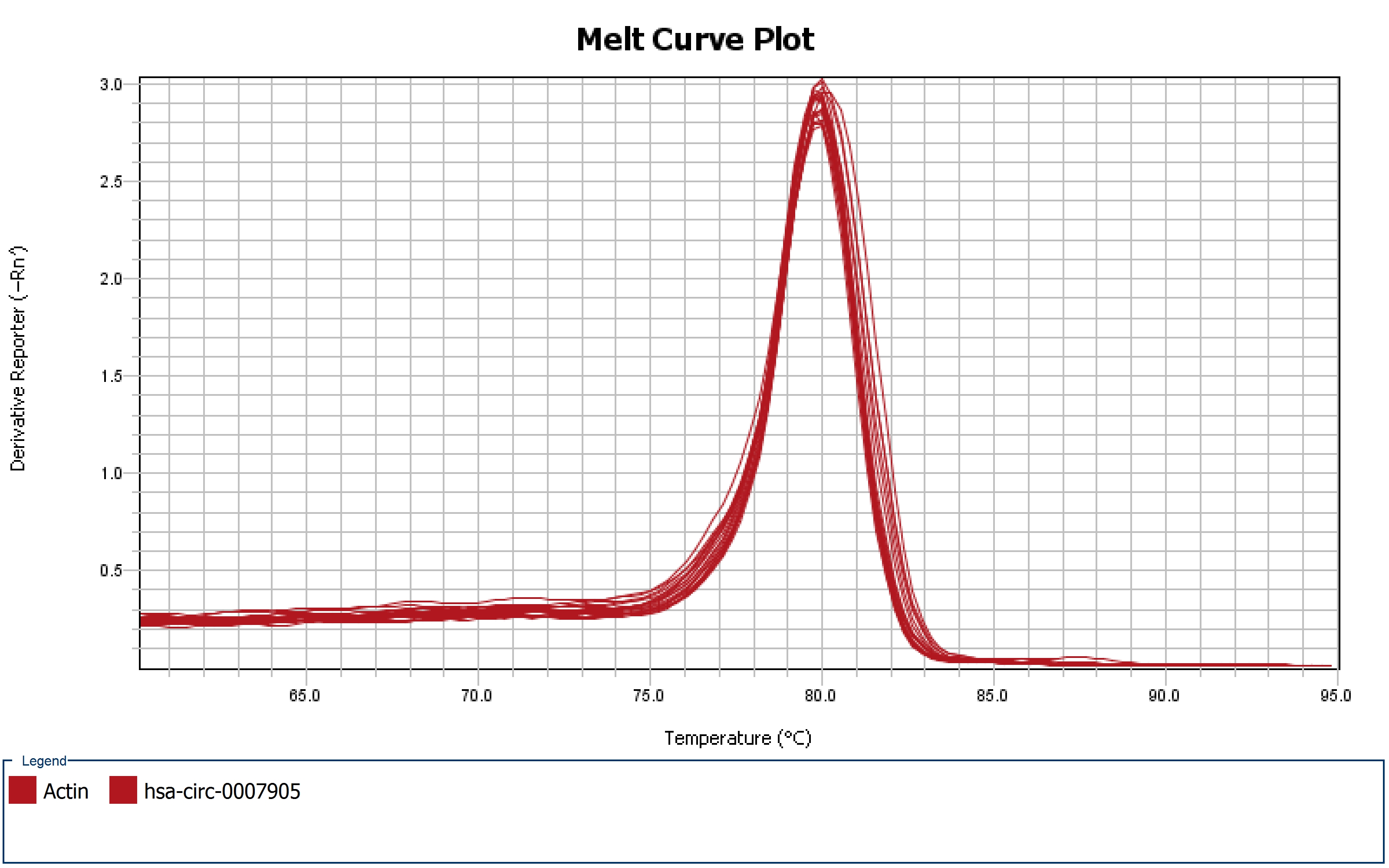

Supplement: Supplemental Information 5 [file peerj-11-14863-s005.zip › Raw data/Fig 2A/Raw data/Melt Curve Plot hsa-circ-0007905.jpg]

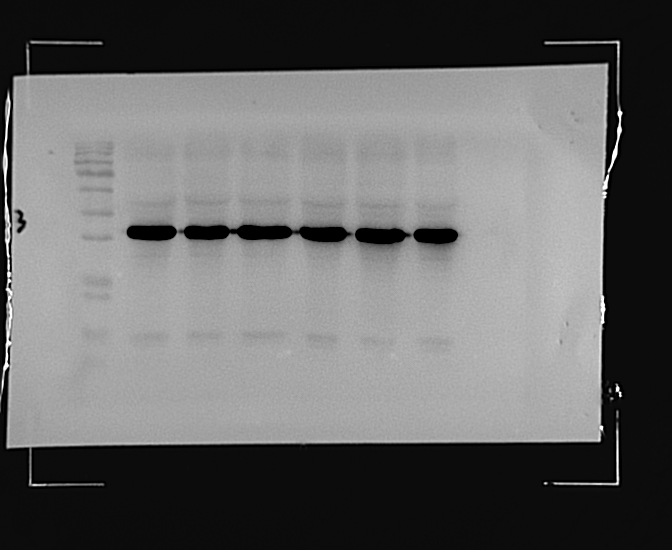

Supplement: Supplemental Information 5 [file peerj-11-14863-s005.zip › Raw data/Fig 2H/GAPDH.tif]

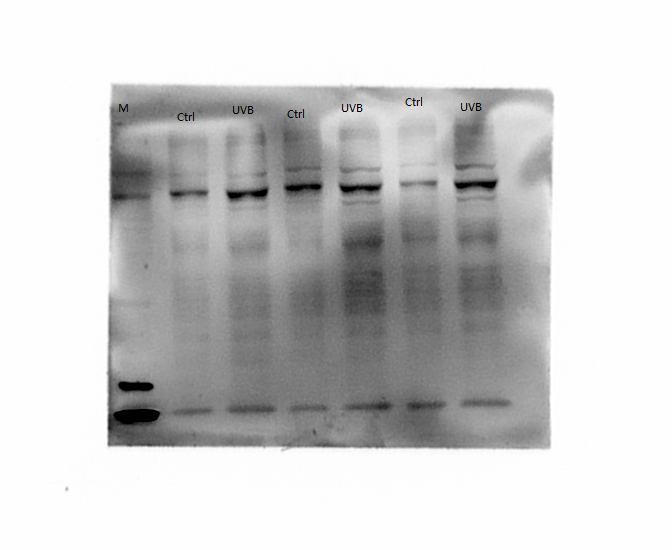

Supplement: Supplemental Information 5 [file peerj-11-14863-s005.zip › Raw data/Fig 2H/Mettl3.tif]

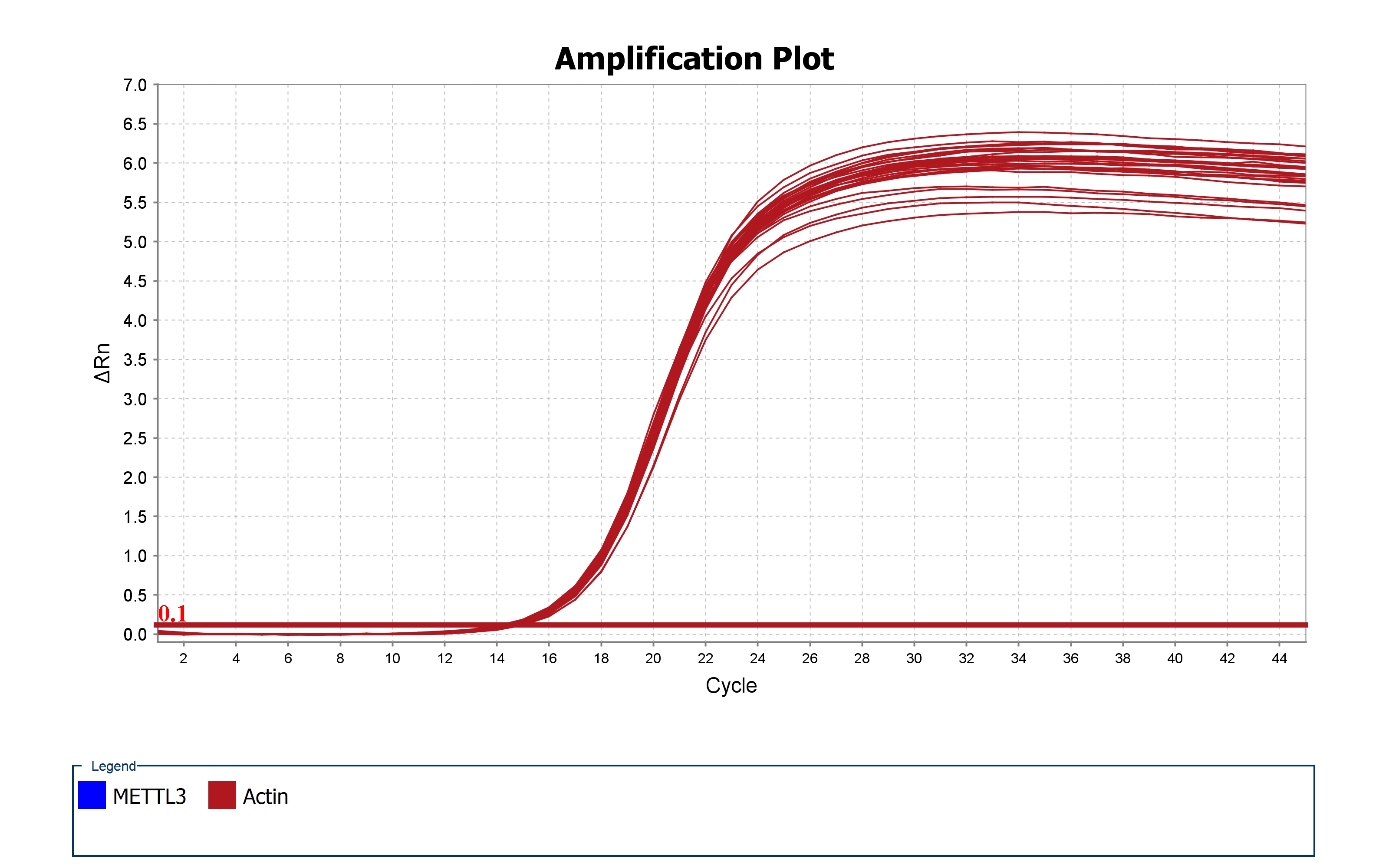

Supplement: Supplemental Information 5 [file peerj-11-14863-s005.zip › Raw data/Fig 3A/Raw data/Amplification Plot Actin.jpg]

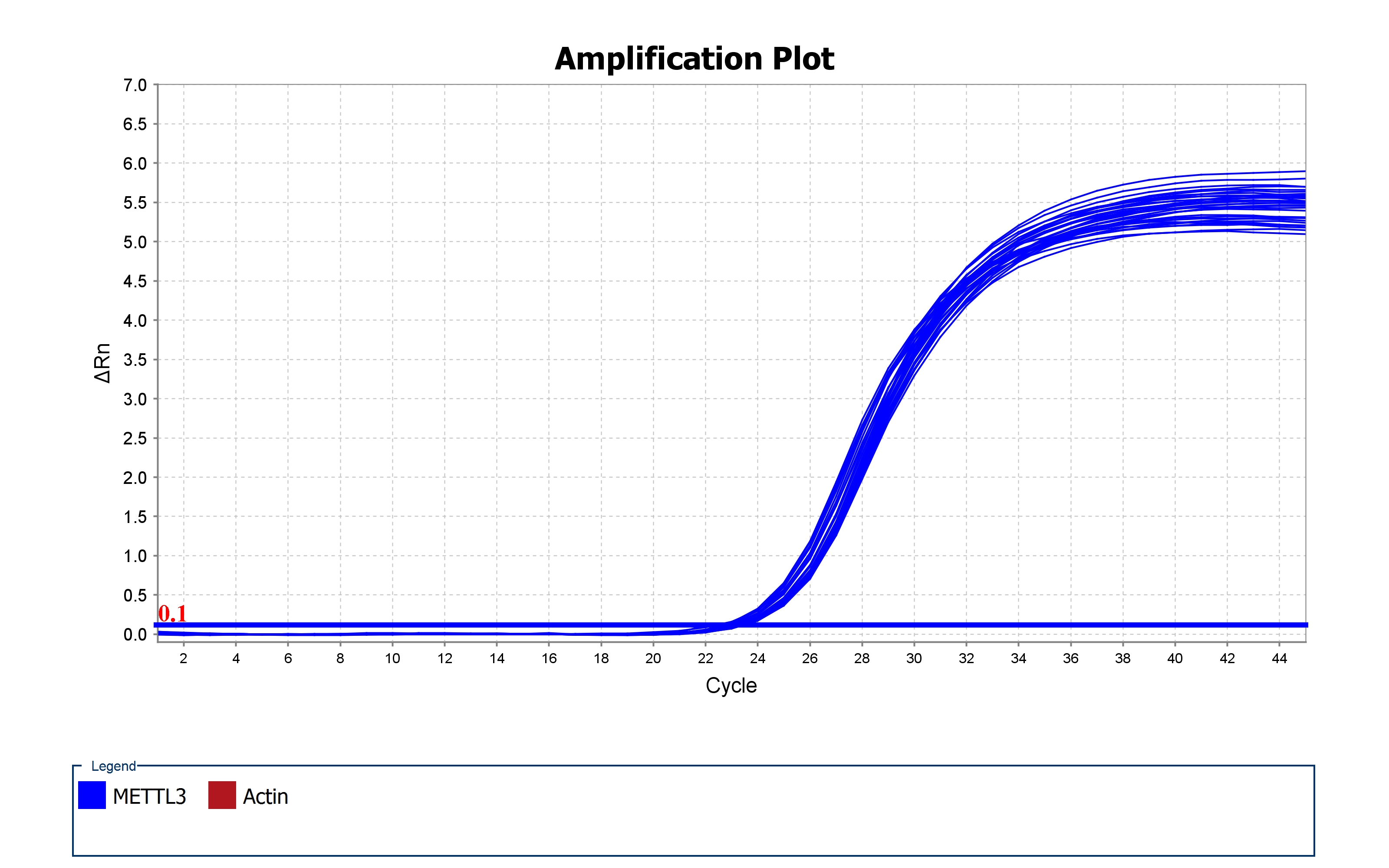

Supplement: Supplemental Information 5 [file peerj-11-14863-s005.zip › Raw data/Fig 3A/Raw data/Amplification Plot METTL3.jpg]

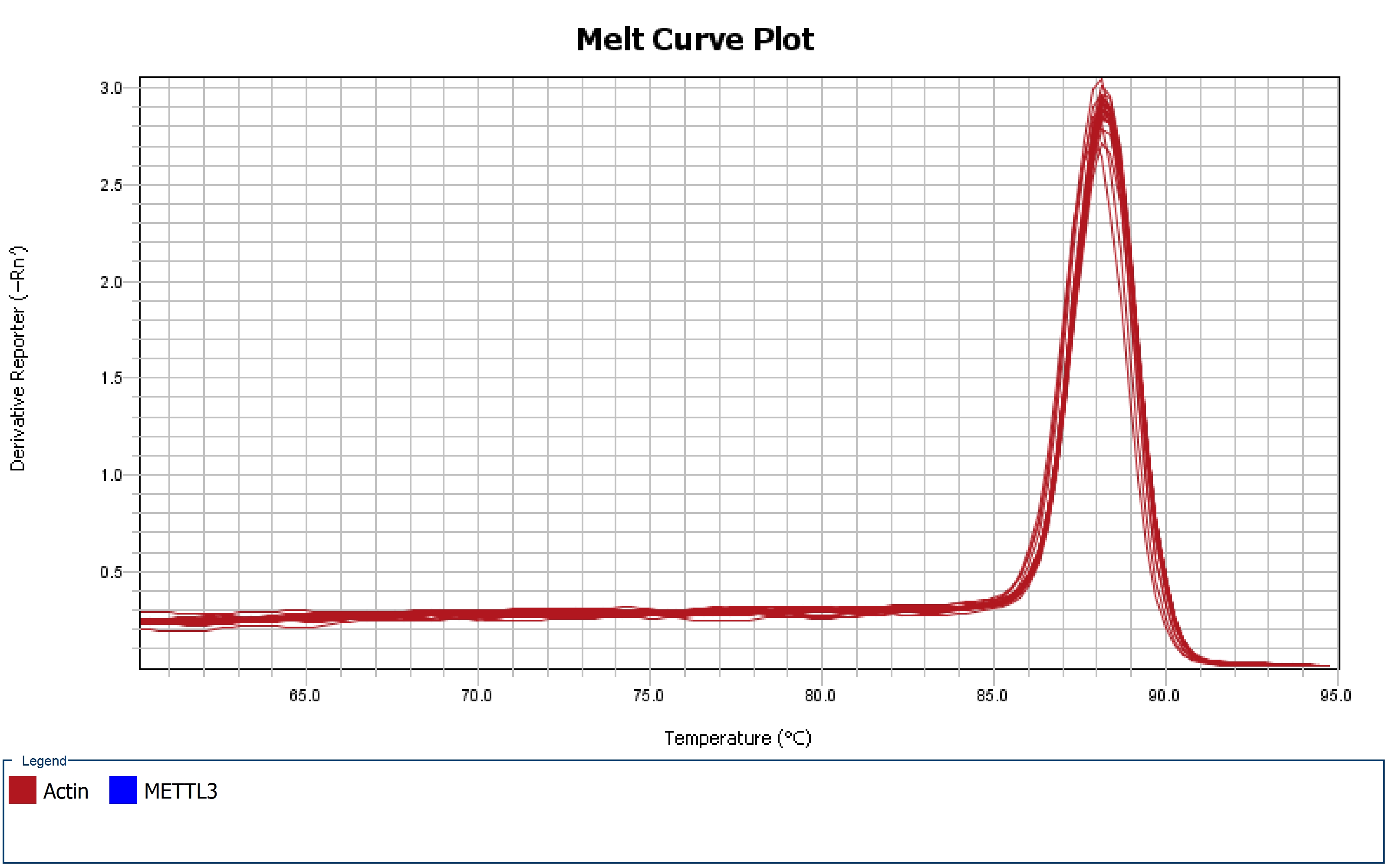

Supplement: Supplemental Information 5 [file peerj-11-14863-s005.zip › Raw data/Fig 3A/Raw data/Melt Curve Plot Actin.jpg]

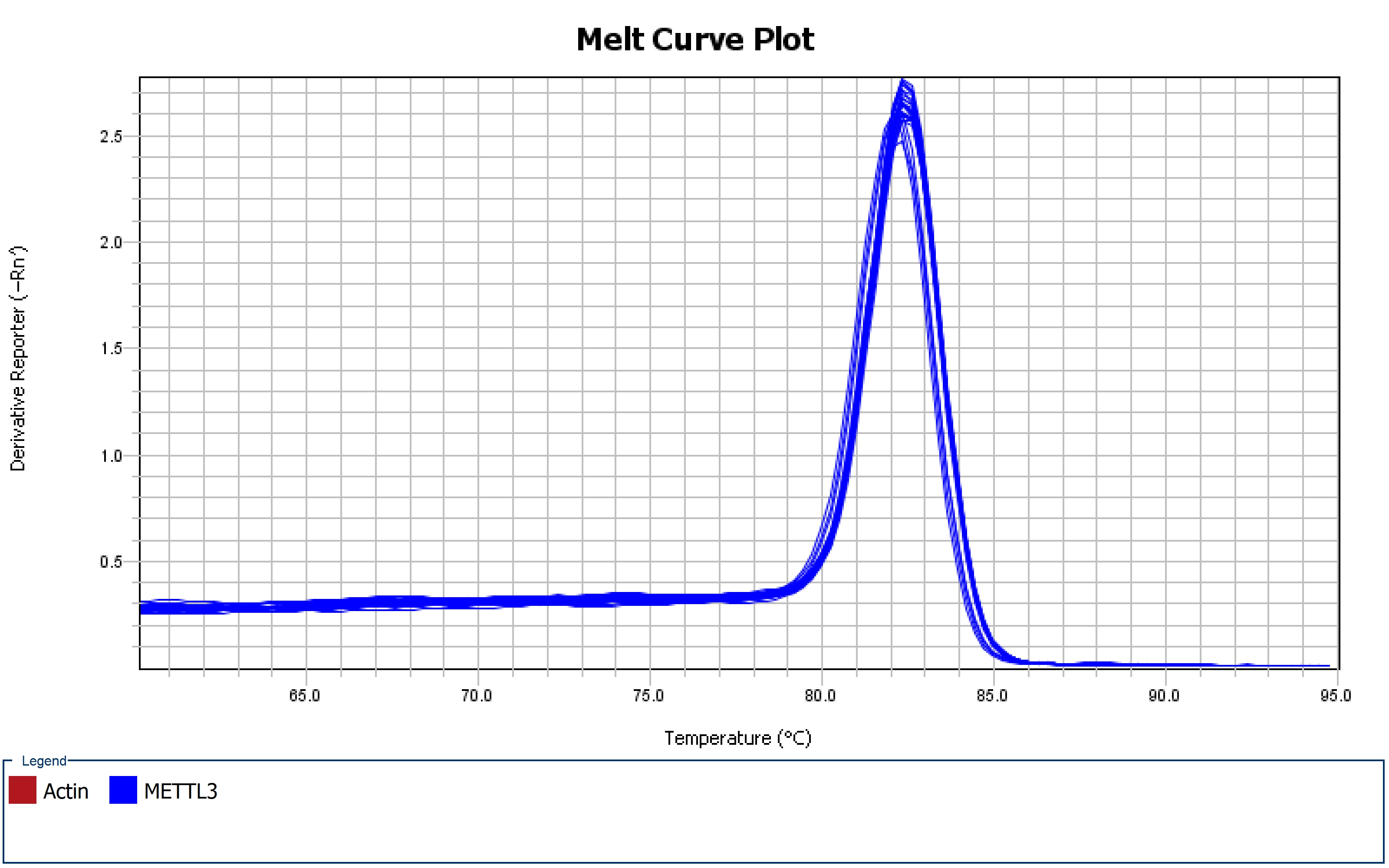

Supplement: Supplemental Information 5 [file peerj-11-14863-s005.zip › Raw data/Fig 3A/Raw data/Melt Curve Plot METTL3.jpg]

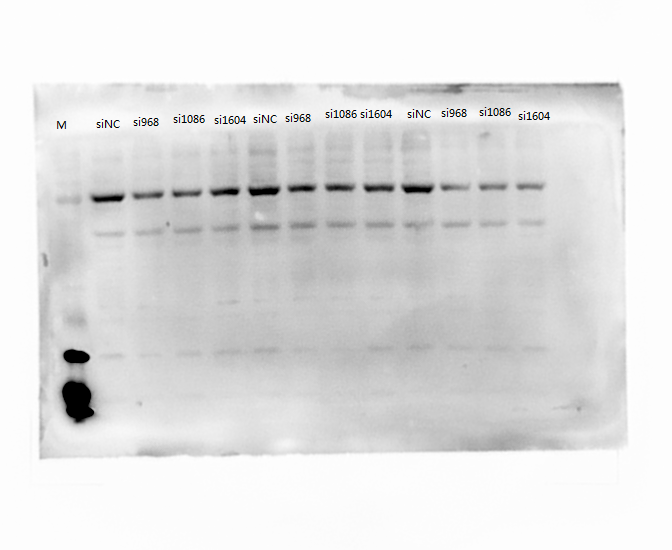

Supplement: Supplemental Information 5 [file peerj-11-14863-s005.zip › Raw data/Fig 3B/3B METTL3.tif]

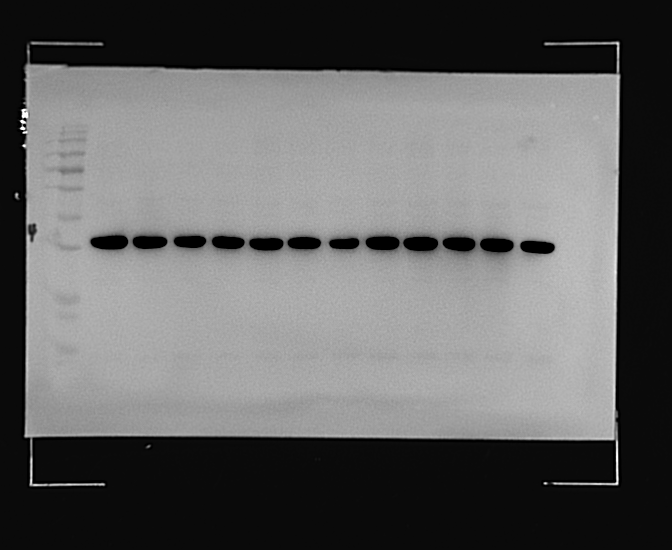

Supplement: Supplemental Information 5 [file peerj-11-14863-s005.zip › Raw data/Fig 3B/GAPDH.tif]

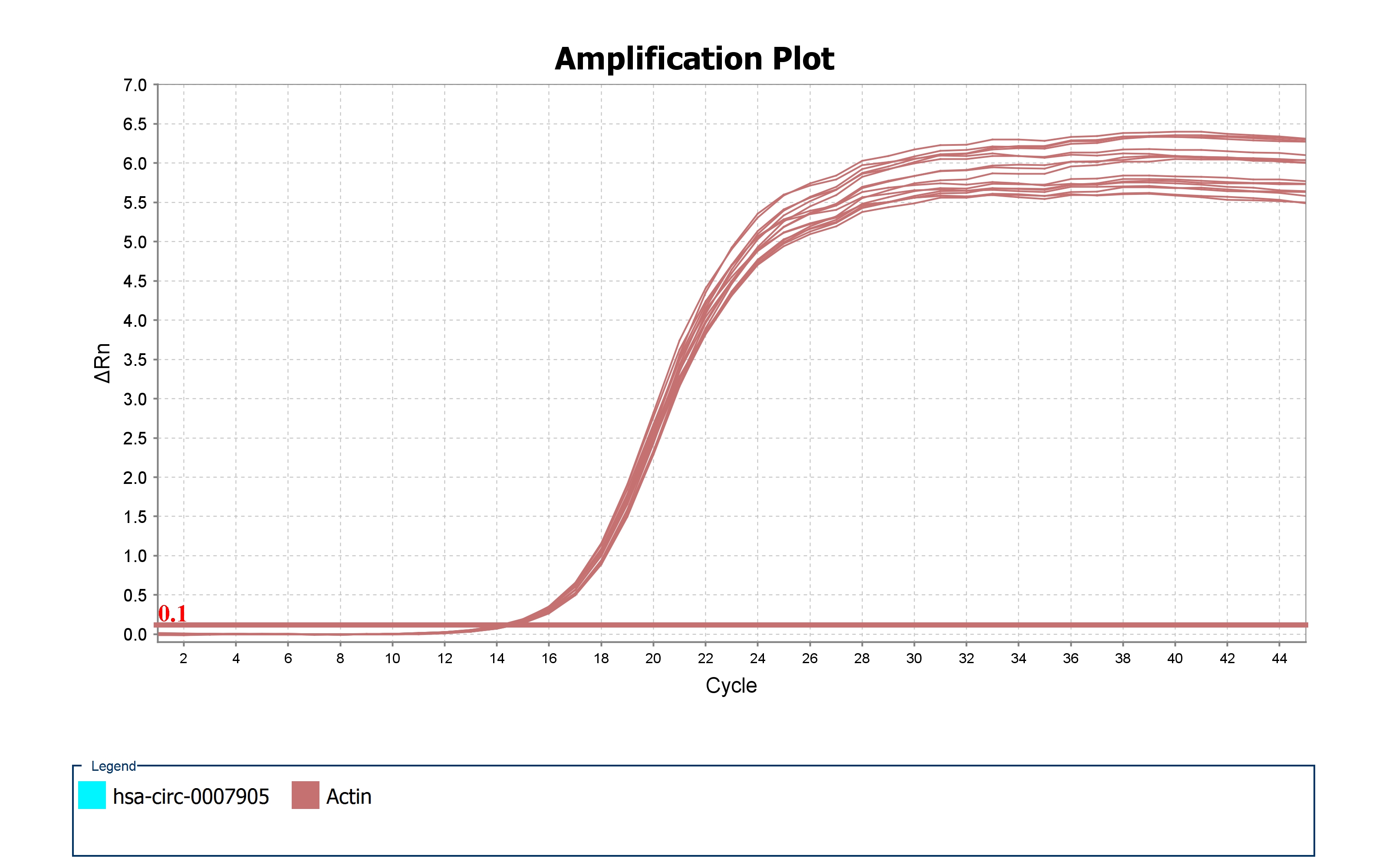

Supplement: Supplemental Information 5 [file peerj-11-14863-s005.zip › Raw data/Fig 3C/Raw data/Amplification Plot Actin.jpg]

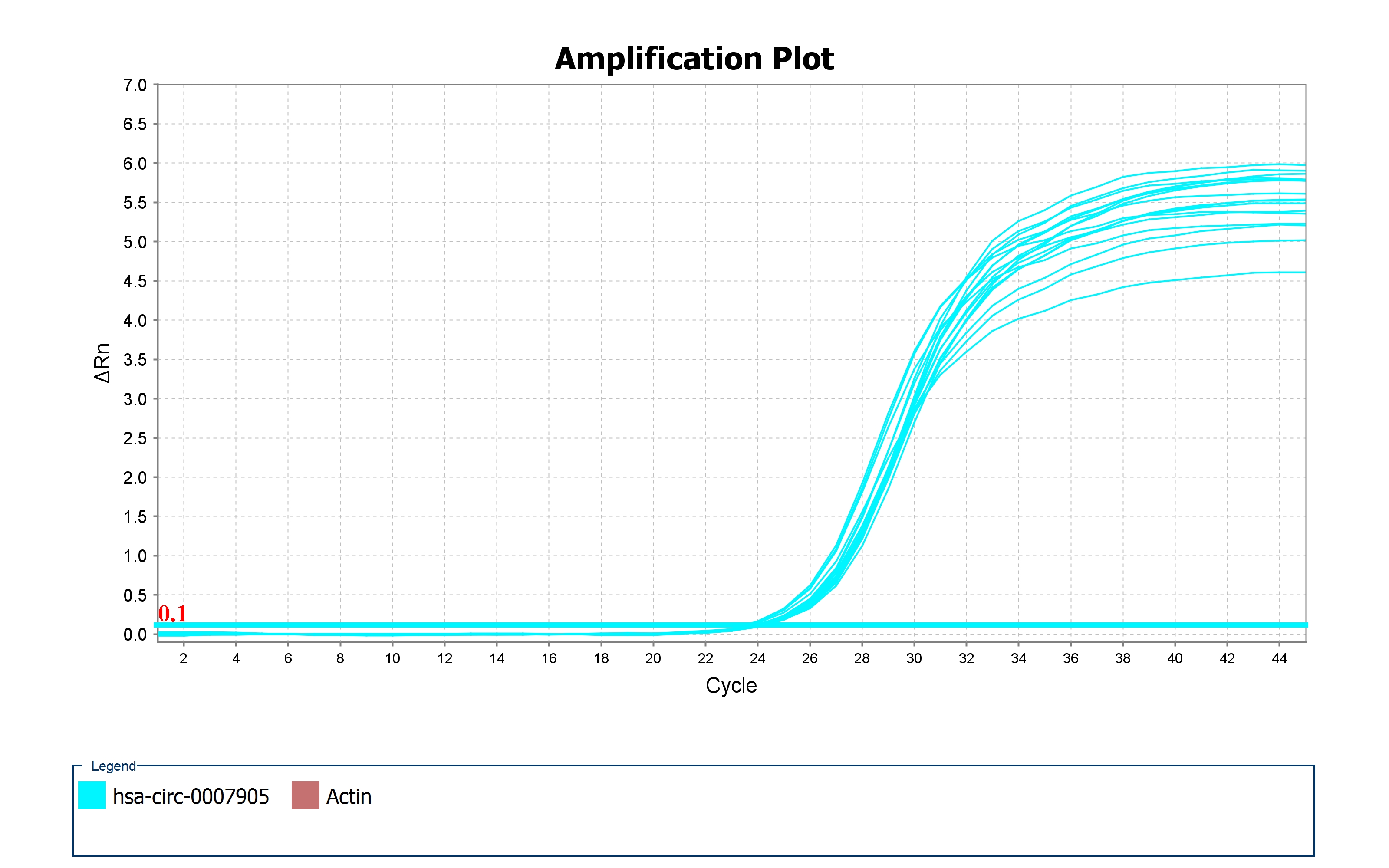

Supplement: Supplemental Information 5 [file peerj-11-14863-s005.zip › Raw data/Fig 3C/Raw data/Amplification Plot hsa-circ-0007905.jpg]

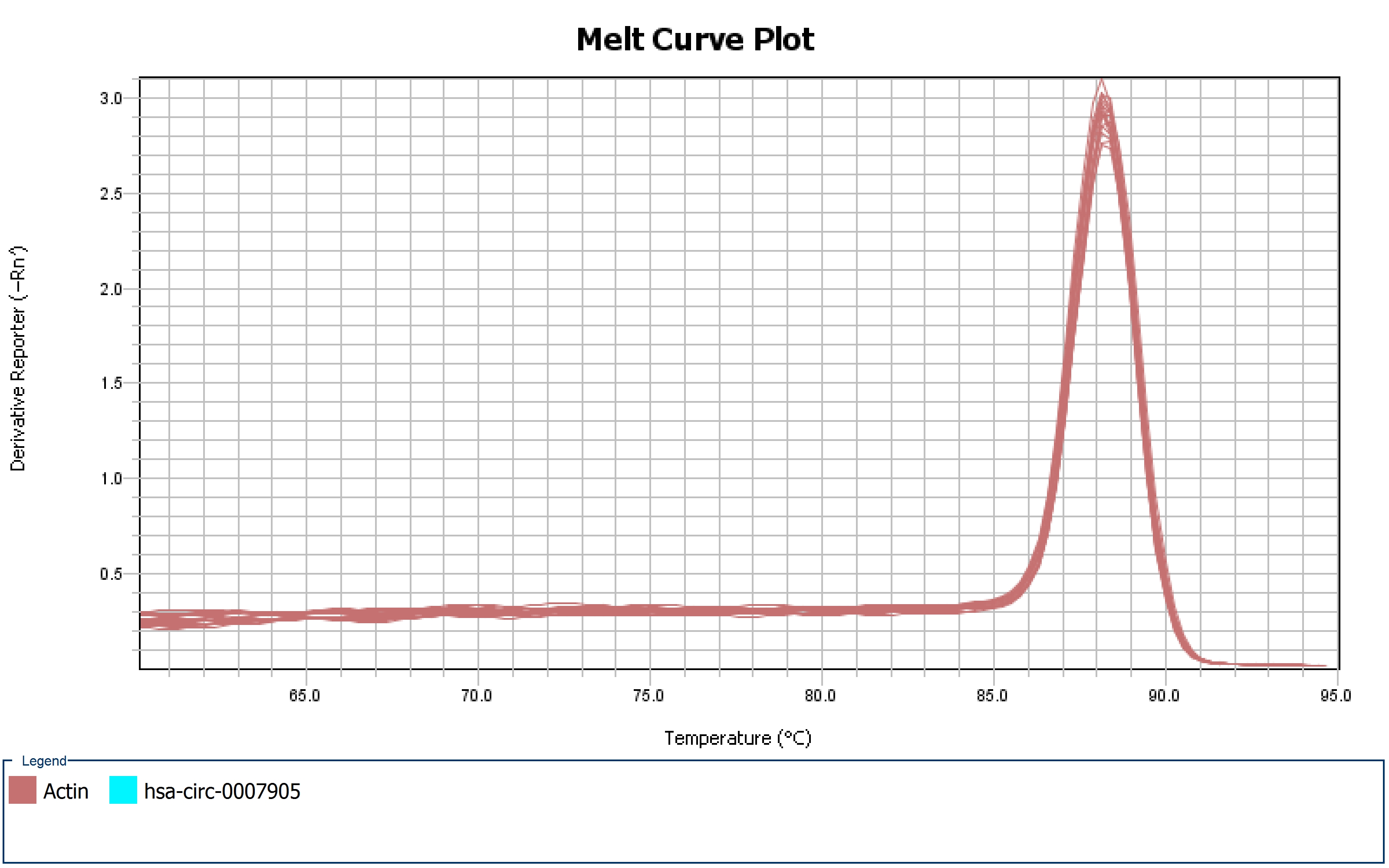

Supplement: Supplemental Information 5 [file peerj-11-14863-s005.zip › Raw data/Fig 3C/Raw data/Melt Curve Plot Actin.jpg]

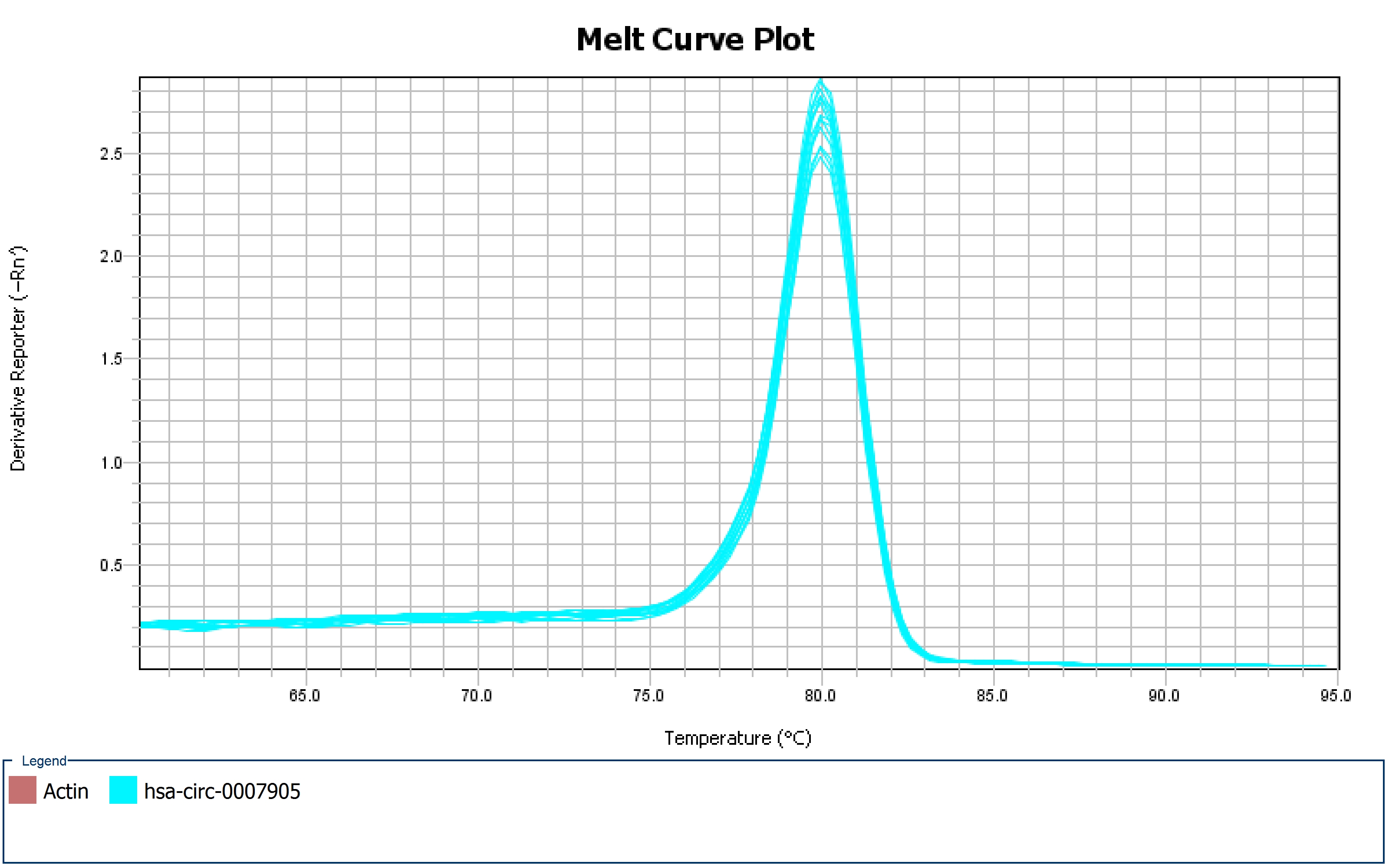

Supplement: Supplemental Information 5 [file peerj-11-14863-s005.zip › Raw data/Fig 3C/Raw data/Melt Curve Plot hsa-circ-0007905.jpg]

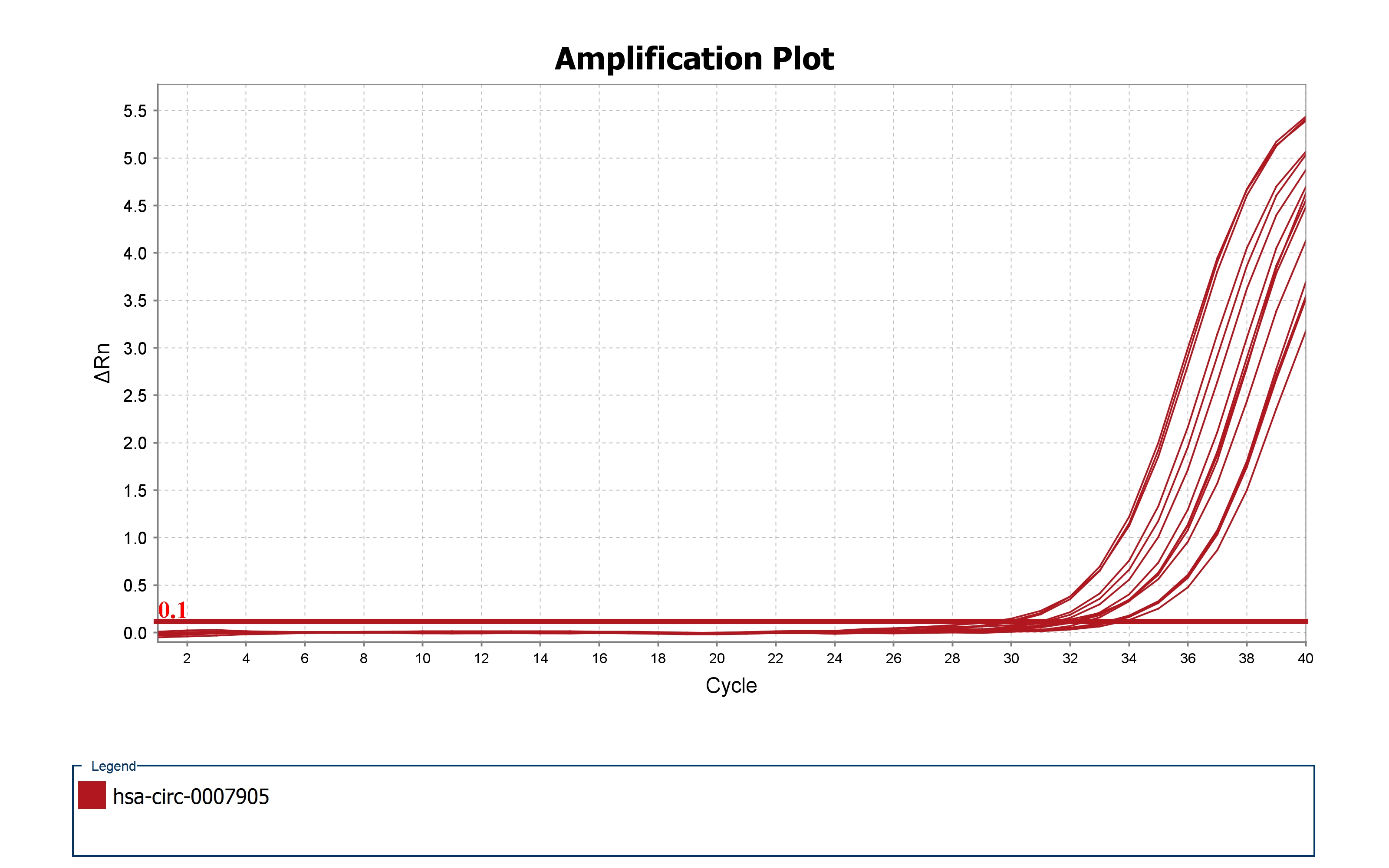

Supplement: Supplemental Information 5 [file peerj-11-14863-s005.zip › Raw data/Fig 3D/Raw data/Amplification Plot hsa-circ-0007905.jpg]

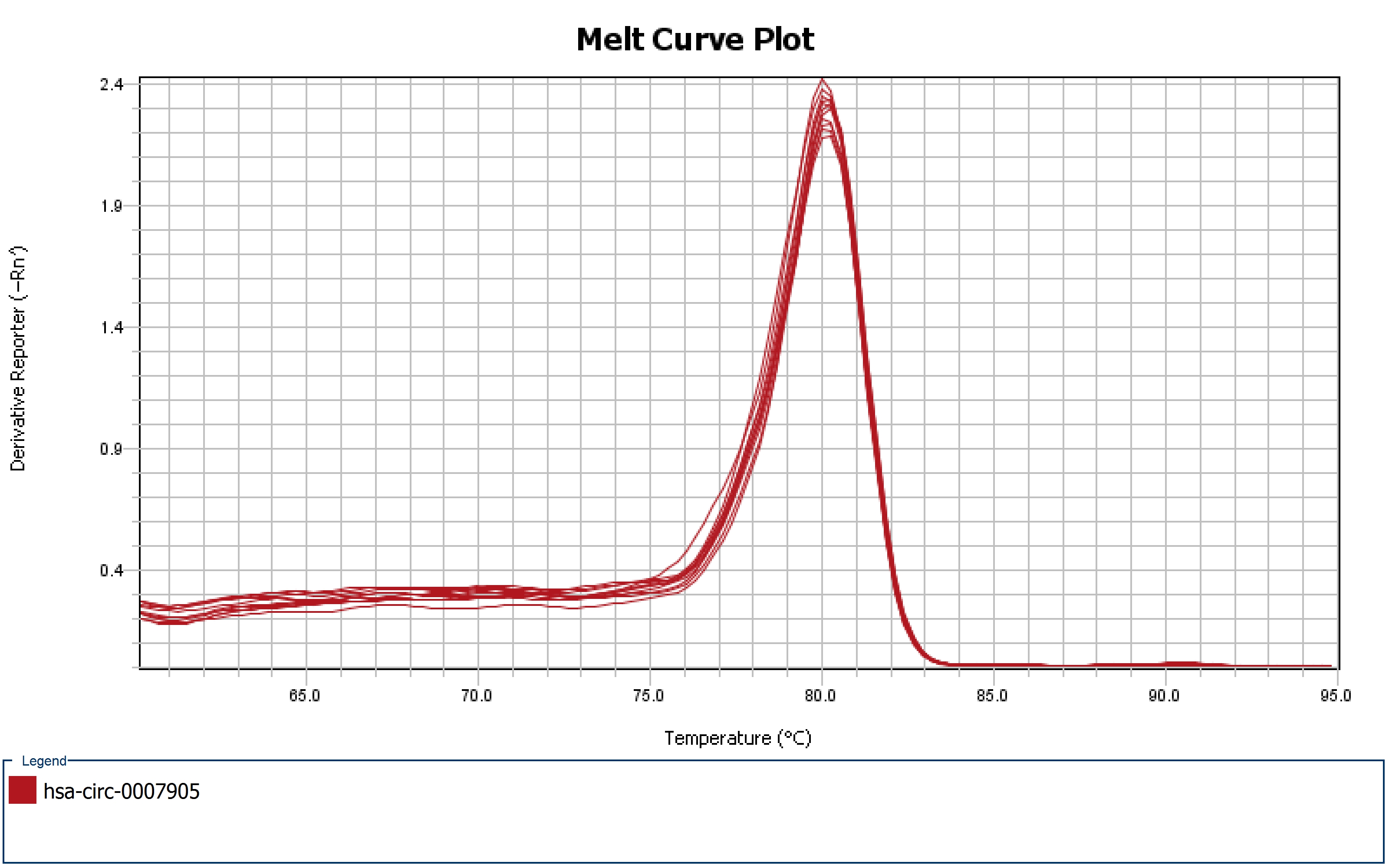

Supplement: Supplemental Information 5 [file peerj-11-14863-s005.zip › Raw data/Fig 3D/Raw data/Melt Curve Plot hsa-circ-0007905.jpg]

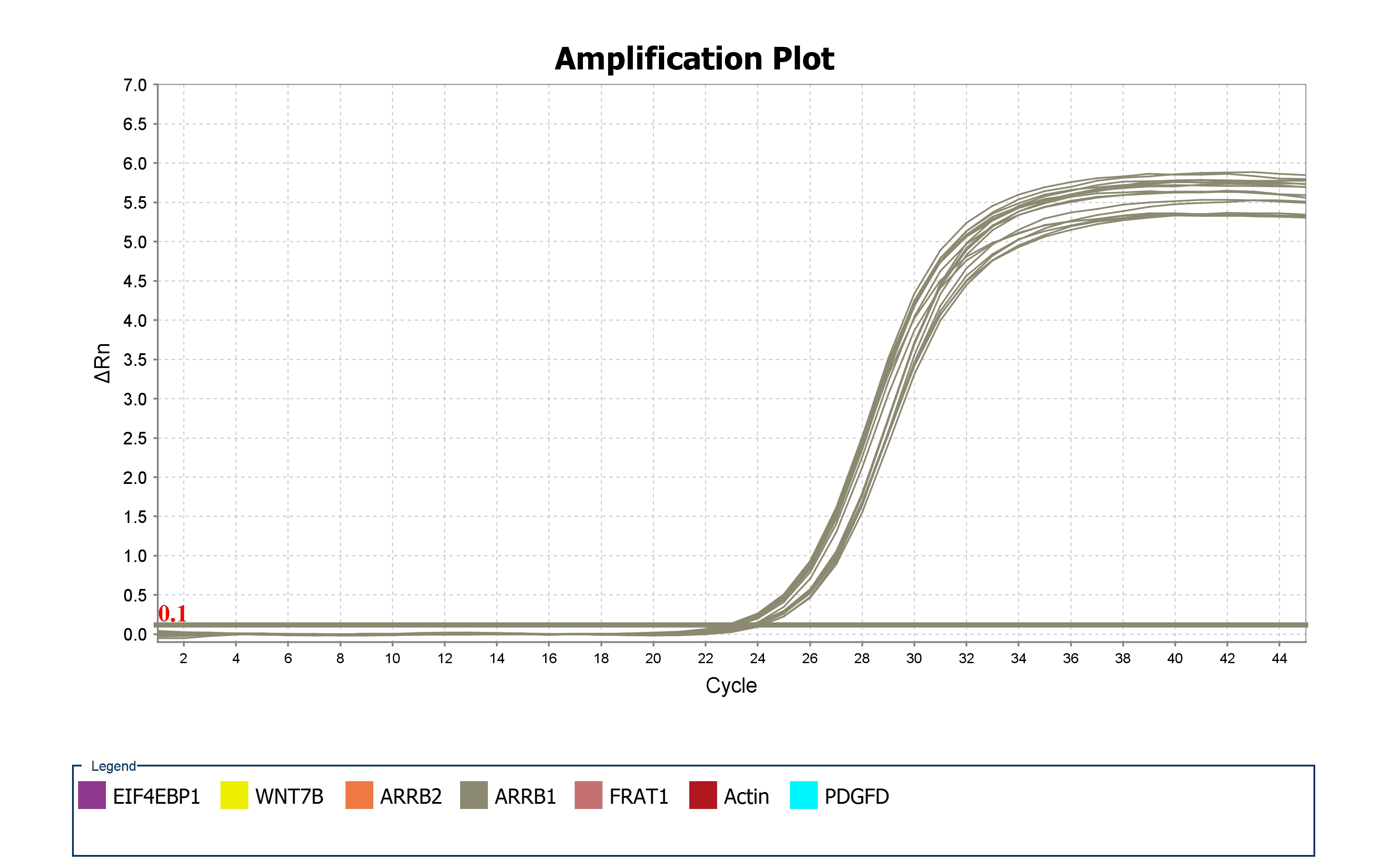

Supplement: Supplemental Information 5 [file peerj-11-14863-s005.zip › Raw data/Fig 4E/Raw data/Amplification Plot ARRB1.jpg]

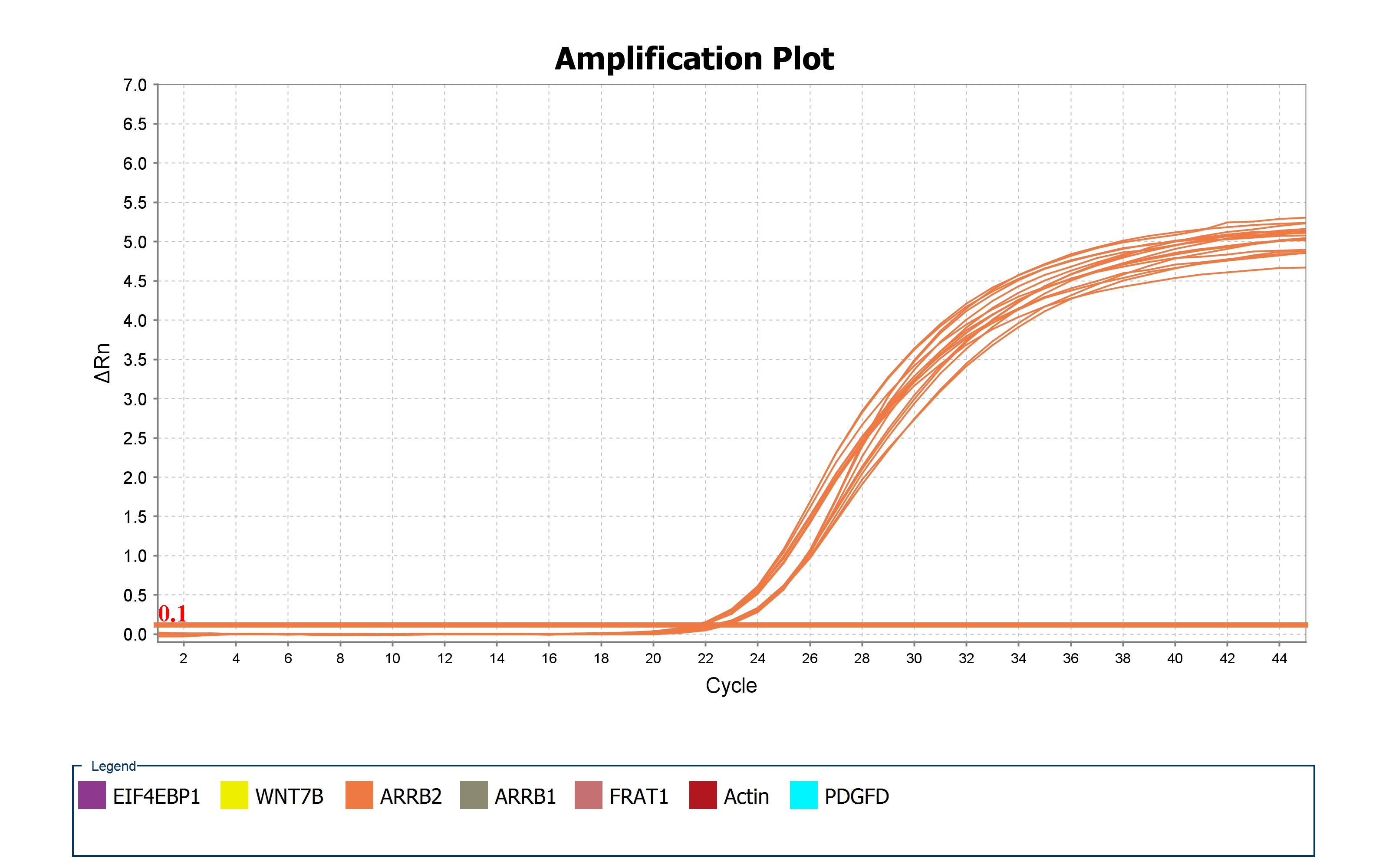

Supplement: Supplemental Information 5 [file peerj-11-14863-s005.zip › Raw data/Fig 4E/Raw data/Amplification Plot ARRB2.jpg]

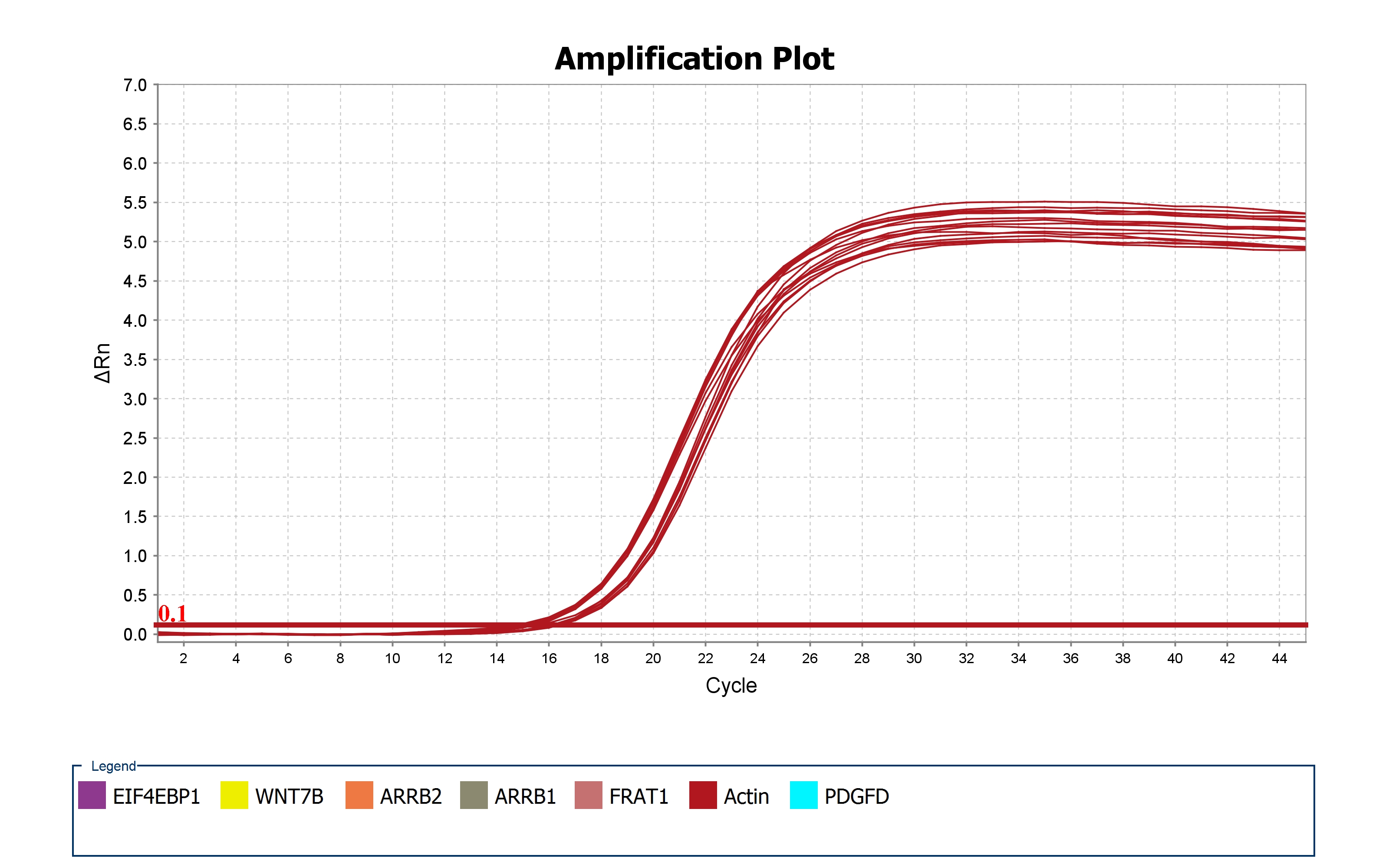

Supplement: Supplemental Information 5 [file peerj-11-14863-s005.zip › Raw data/Fig 4E/Raw data/Amplification Plot Actin.jpg]

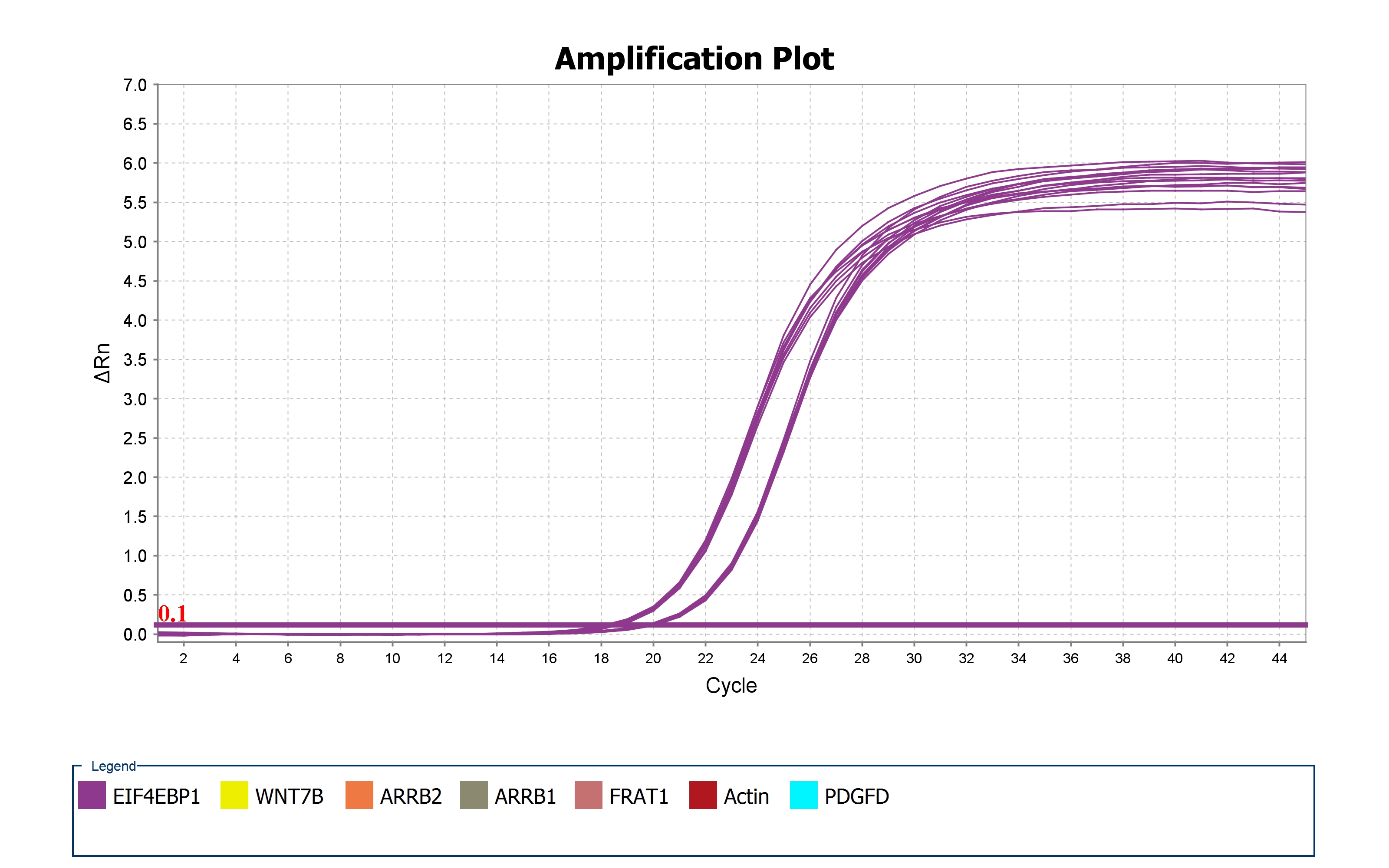

Supplement: Supplemental Information 5 [file peerj-11-14863-s005.zip › Raw data/Fig 4E/Raw data/Amplification Plot EIF4EBP1.jpg]

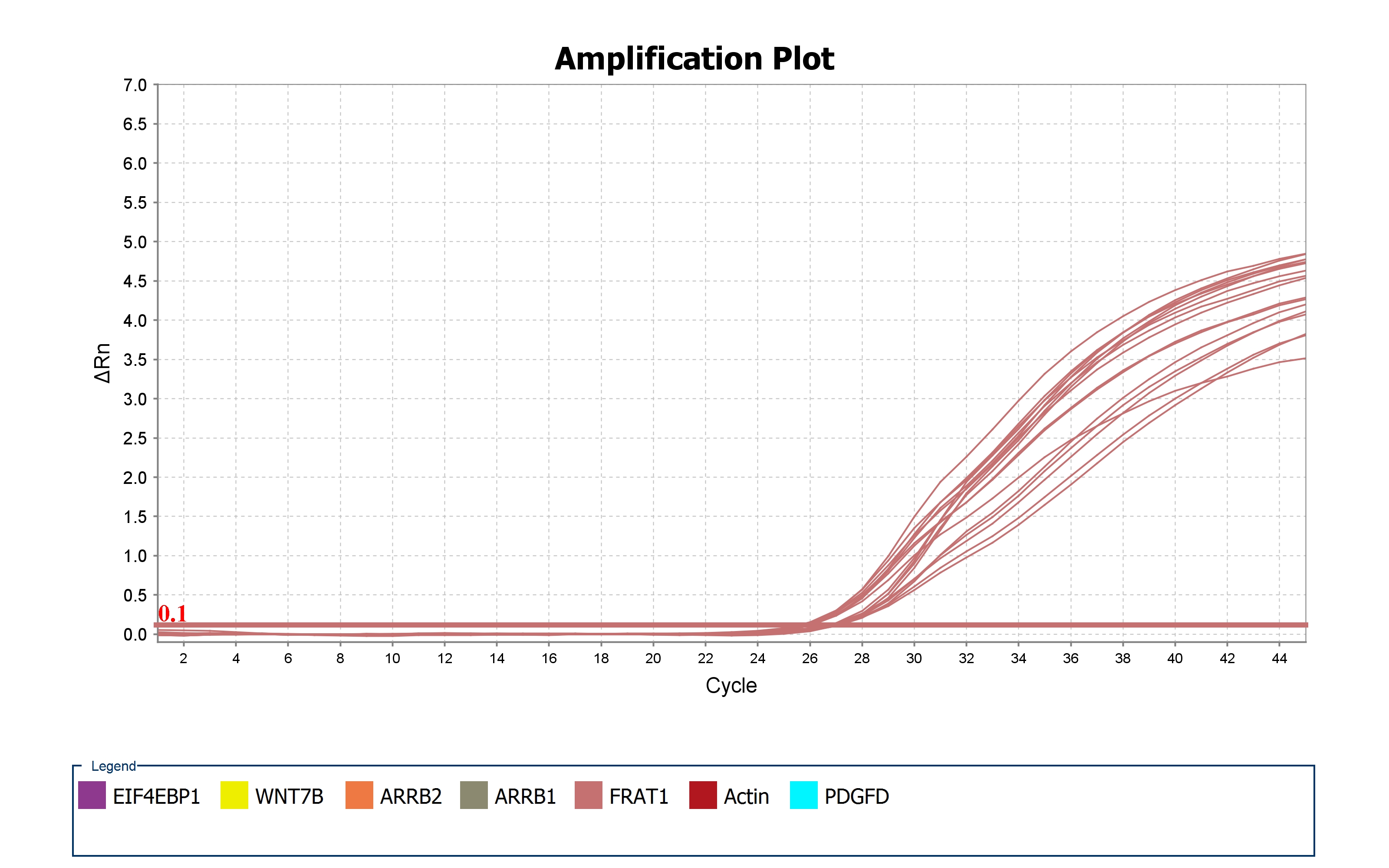

Supplement: Supplemental Information 5 [file peerj-11-14863-s005.zip › Raw data/Fig 4E/Raw data/Amplification Plot FRAT1.jpg]

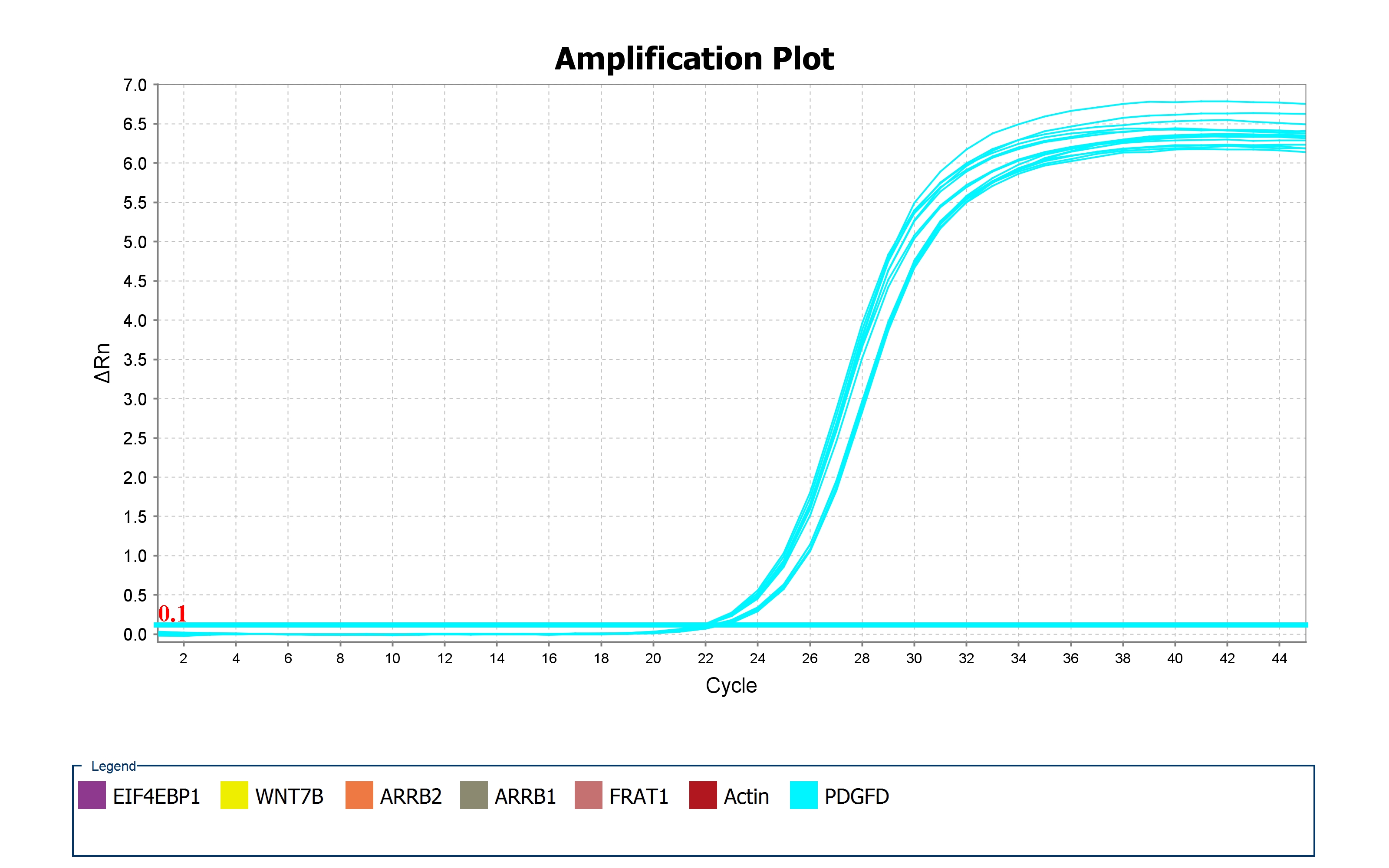

Supplement: Supplemental Information 5 [file peerj-11-14863-s005.zip › Raw data/Fig 4E/Raw data/Amplification Plot PDGFD.jpg]

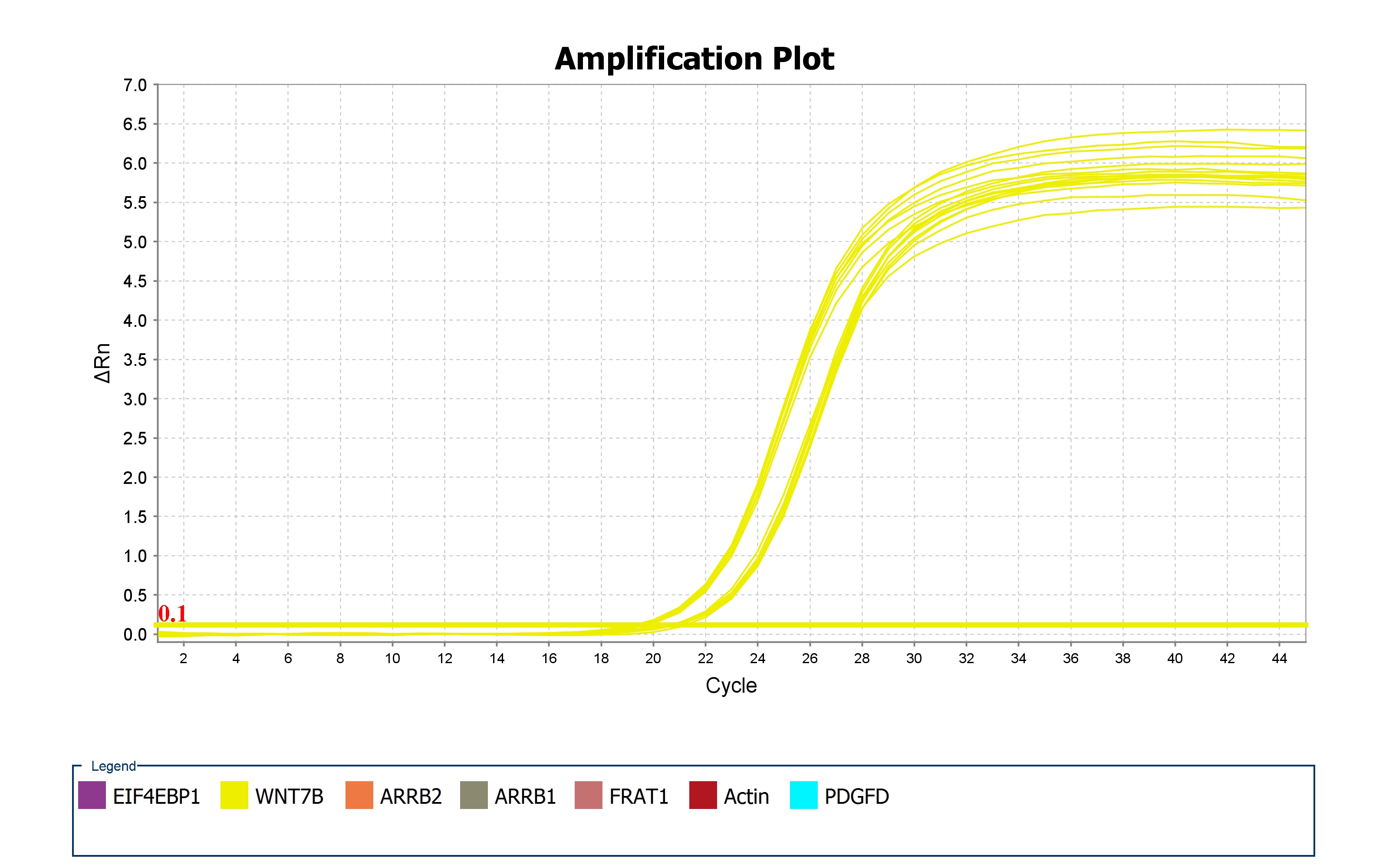

Supplement: Supplemental Information 5 [file peerj-11-14863-s005.zip › Raw data/Fig 4E/Raw data/Amplification Plot WNT7B.jpg]

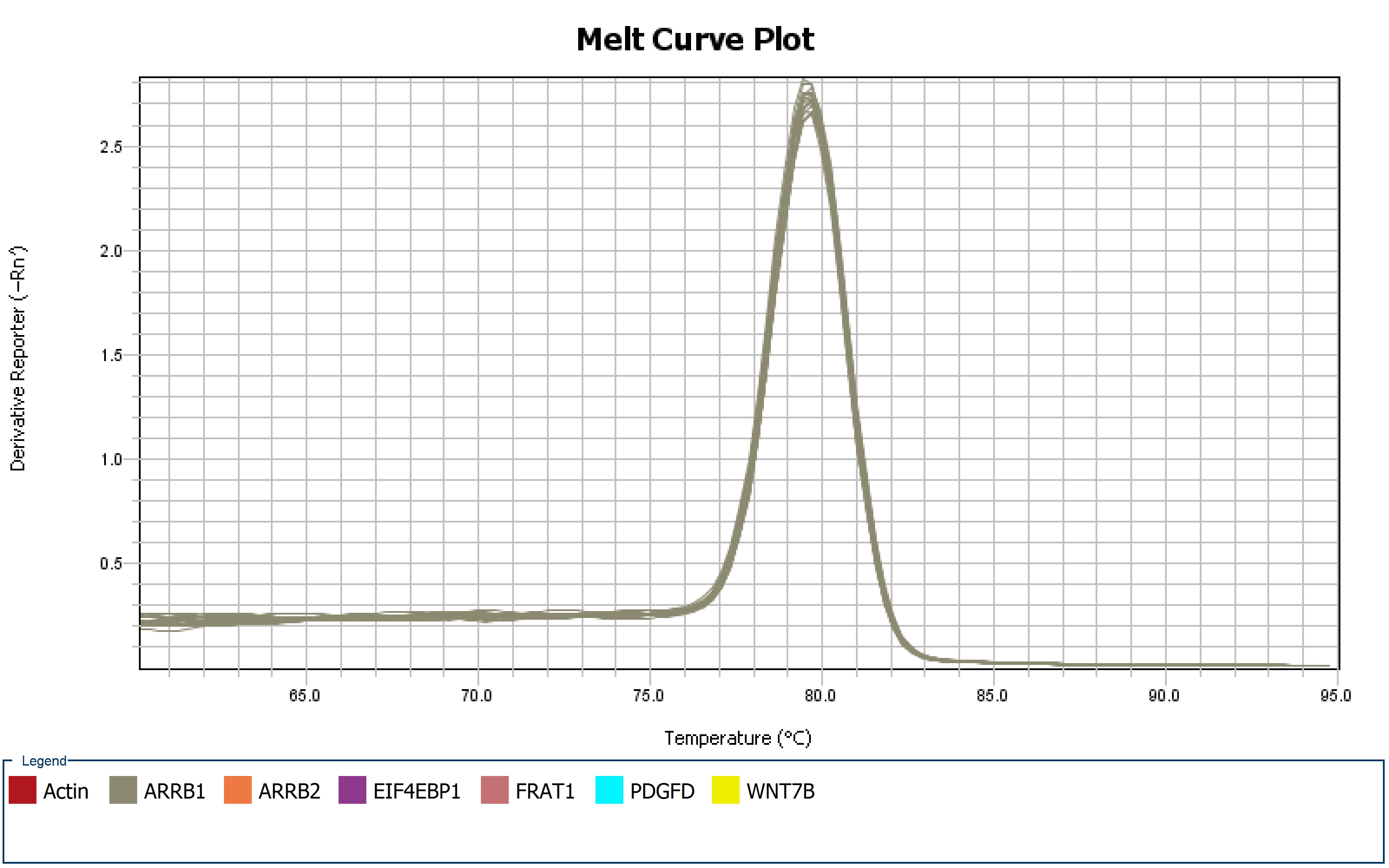

Supplement: Supplemental Information 5 [file peerj-11-14863-s005.zip › Raw data/Fig 4E/Raw data/Melt Curve Plot ARRB1.jpg]

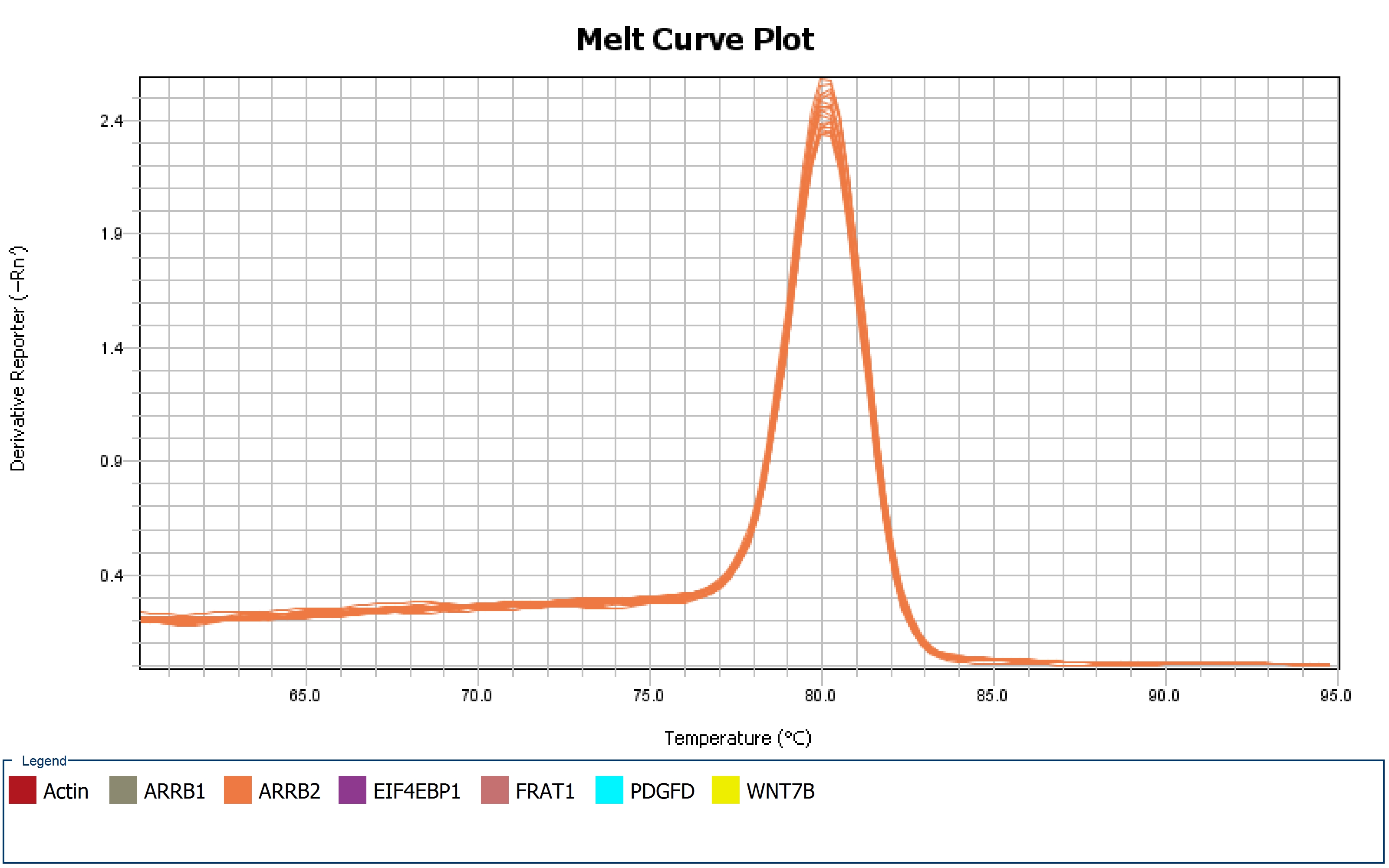

Supplement: Supplemental Information 5 [file peerj-11-14863-s005.zip › Raw data/Fig 4E/Raw data/Melt Curve Plot ARRB2.jpg]

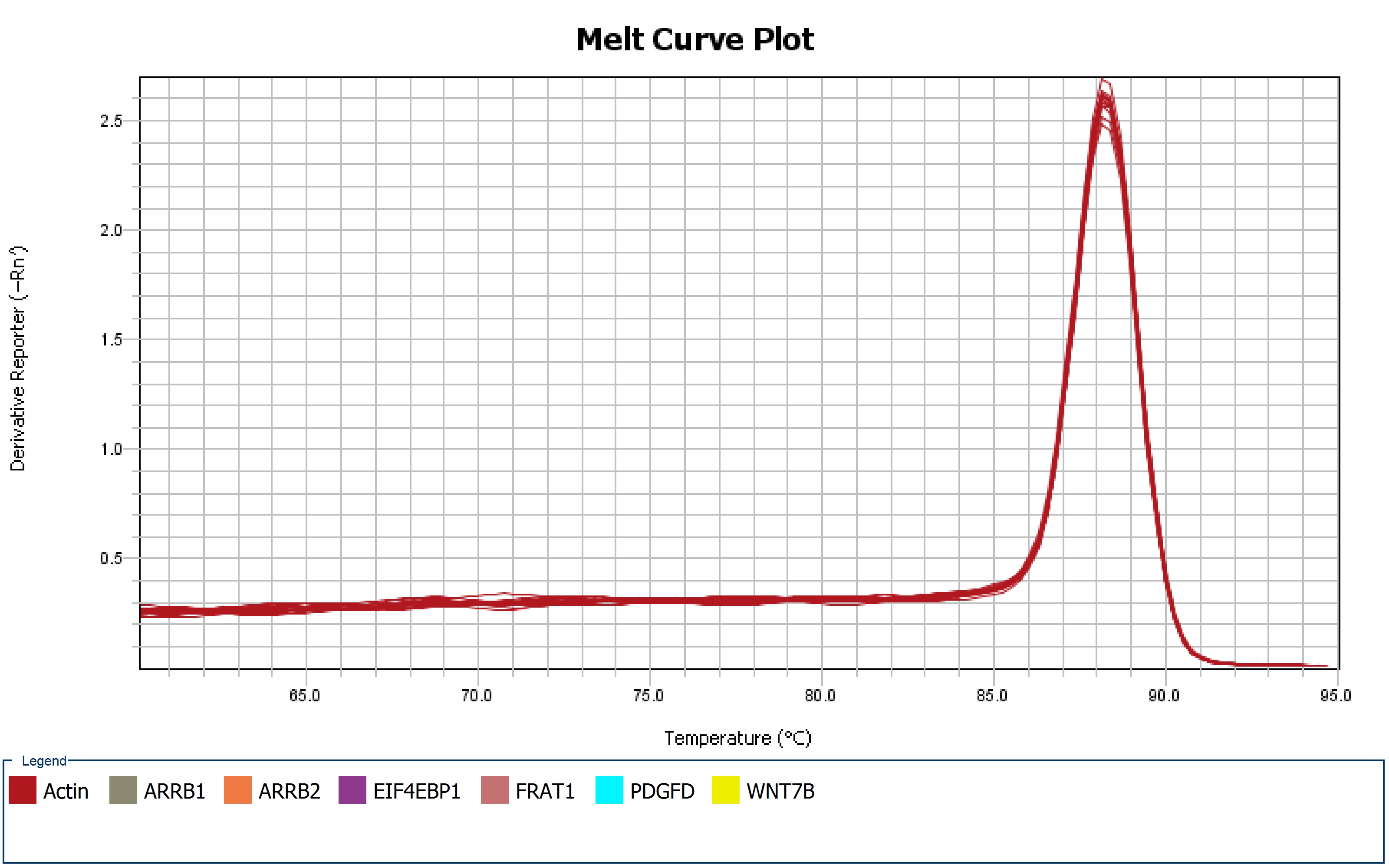

Supplement: Supplemental Information 5 [file peerj-11-14863-s005.zip › Raw data/Fig 4E/Raw data/Melt Curve Plot Actin.jpg]

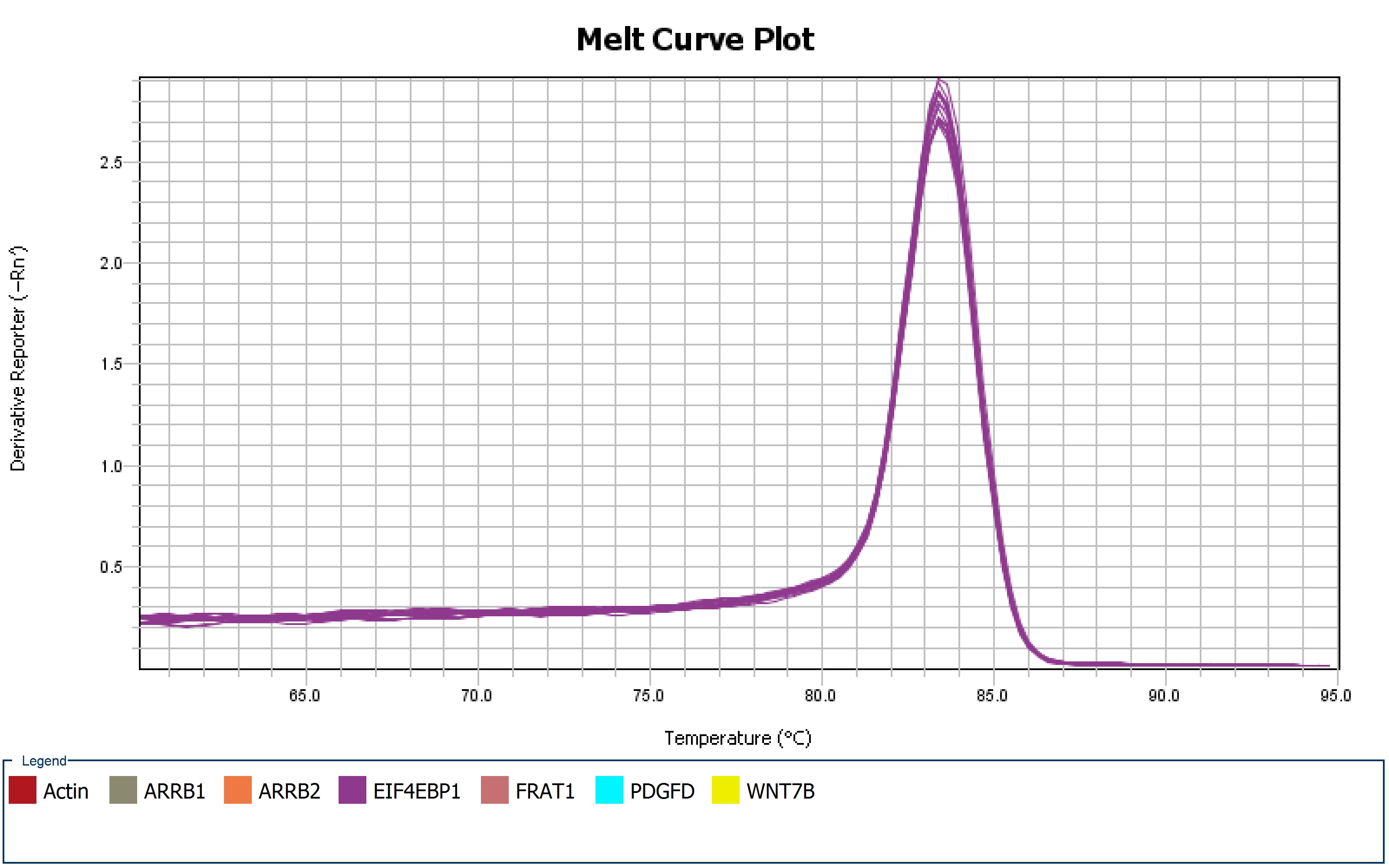

Supplement: Supplemental Information 5 [file peerj-11-14863-s005.zip › Raw data/Fig 4E/Raw data/Melt Curve Plot EIF4EBP1.jpg]

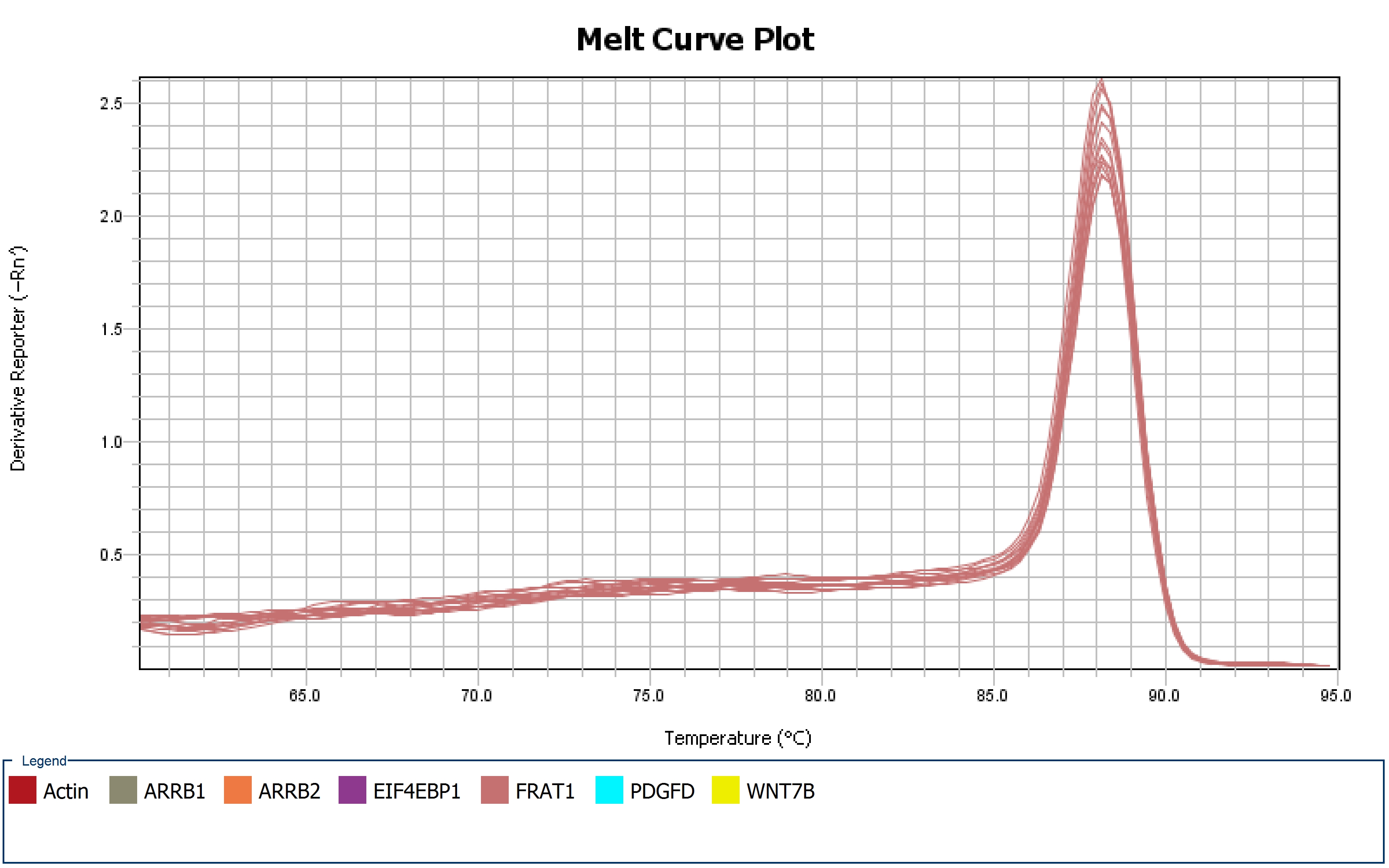

Supplement: Supplemental Information 5 [file peerj-11-14863-s005.zip › Raw data/Fig 4E/Raw data/Melt Curve Plot FRAT1.jpg]

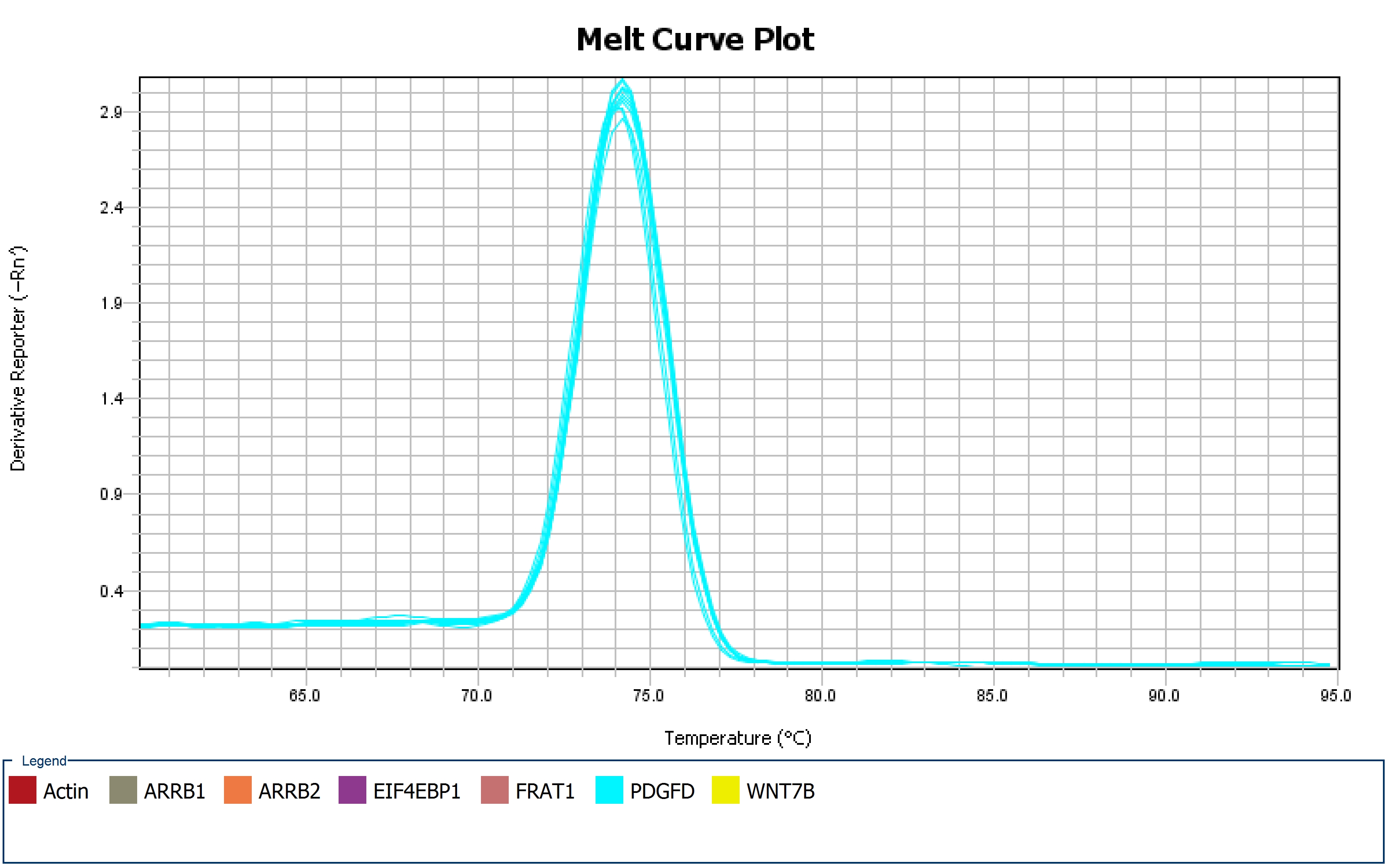

Supplement: Supplemental Information 5 [file peerj-11-14863-s005.zip › Raw data/Fig 4E/Raw data/Melt Curve Plot PDGFD.jpg]

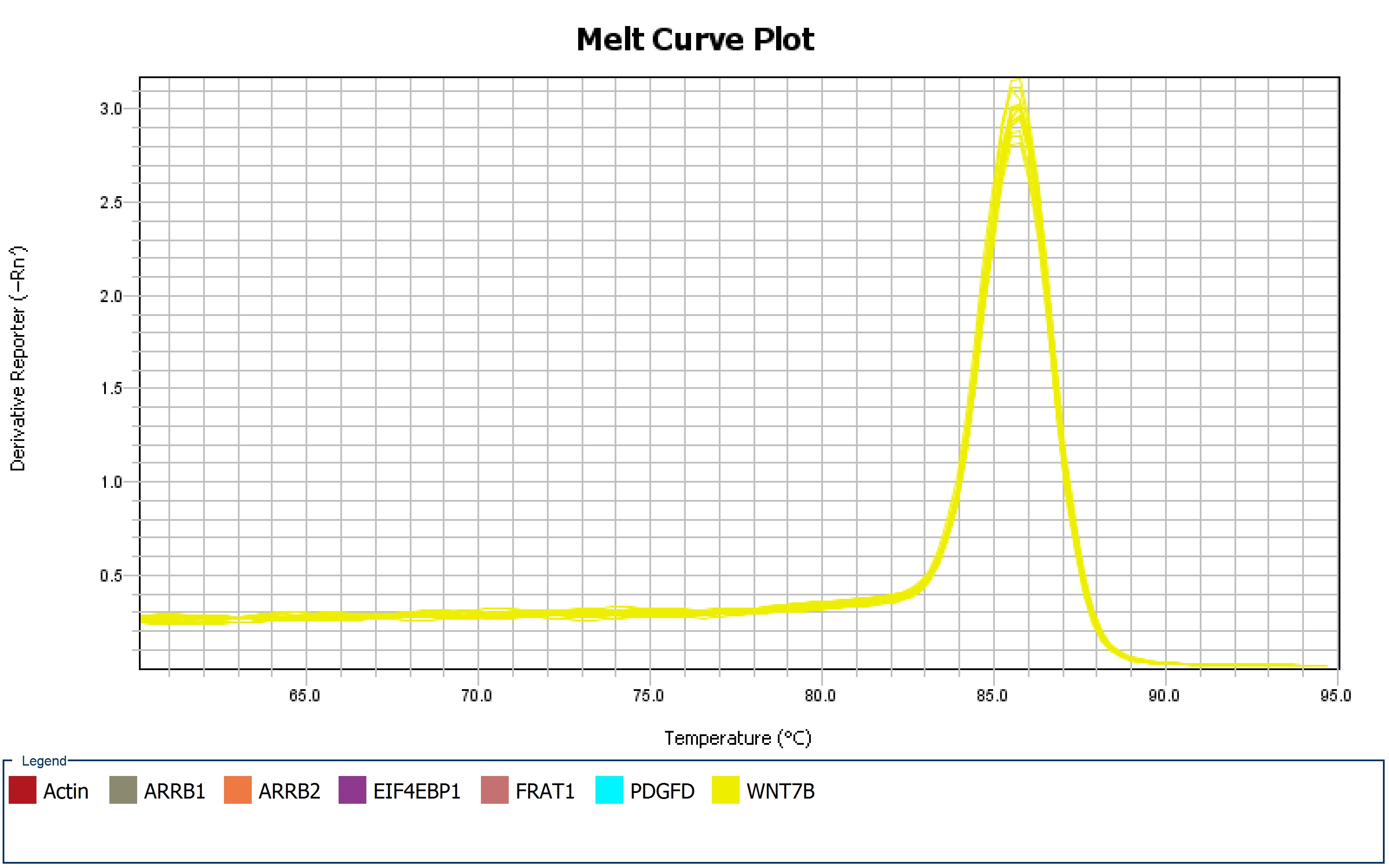

Supplement: Supplemental Information 5 [file peerj-11-14863-s005.zip › Raw data/Fig 4E/Raw data/Melt Curve Plot WNT7B.jpg]

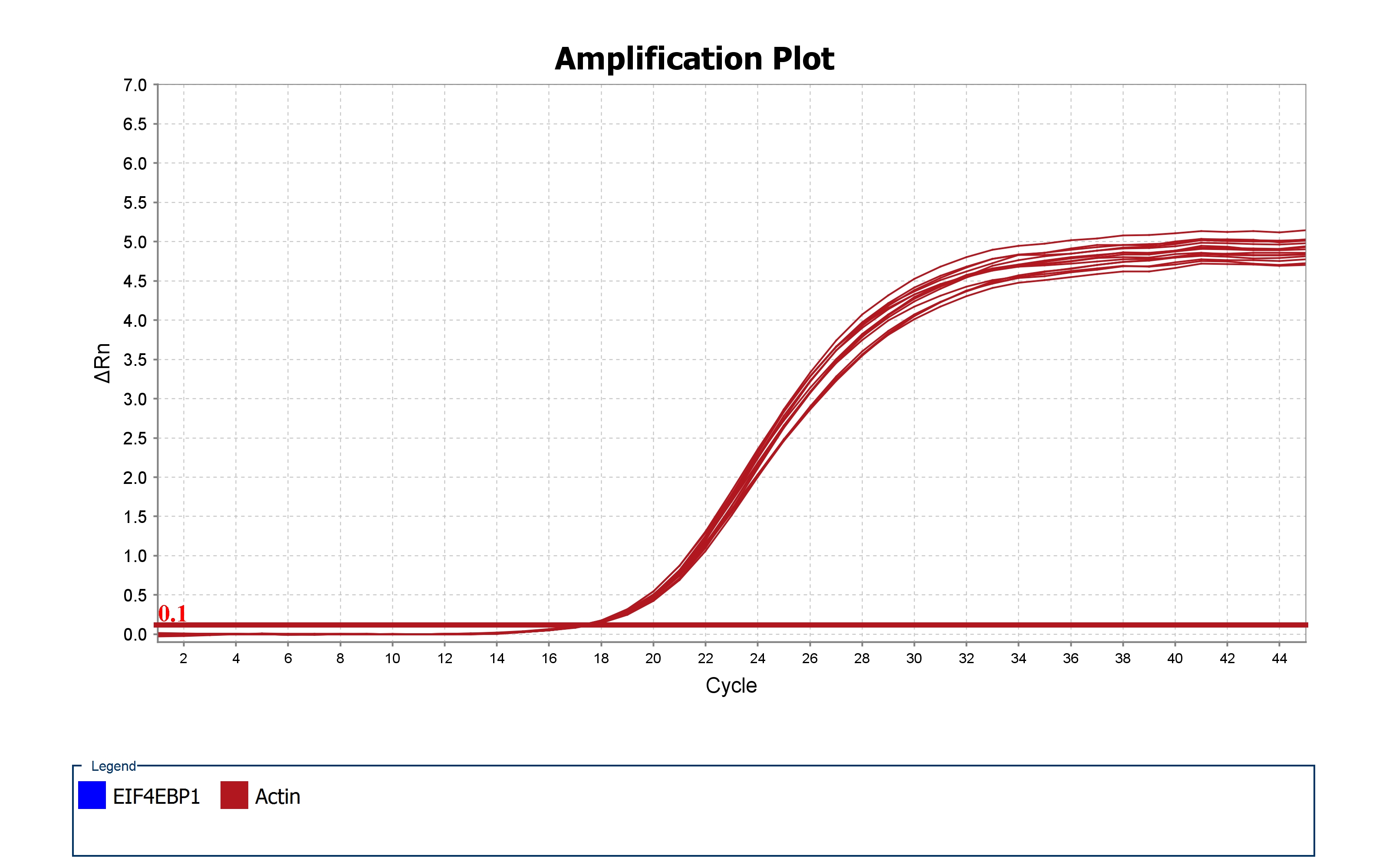

Supplement: Supplemental Information 5 [file peerj-11-14863-s005.zip › Raw data/Fig 5A/Raw data/Amplification Plot Actin.jpg]

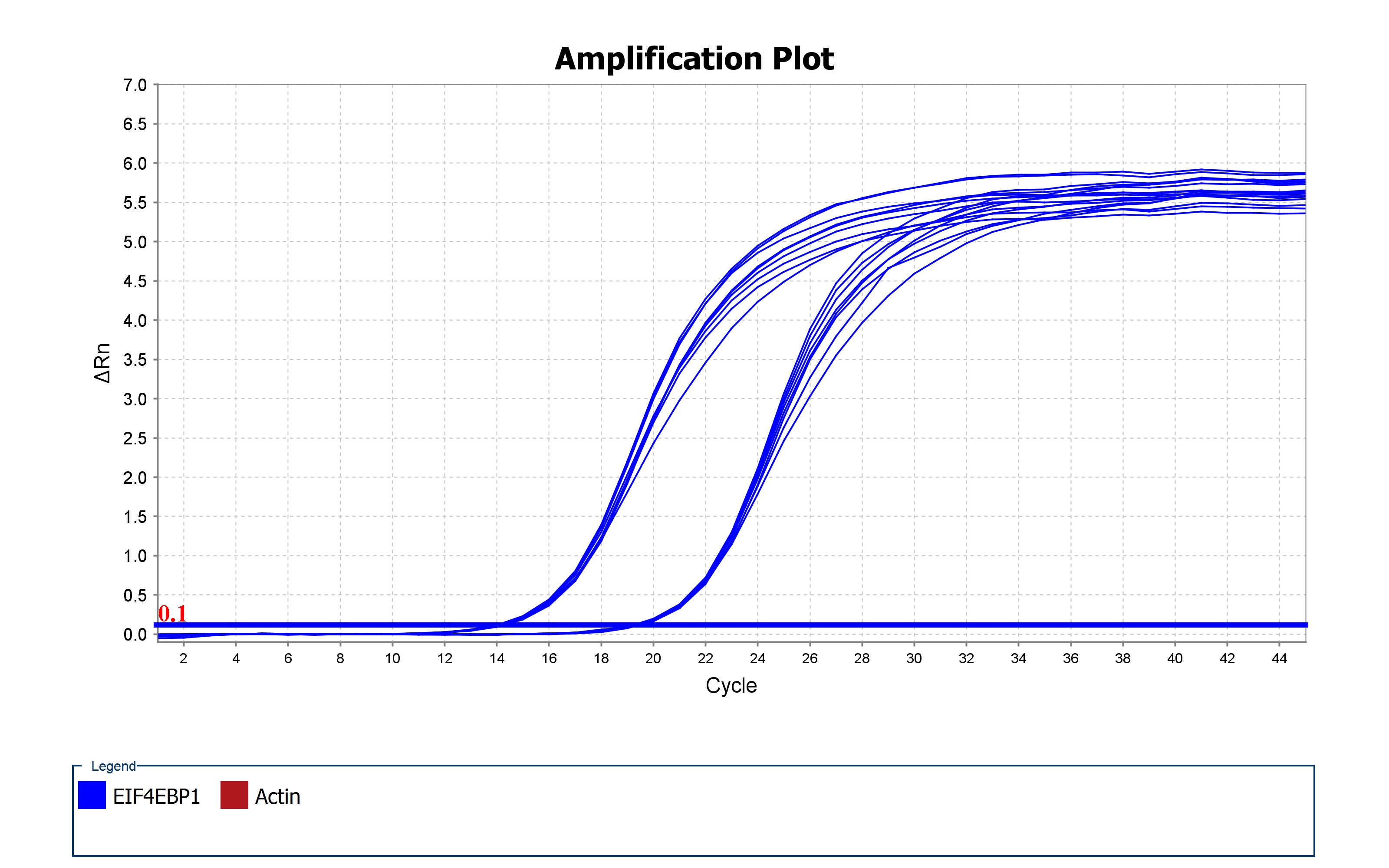

Supplement: Supplemental Information 5 [file peerj-11-14863-s005.zip › Raw data/Fig 5A/Raw data/Amplification Plot EIF4EBP1.jpg]

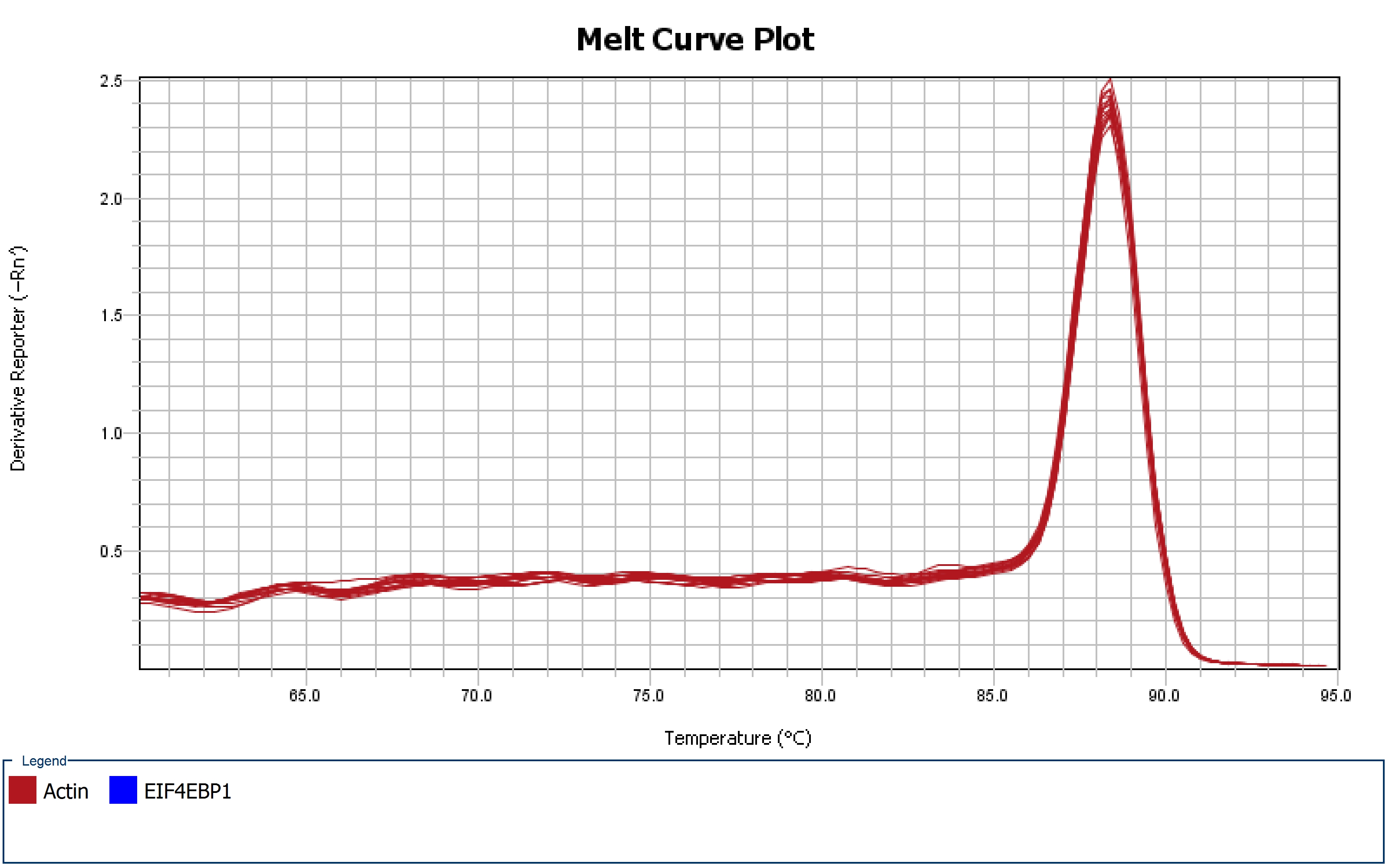

Supplement: Supplemental Information 5 [file peerj-11-14863-s005.zip › Raw data/Fig 5A/Raw data/Melt Curve Plot Actin.jpg]

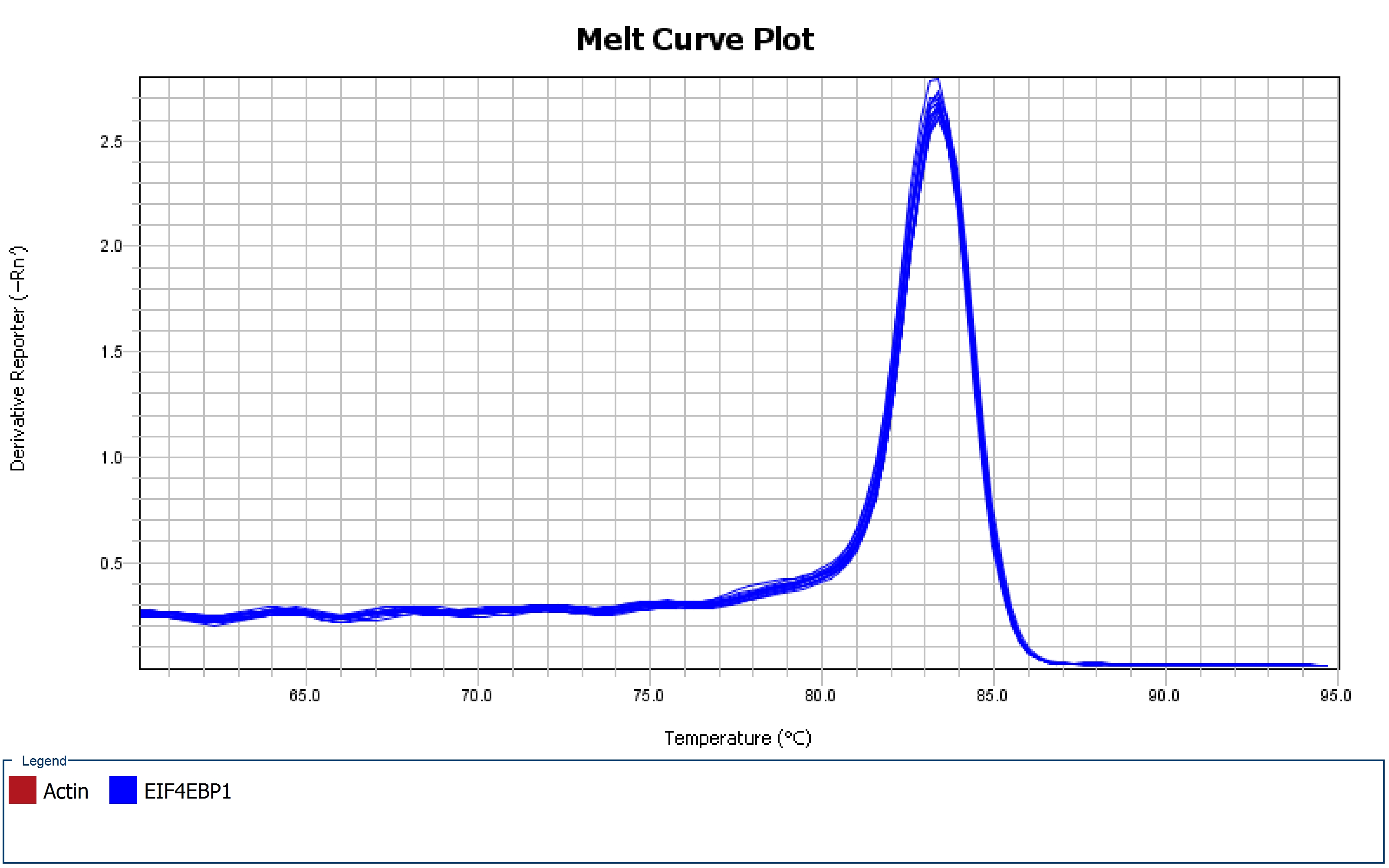

Supplement: Supplemental Information 5 [file peerj-11-14863-s005.zip › Raw data/Fig 5A/Raw data/Melt Curve Plot EIF4EBP1.jpg]

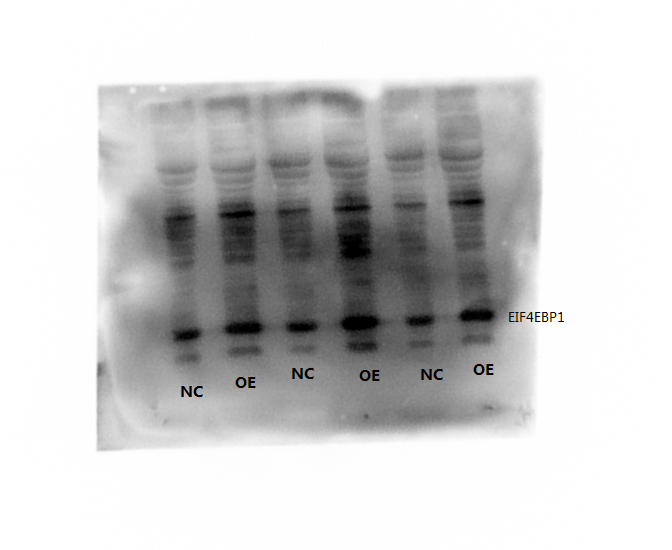

Supplement: Supplemental Information 5 [file peerj-11-14863-s005.zip › Raw data/Fig 5B/5B EIF4EBP1.tif]

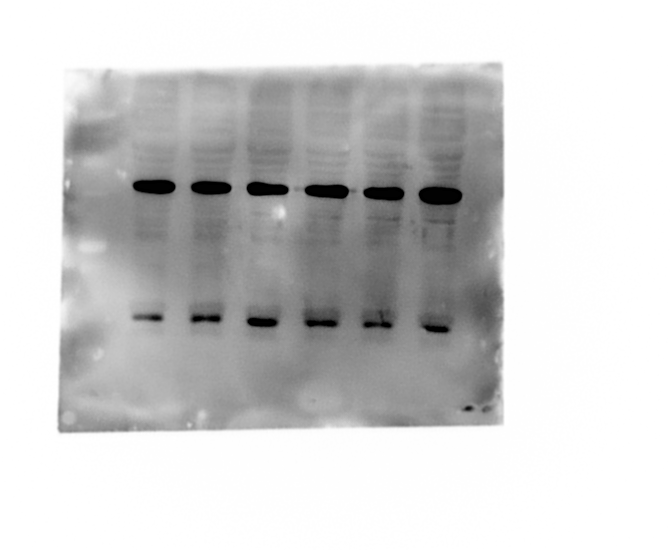

Supplement: Supplemental Information 5 [file peerj-11-14863-s005.zip › Raw data/Fig 5B/GAPDH.tif]

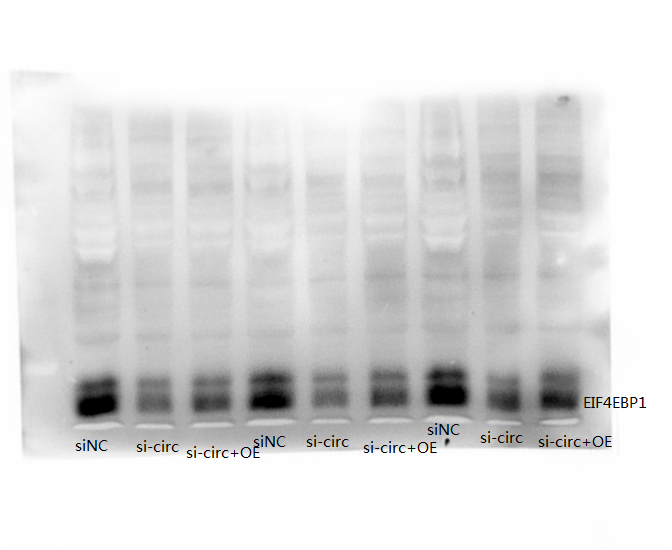

Supplement: Supplemental Information 5 [file peerj-11-14863-s005.zip › Raw data/Fig 5E/5E EIF4EBP1.tif]

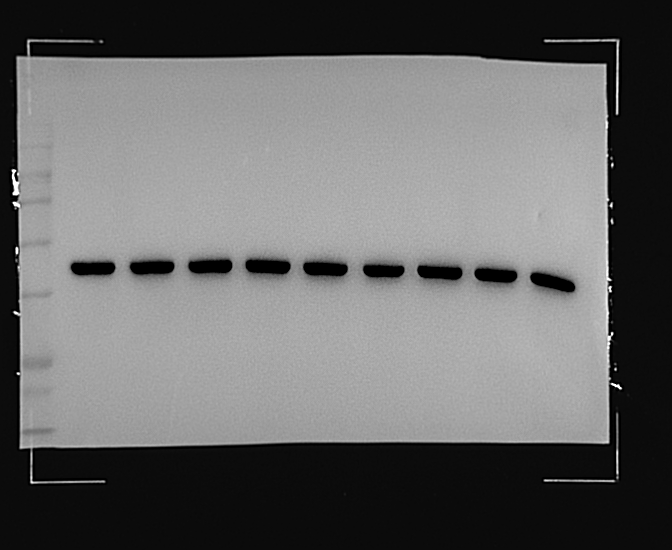

Supplement: Supplemental Information 5 [file peerj-11-14863-s005.zip › Raw data/Fig 5E/GAPDH.tif]

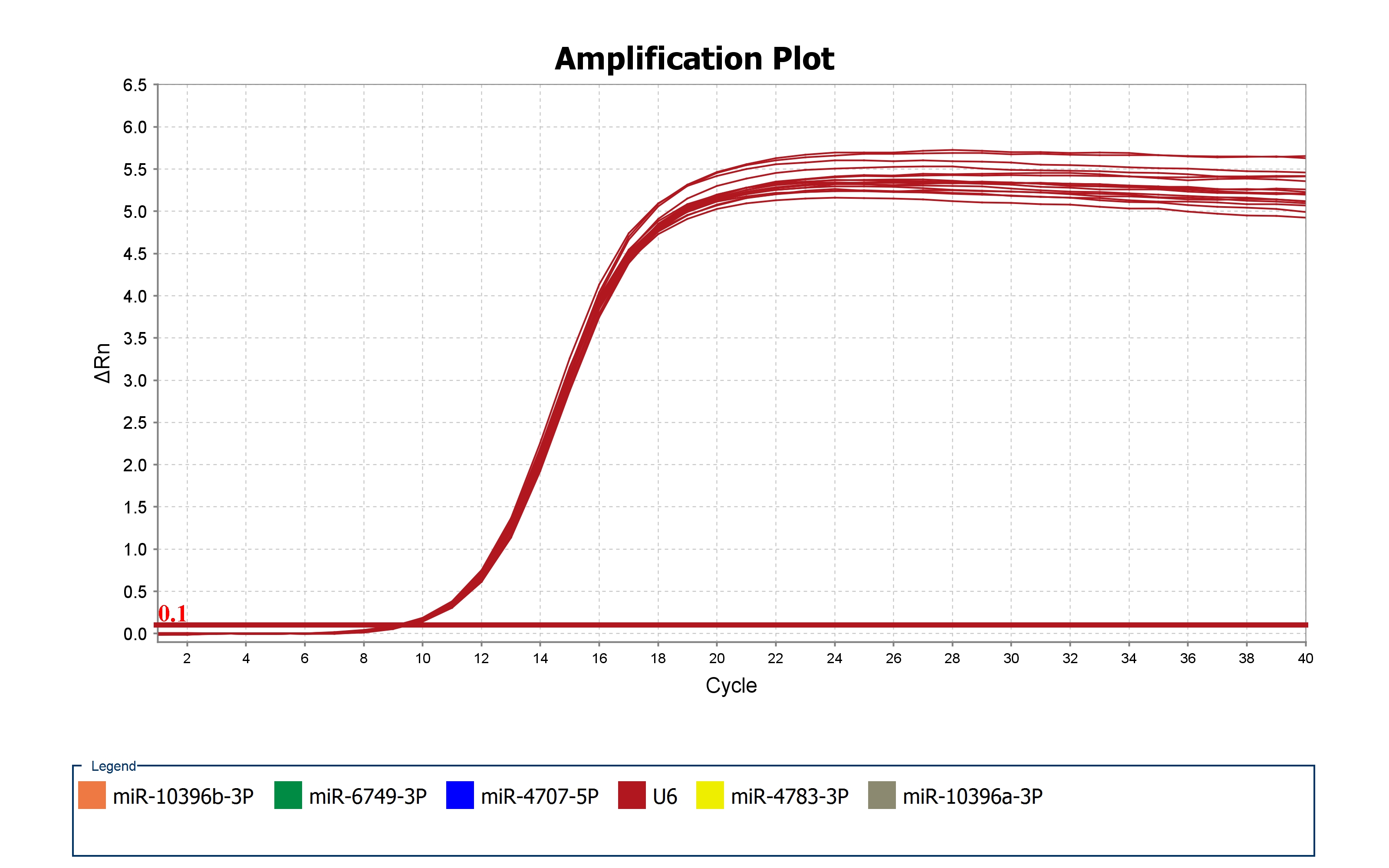

Supplement: Supplemental Information 5 [file peerj-11-14863-s005.zip › Raw data/Fig 6D/Raw data/Amplification Plot U6.jpg]

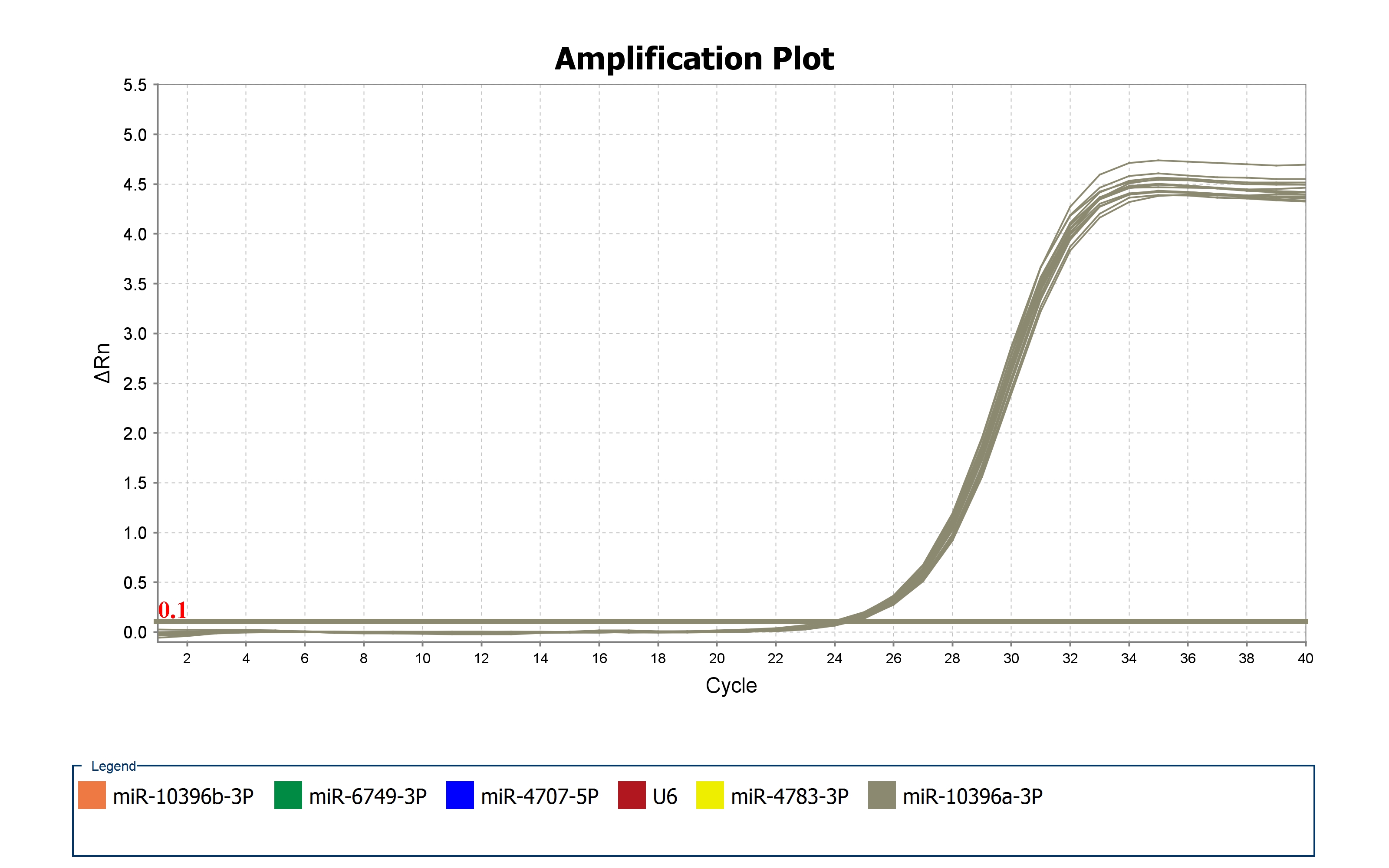

Supplement: Supplemental Information 5 [file peerj-11-14863-s005.zip › Raw data/Fig 6D/Raw data/Amplification Plot miR-10396a-3P.jpg]

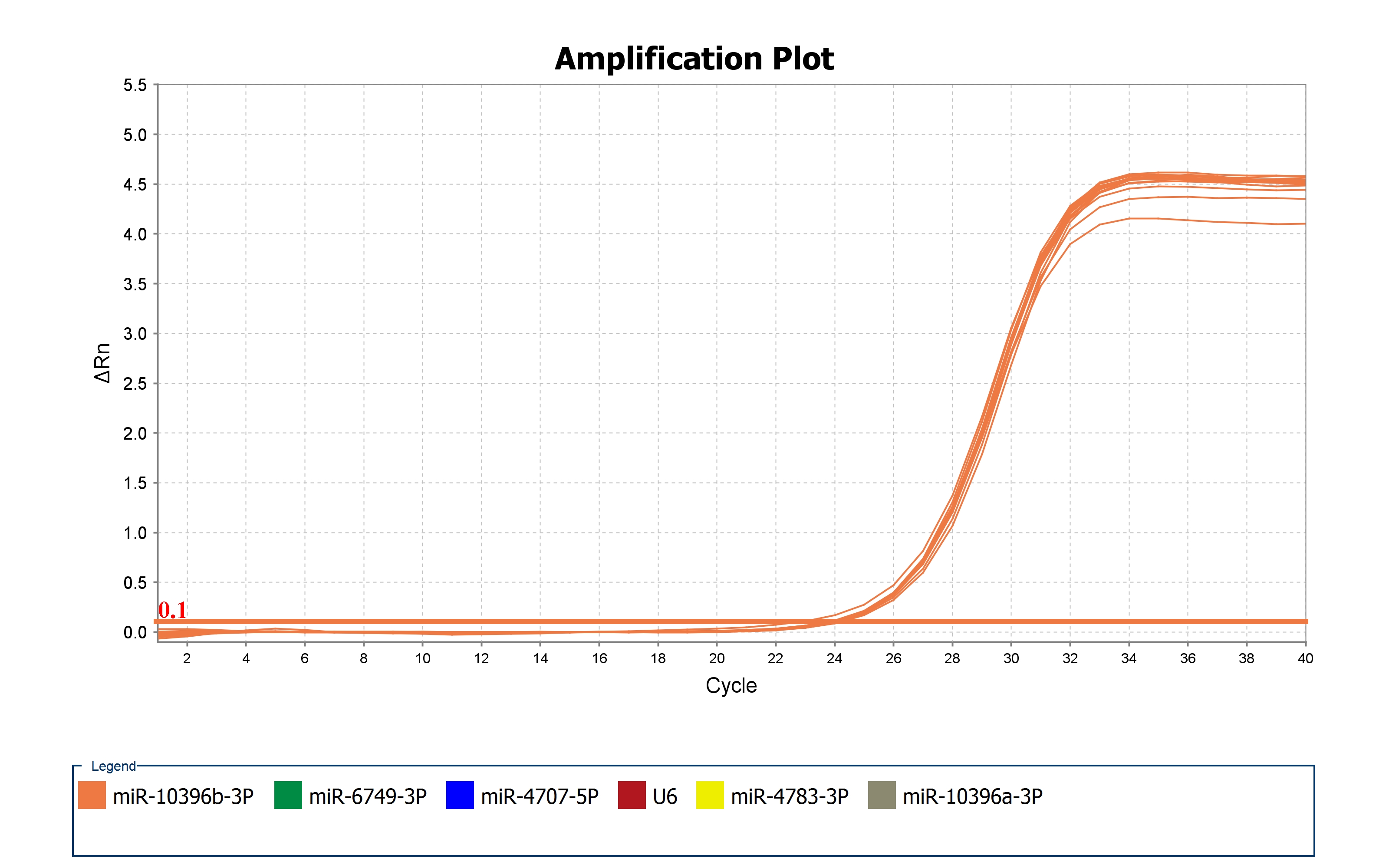

Supplement: Supplemental Information 5 [file peerj-11-14863-s005.zip › Raw data/Fig 6D/Raw data/Amplification Plot miR-10396b-3P.jpg]

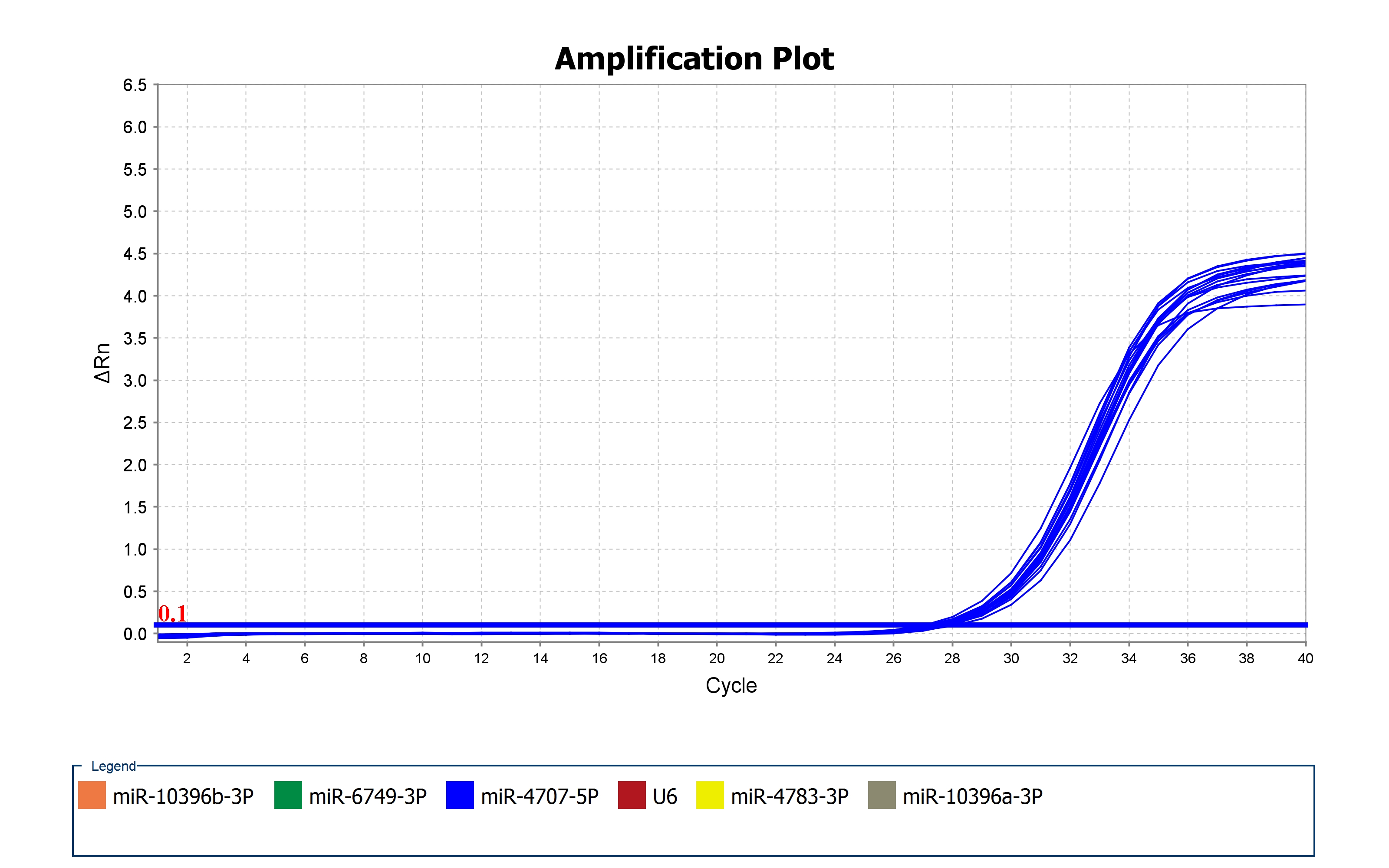

Supplement: Supplemental Information 5 [file peerj-11-14863-s005.zip › Raw data/Fig 6D/Raw data/Amplification Plot miR-4707-5P.jpg]

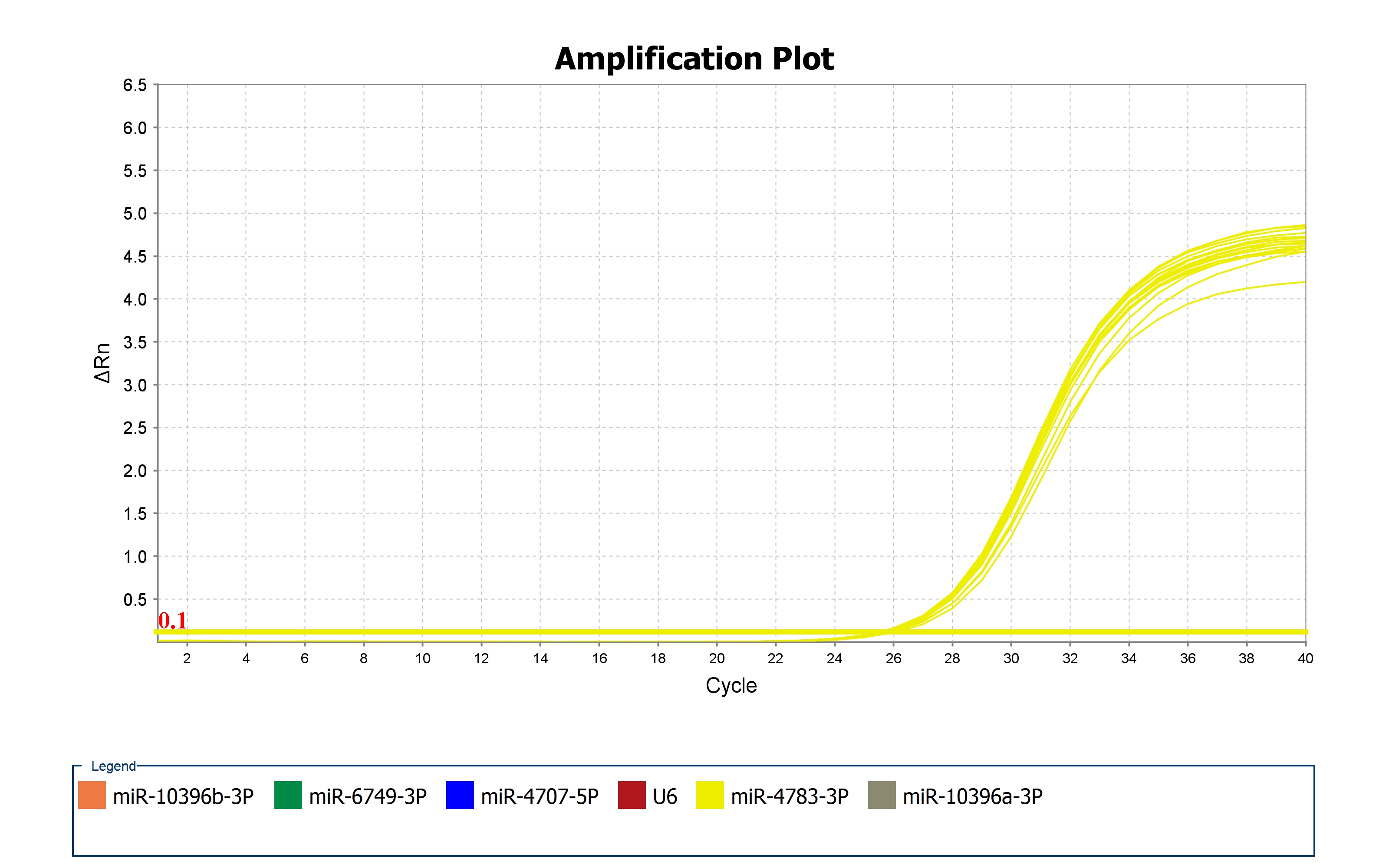

Supplement: Supplemental Information 5 [file peerj-11-14863-s005.zip › Raw data/Fig 6D/Raw data/Amplification Plot miR-4783-3P.jpg]

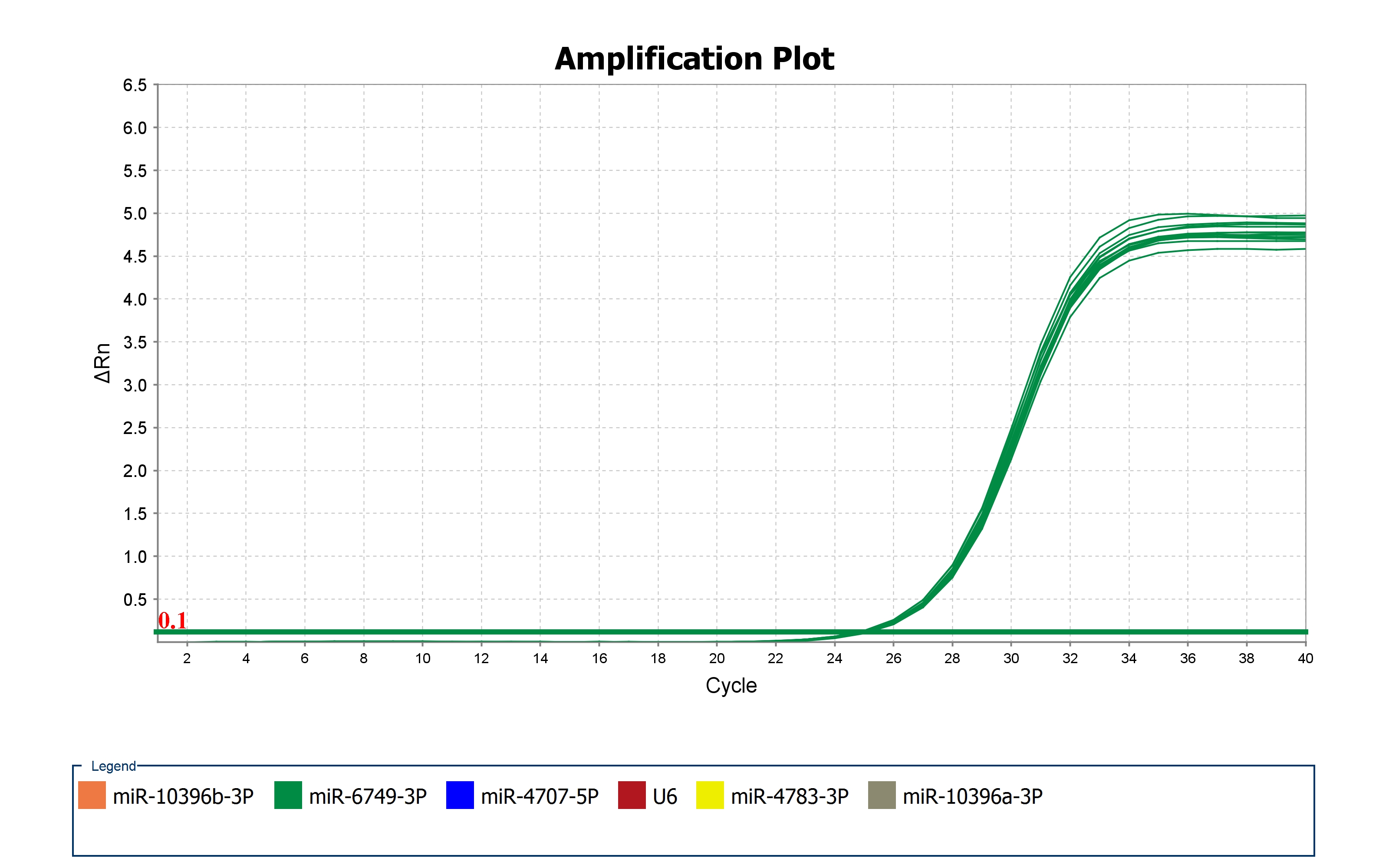

Supplement: Supplemental Information 5 [file peerj-11-14863-s005.zip › Raw data/Fig 6D/Raw data/Amplification Plot miR-6749-3P.jpg]

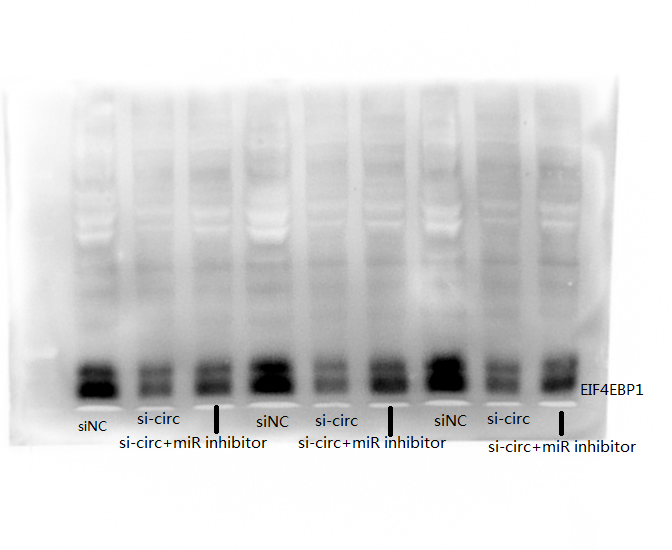

Supplement: Supplemental Information 5 [file peerj-11-14863-s005.zip › Raw data/Fig 6H/6E EIF4EBP1.tif]

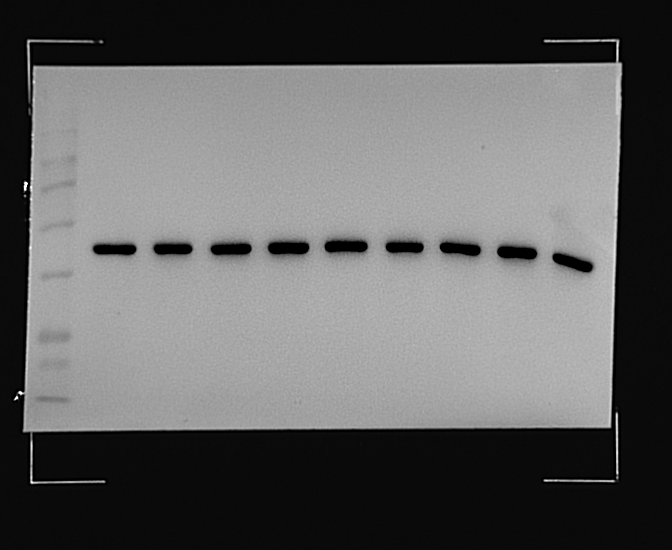

Supplement: Supplemental Information 5 [file peerj-11-14863-s005.zip › Raw data/Fig 6H/GAPDH.tif]
