# Supplementary material for: METTL3-mediated m6A modification of has_circ_0007905 promotes age-related cataract progression through miR-6749-3p/EIF4EBP1
Source: PeerJ. 2023 Mar 6;11:e14863. doi: 10.7717/peerj.14863 (PMC9997201; doi:10.7717/peerj.14863)
Supplement: Supplemental Information 3 [file peerj-11-14863-s003.docx]

Supplemental Table 1. Detailed clinical data for each individual human subject

| Sample |  | Sex | Age | Eye | LOCS III | Axial length (mm) |
| --- | --- | --- | --- | --- | --- | --- |
| ARC | 1 | F | 83 | Left eye | NC4 | 22.85 |
|  | 2 | M | 62 | Left eye | NC3 | 23.44 |
|  | 3 | M | 83 | Left eye | NC4 | 24.43 |
|  | 4 | F | 79 | Left eye | NC3 | 25.55 |
|  | 5 | F | 74 | Left eye | NC3 | 22.61 |
|  | 6 | F | 65 | Left eye | NC2 | 23.40 |
|  | 7 | F | 81 | Left eye | NC3 | 23.94 |
|  | 8 | M | 84 | Right eye | NC3 | 23.54 |
|  | 9 | M | 70 | Right eye | NC2 | 25.45 |
|  | 10 | F | 92 | Left eye | NC3 | 23.69 |
| NC | 1 | F | 60 | Right eye | - | 23.24 |
|  | 2 | F | 59 | Right eye | - | 22.43 |

NC in LOCS III: Nuclear Color
