# Supplementary material for: METTL3-mediated m6A modification of has_circ_0007905 promotes age-related cataract progression through miR-6749-3p/EIF4EBP1
Source: PeerJ. 2023 Mar 6;11:e14863. doi: 10.7717/peerj.14863 (PMC9997201; doi:10.7717/peerj.14863)
Supplement: Supplemental Information 4 [file peerj-11-14863-s004.docx]

Supplemental Table 2. The primers used in this study.

| Gene | Primers (5’- 3’) |
| --- | --- |
| Actin-F | AGCACAGAGCCTCGCCTTTG |
| Actin-R | CTTCTGACCCATGCCCACCA |
| hsa_circ_0007905-F | GGTGCTGAAGAACATGTCCC |
| hsa_circ_0007905-R | GCTAATGCCTGCACAGATGA |
| hsa_circ_0035228-F | GAGTGAGTTCTCTTGCTGGAT |
| hsa_circ_0035228-R | ATGCACATCTGTTCCACTCG |
| hsa_circ_0065244-F | CTCCCTACCCTCTGATGCAC |
| hsa_circ_0065244-R | AAAGGCATGGACCCTCATCA |
| hsa_circ_0003949-F | GACTGTCACCATCTCCAAGC |
| hsa_circ_0003949-R | CAGCTGCAAATTTTCGACGC |
| hsa_circ_0022997-F | AAGGCCCTGCTCAATGTCAT |
| hsa_circ_0022997-R | TACTTGGGGTGCTTGGAGTC |
| METTL3-F | TTTCCGGTTAGCCTTCGGGG |
| METTL3-R | CATCCTAGTCTCCCAGCCCT |
| METTL14-F | CAACTACGGAAAACCCTCAACT |
| METTL14-R | CAGCAAGAGAAGTGACAGCATAA |
| WTAP-F | CCAAGAAGGTTCGATTGAGTGA |
| WTAP-R | ACTCTCTTAGGCCAGTTACATCA |
| FTO-F | AGTTATAGCTGTGAAGGCCCT |
| FTO-R | TGTCCCATGAGATCTTAAAACCA |
| ALKBH5-F | CGTGACTGTGCTCAGTGGAT |
| ALKBH5-R | GAGCTGCTCAGGGACTTTGT |
| WNT7B-F | CGGCCTCATTGTTATGCAGGT |
| WNT7B-R | CGGCCTCATTGTTATGCAGGT |
| ARRB1-F | GCTTGCGGTGTGGACTATGAA |
| ARRB1-R | CTGGGGCATACTGAACCTTCC |
| ARRB2-F | TCCACCAAGACCGTCAAGAA |
| ARRB2-R | TTCGAGTTGAGCCACAGGAC |
| EIF4EBP1-F | CTATGACCGGAAATTCCTGATGG |
| EIF4EBP1-R | CCCGCTTATCTTCTGGGCTA |
| FRAT1-F | TACGTGCAAAGCTTCCCCAA |
| FRAT1-R | TTAGCTGCCAGGCACAAGAA |
| PDGFD-F | ACGGATACAGCTAGTGTTTGACA |
| PDGFD-R | GTCCACACCATCGTCCTCTAATA |
| hsa-miR-4707-5p-RT | GTCGTATCCAGTGCGTGTCGTGGAGTCGGCAATTGCACTGGATACGACCCAGAAC |
| hsa-miR-6749-3p-RT | GTCGTATCCAGTGCGTGTCGTGGAGTCGGCAATTGCACTGGATACGACCTGGGCC |
| hsa-miR-4783-3p-RT | GTCGTATCCAGTGCGTGTCGTGGAGTCGGCAATTGCACTGGATACGACGCAGACG |
| hsa-miR-10396a-3p-RT | GTCGTATCCAGTGCGTGTCGTGGAGTCGGCAATTGCACTGGATACGACCCCGGTC |
| hsa-miR-10396b-3p-RT | GTCGTATCCAGTGCGTGTCGTGGAGTCGGCAATTGCACTGGATACGACGTCCGGT |
| si-has_circ_0007905-1 | UCAUAUGACCAGUGGACAUTT  AUGUCCACUGGUCAUAUGATT |
| si-has_circ_0007905-2 | AUAUGACCAGUGGACAUGATT  UCAUGUCCACUGGUCAUAUTT |
| si-METTL3-Homo-1604 | GGUUGGUGUCAAAGGAAAUTT  AUUUCCUUUGACACCAACCTT |
| si-METTL3-Homo-1086 | GGUGACUGCUCUUUCCUUATT  UAAGGAAAGAGCAGUCACCTT |
| si-METTL3-Homo-968 | GCAAGAAUUCUGUGACUAUTT  AUAGUCACAGAAUUCUUGCTT |
| siRNA-NC | UUCUCCGAACGUGUCACGUTT  ACGUGACACGUUCGGAGAATT |
| hsa-miR-6749-3p inhibitor | CTGGGCCAGGCAGGGGAGGAG |
| U6-F | CGATACAGAGAAGATTAGCATGGC |
| U6-R | AACGCTTCACGAATTTGCGT |
| hsa-miR-4707-5p-F | GCGGGCGGGTTC |
| hsa-miR-6749-3p-F | GCTCCTCCCCTGCCT |
| hsa-miR-4783-3p-F | GTTGGGGCGCGTC |
| hsa-miR-10396a-3p-F | GCCCCGGGCCCT |
| hsa-miR-10396b-3p-F | GCCCCGGGCCCT |
| R | AGTGCGTGTCGTGGAGTCG |

Note: F means forward primers, R means reverse primers, RT means reverse transcription
